# MUC1 Glycopeptide Vaccine Modified with a GalNAc Glycocluster Targets the Macrophage Galactose C-type Lectin on Dendritic Cells to Elicit an Improved Humoral Response

**DOI:** 10.1021/jacs.2c12843

**Published:** 2023-06-06

**Authors:** Adele Gabba, Riem Attariya, Sandra Behren, Christian Pett, Joost C. van der Horst, Hajime Yurugi, Jin Yu, Moritz Urschbach, Juan Sabin, Gabriel Birrane, Edgar Schmitt, Sandra J. van Vliet, Pol Besenius, Ulrika Westerlind, Paul V. Murphy

**Affiliations:** † School of Biological and Chemical Sciences, University of Galway, University Rd., H91 TK33 Galway, Ireland; ‡ Department of Chemistry, Johannes Gutenberg University Mainz, Duesbergweg 10−14, 55128 Mainz, Germany; § Institute of Immunology, University Medical Center Mainz, Langenbeckstr.1, 55131 Mainz, Germany; ∥ Department of Chemistry, Umeå University, KBC-Building, Linneaus väg 6, S-907 36 Umeå, Sweden; ⊥ Department of Molecular Cell Biology and Immunology, Amsterdam UMC Location Vrije Universiteit Amsterdam, De Boelelaan 1117, 1081 HV Amsterdam, the Netherlands; # Amsterdam Institute for Infection and Immunity, Cancer Immunology1105 AZ Amsterdam, the Netherlands; ¶ Glycosciences Laboratory, Imperial College London, W12 0NN London, U.K.; ∇ AFFINImeter Scientific & Development Team, Software 4 Science Developments, 15782 Santiago de Compostela, A Coruña, Spain; ○ Departamento de Física Aplicada, Facultad de Física, Universidad de Santiago de Compostela, 15705 Santiago de Compostela, Spain; ⧫ Division of Experimental Medicine, Department of Medicine, Beth Israel Deaconess Medical Center and Harvard Medical School, Boston, Massachusetts 02215, United States; †† SSPC, the Science Foundation Ireland Research Centre for Pharmaceuticals, University of Galway, University Rd., H91 TK33 Galway, Ireland

## Abstract

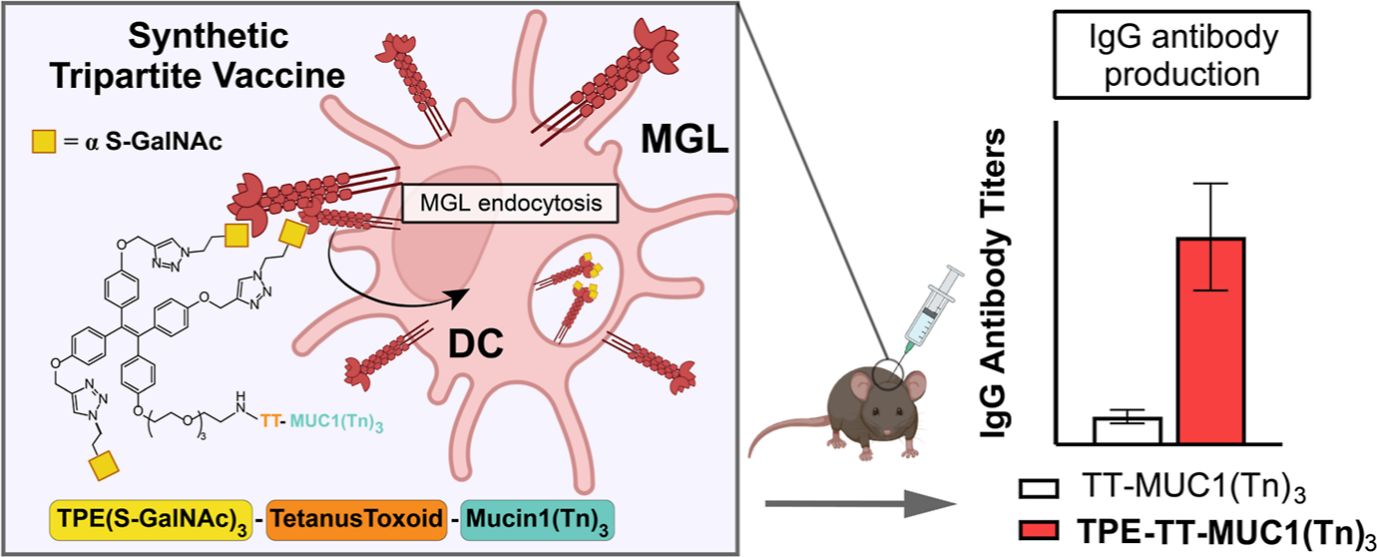

Mucin expression and glycosylation patterns on cancer cells differ markedly from healthy cells. Mucin 1 (MUC1) is overexpressed in several solid tumors and presents high levels of aberrant, truncated O-glycans (e.g., Tn antigen). Dendritic cells (DCs) express lectins that bind to these tumor-associated carbohydrate antigens (TACAs) to modulate immune responses. Selectively targeting these receptors with synthetic TACAs is a promising strategy to develop anticancer vaccines and to overcome TACA tolerance. In this work, we prepared, via a solid phase peptide synthesis approach, a modular tripartite vaccine candidate, incorporating a high-affinity glycocluster based on a tetraphenylethylene scaffold, to target the macrophage galactose-type lectin (MGL) on antigen presenting cells. MGL is a C-type lectin receptor that binds Tn antigens and can route them to human leukocyte antigen class II or I, making it an attractive target for anticancer vaccines. Conjugation of the glycocluster to a library of MUC1 glycopeptides bearing the Tn antigen is shown to promote uptake and recognition of the TACA by DCs via MGL. In vivo testing revealed that immunization with the newly designed vaccine construct bearing the GalNAc glycocluster induced a higher titer of anti-Tn-MUC1 antibodies compared to the TACAs alone. Additionally, the antibodies obtained bind a library of tumor-associated saccharide structures on MUC1 and MUC1-positive breast cancer cells. Conjugation of a high-affinity ligand for MGL to tumor-associated MUC1 glycopeptide antigens has a synergistic impact on antibody production.

## Introduction

The macrophage galactose-type lectin (MGL) is an endocytic receptor located on the surface of antigen-presenting cells (APCs), in particular dendritic cells (DCs) and macrophages. MGL binds to terminal α- or β-GalNAc residues in a calcium-dependent manner^1−3^ and can employ a secondary binding site outside the carbohydrate recognition domain (CRD) to engage with glycoproteins^4^ or disaccharides.^5^ MGL was reported to recognize Tn-modified Mucin 1 (Tn-MUC1) tandem repeat (TR) glycopeptides^6,7^ on several cancer cell lines, including colon, lung, and bronchioalveolar carcinoma.^8^ The MUC1 glycoprotein is a membrane-bound mucin found on epithelial cells, but aberrantly expressed with truncated glycans in several cancer cell lines.^9−12^

These unique MUC1 glycopeptide epitopes on epithelial cancer cells are interesting targets for immunotherapy.^13,14^ However, MUC1 immunotolerance is a major hurdle in the development of efficient anticancer vaccines.^15^ The unglycosylated protein backbone is not sufficient to trigger an immune response and unconjugated MUC1 glycopeptides are weakly immunogenic.

The role of MGL in immune regulation is not fully understood but appears to be highly dependent on the antigen structure. In some cases, glycotope interactions with MGL seem to facilitate immune escape of the pathogen via DC secretion of anti-inflammatory cytokines,^16−19^ such as interleukin-10 (IL-10),^20^ which reduces the glycolytic activity of DCs^5^ or abrogates the T helper 1/2 (Th1/2) type response in favor of regulatory T cells (Treg) or T helper 17 (Th17) type responses.^21^

However, in other cases, the Tn–MGL interaction has been leveraged to prime the immune system against tumor antigens by promoting antigen processing and presentation on APCs.

A glycopeptide therapeutic vaccine candidate based on multiple antigenic glycopeptide (MAG) composed of tri Tn glycotope, MAG-Tn3, has been shown to engage MGL to induce tumor-specific cytotoxic antibodies in breast cancer patients.^22−24^ In another study, a short peptide lacking glycosylation was bound to the MGL CRD in a calcium-dependent manner and was shown to improve survival in an ovarian cancer murine model by triggering IFN-γ release and maturation of immune cells.^25^ Furthermore, MGL expressed specifically on CD1c^+^ DCs was reported to bind and internalize Tn-MUC1 glycopeptides and enhance toll-like receptor 7/8-induced cytokine secretion.^26^

The dichotomous signaling and consequent immune response observed upon MGL engagement requires further investigation, but the remarkable example offered by Leclerc team with MAG-Tn3, and the solid evidence that MGL enhances antigen uptake and routing of tumor-associated carbohydrate antigens (TACA’s) to human leukocyte antigen class I and II compartments^27,28^ suggest that MGL targeting for prophylactic and therapeutic vaccine development should not be disregarded.

Herein, we propose to further enhance MGL-induced Tn-MUC1 uptake by DCs via a modular vaccine design strategy and incorporate a synthetic glycocluster into vaccine constructs with an aim to overcome the natural immunotolerance of MUC1.

We report the synthesis of a trivalent ligand **1**, which can be used in Fmoc solid-phase peptide synthesis (Fmoc-SPPS). This synthetic glycocluster has three α-S-GalNAc residues grafted to a tetraphenylethylene (TPE) scaffold containing an amino acid linker (TPEaa, **1**). Specifically, we generated a glycopeptide library based on the MUC1 TR HGVTSAPDT*RPAPGS*T*AP (* represents a possible glycosylation site) presenting the Tn-antigen at the possible glycosylation sites and the TPEaa **1** at the peptide’s N-terminus. We assessed conjugate binding to recombinant MGL^2^ via enzyme linked immunosorbent assay (ELISA) and found that the TPEaa-functionalized MUC1 enhanced binding to MGL. The presence of the TPEaa glycocluster **1** increased the peptide avidity for MGL approximately 10-fold (**11** vs **12** and **13**). For in vivo studies, the novel fully synthetic tripartite vaccine candidate **16**, consisting of the trivalent TPE glycocluster **1**, the P30 T helper epitope, and an antigenic Tn-modified MUC1 glycopeptide was synthesized. The tripartite construct **16**, induced a higher antibody titer compared to the control **15** that lacked the glycocluster unit **1**. The antibodies produced upon immunization with **16** bound to MUC1-Tn-positive T47D human breast cancer cells. Furthermore, the antibody selectivity was assessed via glycopeptide microarray binding assays, which revealed that the presence of glycocluster **1** did not alter the recognition pattern of the antibodies.

Both sera showed a preferential binding for the PDTR region over glycopeptides glycosylated at the GVTS or GSTA regions while binding for extensively glycosylated glycopeptides, such as core 2 and core 4 common for healthy cells mucins, was not observed.

## Results and Discussion

### Glycan and Multivalent Ligand Design

**Scheme 1:**
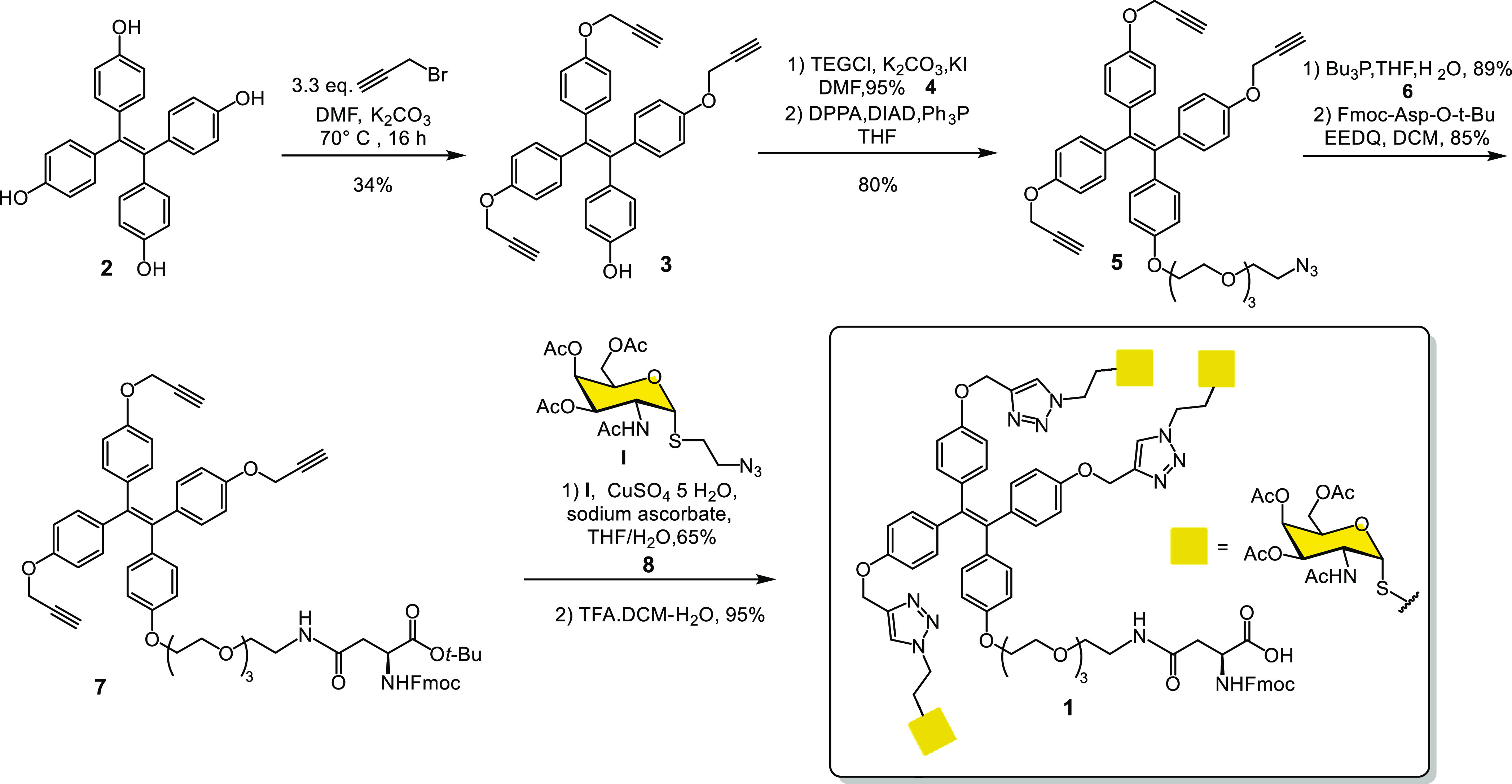
Synthesis of the Trivalent Ligand 1 to Engage MGL

MGL CRD preferentially binds GalNAc over galactopyranoside mostly due to direct hydrogen bond between the carbonyl of the acetamide group and Nε2 of the His286 imidazole group and CH−π interaction between the methyl group of the acetamide with the aromatic side chain of Tyr236.^29^ It shows a slight preference for α-Gal over the corresponding β-anomer.^2,30^ Furthermore, monovalent α-S-GalNAc had a 50-fold higher inhibitory potency than GalNAc for MGL CRD bound to crypt-associated cells of murine jejunum.^31^ Thioglycosides (S-glycosides) are accepted by most biological systems and are less susceptible to acid or enzymatic cleavage than O-glycosides. Given its enhanced affinity and well-established synthetic access, we designed a multivalent glycocluster display of the α-S-GalNAc derivative **I** (Scheme 1).

We previously assessed the ability of tetravalent and trivalent α-S-GalNAc glycoclusters in inhibiting the binding of MGL to fixed murine jejunum. The ligands containing the TPE scaffold outperformed other scaffolds with the same valency and similar geometry.^31^ This might be due to the established ability of TPE to aggregate in aqueous media^32,33^ with the increased valency of the supramolecular structure leading to higher-affinity interactions with MGL. Before embarking on the design of a synthetic route for a conjugatable glycocluster based on the TPE core, we evaluated the ability of the glycocluster **1S** (Figure S2) to bind human MGL (hMGL) by isothermal titration calorimetry (ITC). We expressed recombinantly and purified the hMGL CRD, amino acids 181–277. To mimic closely the oligomerization state of the protein on APCs, also the extracellular portion of the lectin, amino acids 81–309, containing a coiled-coil motive, amino acids 85–176,^34^ that induces the trimerization of the CRD was recombinantly expressed and purified (Figure S1).

ITC experiments (Figures S3–S5 and Table S1) revealed a high affinity of the TPE ligand for MGL, both the extracellular portion and CRD. **1S** has a *K*_d_ of 67 and 250 nM for MGL CRD and full length, respectively. The fraction of active ligand (rA) was found to be <1 (Figure S6), suggesting that the formation of supramolecular clusters blocks access to a proportion of the ligand binding sites. Furthermore, the trivalent glycocluster **1S** provides an unutilized phenol that can be harnessed to install a conjugation handle.

### Synthesis of Trivalent MGL Ligand **1**

The synthesis of **1** is summarized in Scheme 1.

The synthesis commenced from **2**, which was prepared as described previously.^35^ The direct tri-*O*-propargylation of **2** with propargyl bromide gave **3** in 34% yield. The propargylation gave also mono, di and tetra-*O*-propargylated products, however, this was still more efficient than a sequence involving the mono protection of an hydroxyl group of **2**, followed by tripropargylation and then removal of the protecting group.^31^ The phenolic compound **3** was then reacted with triethylene glycol (TEG) chloride, followed by treatment of the primary alcohol under Mitsunobu type reaction to obtain the azide **5**, which was then reduced to the corresponding amine using tributylphosphine and was subsequently coupled with *N*-Fmoc-Asp-O*t*Bu using EEDQ to give compound **7** in 85% yield. The S-GalNAc derivative **I** was synthesized as reported previously^30^ and reacted with **7** via copper(I)-catalyzed 1,3-dipolar azide–alkyne cycloaddition.^31^ The final acidic cleavage of the *tert*-butyl ester of **8** gave the desired compound TPEaa **1** for Fmoc-SPPS with an overall yield of 14%.

### Synthesis of MUC1 Peptides, Conjugates with Trivalent S-GalNAc, and Vaccine Constructs to Target MGL

Since MUC1 is ubiquitously found on epithelial cell surfaces and tumor-associated glycoforms with an aberrant glycosylation pattern are overexpressed in many carcinomas, it is an attractive antigenic target for the development of cancer vaccines. Because the immune system has a natural tolerance for endogenous structures, MUC1-based vaccines employing the unmodified MUC1 TR sequence HGVT*S*APDT*RPAPGS*T*APPA (* represents possible glycosylation sites) as the antigen are only weakly immunogenic.

To enhance the immunogenicity, TACAs can be introduced into the MUC1 vaccine. A study on monoclonal antibodies suggested that the PDTR region is immunodominant in mice that were immunized with MUC1 vaccines.^36^ Additionally, carbohydrate clusters, with the carbohydrate on adjacent Ser and Thr residues, were found to be the preferred epitopes of monoclonal antibodies.^37−40^ With this in mind, we chose the MUC1 TR sequence glycosylated with the Tn-antigen in the PDT*R and the GS*T*A regions as antigen peptides. Although mucin-derived peptides can engage and crosslink B cell receptors, assigning them the unique or sole role as B cell epitopes is limiting as they may also contain T cell epitopes. For instance, the antigenic-dominant domain of MUC1 TR, which is included in the sequence SAPDT*RPAP, can complex with MHC class I (Kb) molecules and trigger the activation of cytotoxic T lymphocytes.^41^ Furthermore, peptides can function as a shuttle to present carbohydrate antigens to T cells and generate carbohydrate-specific T cells that trigger a superior cellular immune response.^42^ On the other hand, specific T follicular helper cells promote B cell class switching and memory formation.^43^

We planned the synthesis of two sets of peptides: the first set for in vitro and in cellulo testing and the second set designed for in vivo testing.

**Figure 1:**
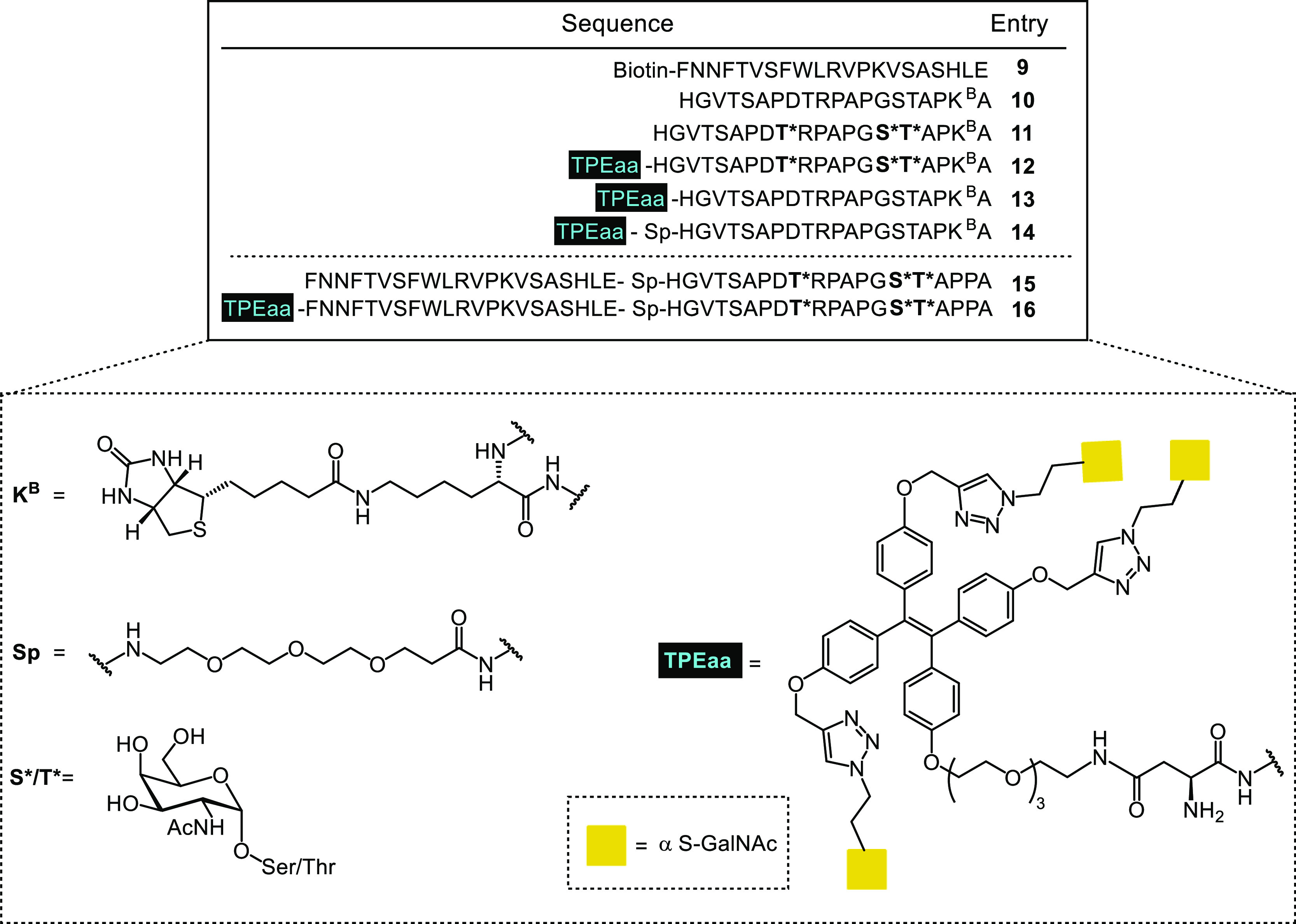
Summary of peptides **9–16** synthesized by SPPS.

For the first set, a small library of control peptides and tumor-associated MUC1 TR glycopeptides carrying Tn-antigens were synthesized (Figure 1, peptides **9–14**). One of the amino acids at the C-terminus was exchanged with a biotinylated lysine (K^B^) for peptide immobilization on streptavidin-coated ELISA plates and for fluorophore conjugation in flowcytometry and microscopy experiments. To test the influence of the TPEaa on MGL binding, ligand **1** was introduced to the N-terminus of peptides **12–14**.

In the second peptides set (Figure 1, peptides **15** and **16**), we introduced the T helper epitope P30 (FNNFTVSFWLRVPKVSASHLE), derived from tetanus toxoid, to improve the presentation of exogenous glycopeptides on the MHC complex and activate T cells for in vivo testing.^44^

The tripartite vaccine **16** contains the Tn-modified MUC1 glycopeptide, the T helper epitope P30, and the high-affinity ligand **1** to enhance endocytosis by the C-type lectin MGL. Additionally, a corresponding control glycopeptide **15** lacking the MGL glycocluster ligand **1** was prepared.

Unglycosylated and Tn-modified MUC1 peptides were synthesized by automated SPPS according to a previously described protocol for glycopeptide synthesis.^45,46^ Briefly, preloaded TentaGel R Trt Fmoc-Ala was used, and the standard Fmoc-protected amino acids as well as the biotinylated lysine were coupled using HBTU and HOBt. Glycosylated Ser and Thr amino acids (1.5 equiv) were pre-activated using the more reactive HATU and HOAt, manually added to the resin, and the coupling time was extended to 8 h. After complete assembly of peptides **14–16**, a triethylene glycol spacer (Sp) was introduced to the N-terminus. The peptides were then further elongated with the P30 epitope by Fmoc-SPPS. We inserted TPEaa **1** by a final coupling step at the N-terminus and placed this furthest away from the resin, to maximize the coupling yield. To this end, TPEaa **1** (2 equiv) was manually pre-activated with HATU/HOAt and coupled to peptides **12–14** and **16** for 8 h. The obtained peptides were cleaved from the resin with simultaneous removal of acid-sensitive protecting groups on the amino acid side chains using TFA and TIPS. The *O*-acetyl protecting groups on the glycans were removed by transesterification in methanol using catalytic amounts of sodium methoxide, and compounds **9–16** were finally isolated after purification by C-18 preparative HPLC.

### Recombinant MGL Receptor Binds to S-GalNAc MUC1 Glycopeptide Conjugates

To assess the ability of recombinant MGL to bind the synthesized peptides **9–14**, we performed an ELISA using streptavidin-coated plates to which the biotinylated peptides **9–14** were immobilized. As reported in other affinity assessment assays, we varied the peptide concentration, while the lectin concentration was kept constant.^47,48^

To determine the optimal peptides and protein range to use in the assay, we performed several pilot titrations using a biotinylated polyacrylamide coupled-GalNAc polymer (PAA-GalNAC) (Figure S7).

The biotinylated peptides were coupled to a streptavidin-coated plate at twofold serial dilutions, which was then probed with recombinant MGL at a constant concentration tagged with a human IgG1Fc fragment and goat anti-human Fc-peroxidase was used for colorimetric detection.

**Figure 2:**
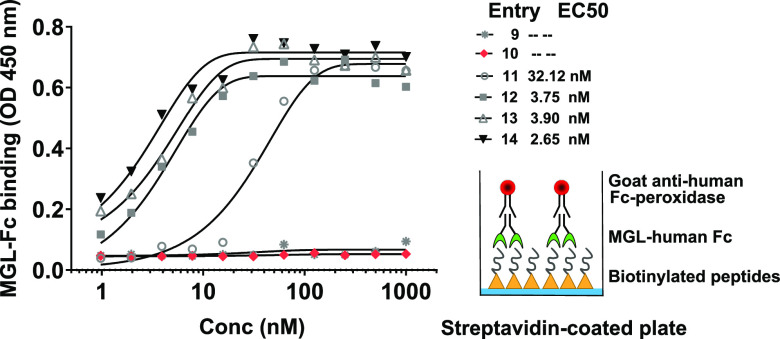
EC_50_ values for the Tn-MUC1 (glyco)peptides and their glycocluster-conjugates with MGL were obtained using ELISA assays of a dilution series of the immobilized peptides **9–14** and recombinant MGL: the biotinylated peptides **9–14** were coupled to a streptavidin-coated plate at twofold dilutions, which was then titrated with recombinant MGL-Fc. MGL binding was detected using peroxidase conjugated to a goat anti-human Fc Ab. Curve fitting is shown in the figure. For all the peptides, the *R*^2^ value of the curve fitting was above 0.98.

The EC_50_ values derived from the ELISA binding studies with TA-MUC1 peptides are shown in Figure 2. Peptides **12–14** bearing the TPE α-S-GalNAc-glycocluster showed higher avidities than peptide **11** (Figure 2). MGL exhibited similar affinities for peptides **12–14**, suggesting that MGL binds preferentially to the trivalent glycocluster over the Tn antigens on the MUC1 glycopeptide. A slight improvement in the EC50, from 3.90 nM for **13** to 2.65 nM for **14**, was observed with the addition of a spacer separating the MUC1 peptide from the α-S-GalNAc-glycocluster. MGL binding to the unglycosylated MUC1 peptide **10** or to the tetanus toxoid P30 epitope **9** was not observed, confirming that glycosylation on the MUC1 backbone or the TPEaa ligand are crucial for MGL binding.

### MGL Binds Vaccine Constructs and Mediates Antigen Uptake in Murine Bone Marrow-Derived Dendritic Cells

**Figure 3:**
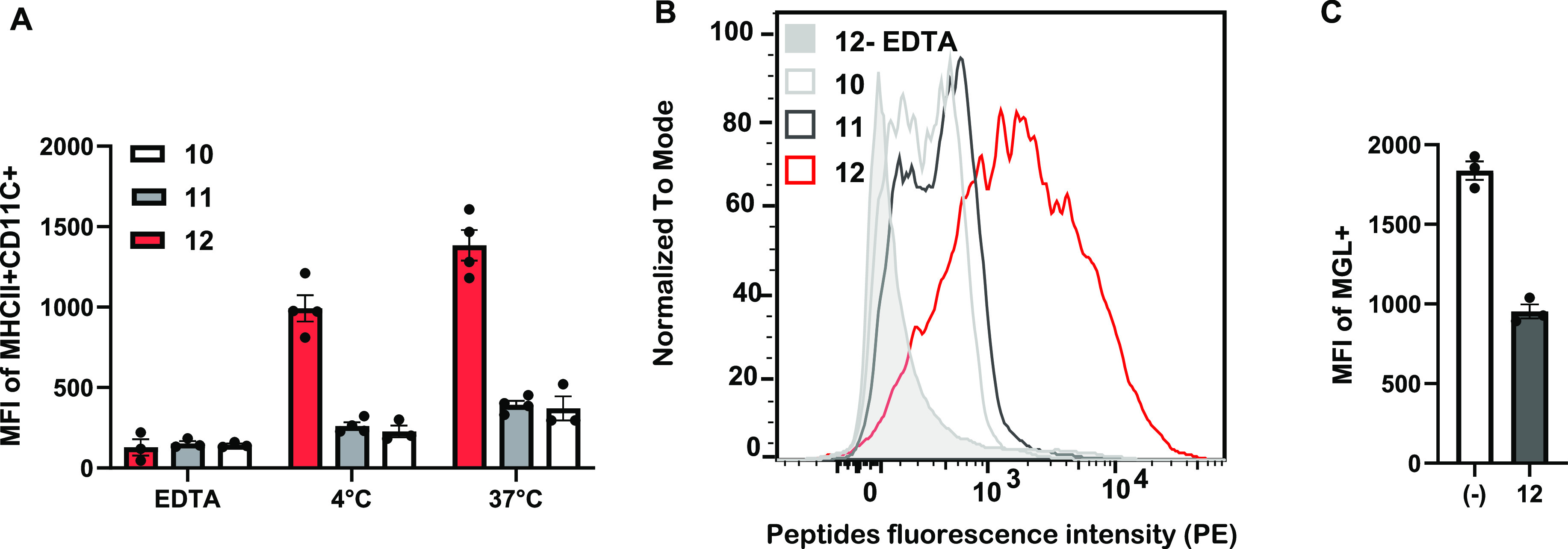
Flow cytometry analysis on BMDCs. (A) MFI of MHCII^+^CD11c^+^ cells relative to the peptides. BMDCs where incubated in the presence of the biotinylated peptides **10**, **11**, and **12** at 4 °C and then labeled with streptavidine-PE. (B) Peptides-associated fluorescence intensity histogram after incubation with BMDCs at 37 °C. (C) MFI relative to the MGL receptor on BMDC surface. BMDCs were treated with peptide **12** for 30 min at 37 °C. Error bars represent standard errors.

To assess the vaccine construct uptake by DCs via MGL, we isolated and cultured murine bone marrow-derived dendritic cells (BMDCs) and assessed the peptide uptake by flow cytometry. We allowed the peptide to bind to the cell surface at 4 °C and then labeled the constructs with streptavidin-PE. The cells were then incubated at 37 °C for 1 h to facilitate peptide internalization. Analysis of the mean fluorescence intensity (MFI) showed that the uptake of peptide **12** was fourfold larger compared to the uptake of peptides **10** and **11**. The weak fluorescence increase of **12** in the 37 °C samples compared to the 4 °C samples suggests a receptor-mediated uptake mechanism (Figure 3A,B).

To verify whether the uptake was C-type lectin dependent, we sequestered calcium ions with EDTA. In the presence of the chelating agent, the binding was significantly diminished (Figure 3A,B). Furthermore, we used anti-MGL antibodies to evaluate the presence of MGL on the surface of the BMDCs post incubation with peptide **12**. The amount of accessible MGL on the cell surface decreased by 48% in the samples treated with peptide **12** compared to the untreated cells (Figure 3C).

**Figure 4:**
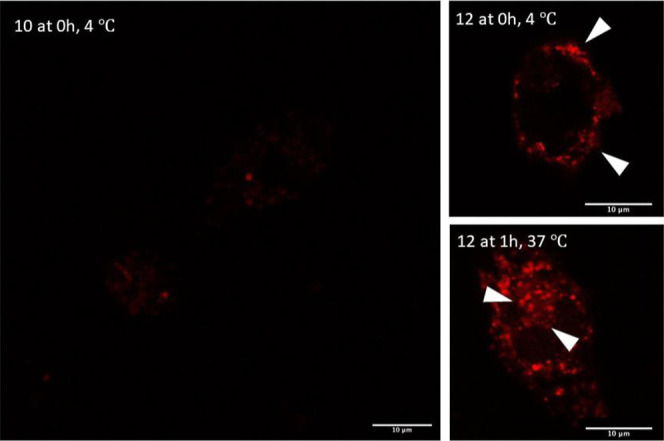
Comparison of fluorophore-labeled MUC1 peptides **10** and **12** by microscopy. BMDCs were treated with 5 μg/mL of the biotinylated peptides **10** and **12** at 4 °C for 1 h followed by washing and treatment with streptavidin-Alexa 594. (A,B) Localization of the peptides on the cells surface. (C) Localization of the peptides after incubation for 30 min and 1 h at 37 °C. Internalization was visualized by confocal microscopy.

Peptide uptake was investigated by confocal microscopy. Peptide **10**, serving as a negative control, and peptide **12** were incubated at 4 °C with BMDCs to allow binding to the MGL receptor. After a washing step, we labeled the bound peptides with streptavidin-Alexa 594. The cells incubated with the unglycosylated peptide **10** showed only unspecific binding (Figure 4A), while **12** showed high levels of cell surface binding (Figures 4B,C and S8). After incubation of the cells for 1 h at 37 °C, uptake of peptide **12** into the cells was detected. No uptake of **12** was observed at 4 °C, suggesting that the uptake is predominantly receptor mediated, thus excluding non-specific mechanisms of internalization. Again, no binding or uptake of peptide **10** was observed under any conditions, corroborating the observations and conclusions from flow cytometry experiments.

### S-GalNAc Glycocluster-MUC1 Conjugate Vaccine 16 Induces Higher IgG Antibody Titers

**Figure 5:**
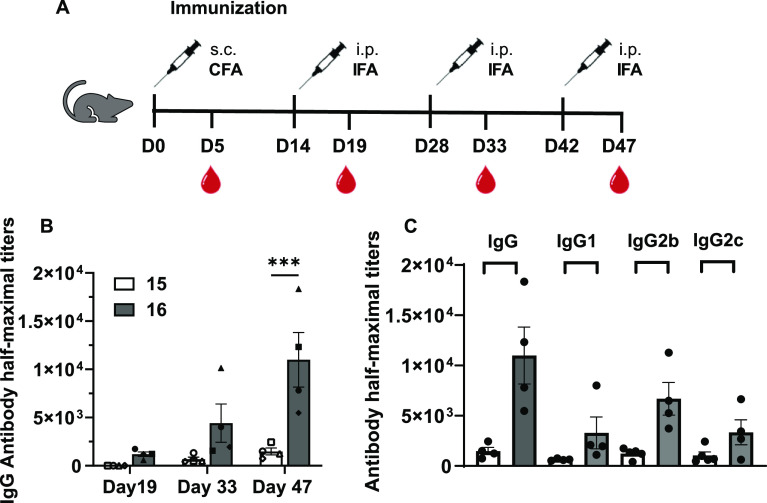
In vivo experiment immunization schedule and antibody analysis. (A) Experimental design: 2.5 nmol of construct per injection; day 0, immunization subcutaneous (s.c.) with complete Freund’s adjuvant (CFA); day s14, 28, and 42, immunization intraperitoneal (i.p.) with incomplete Freund’s Adjuvant (IFA); blood collection: 5 days post immunization. (B) IgG titers in the serum of mice immunized with constructs **15** and **16**, respectively. The sera were collected at days 19, 33, and 47. (C) Total IgG and IgG subtypes from sera collected at day 47 from mice immunized with constructs **15** and **16**. All ELISA measurements were performed on plates coated with BSA-(**11**)_4-17_ synthesized as reported in the Supporting Information. The *p* values are determined through two-way ANOVA with Šídák multiple comparison test using GraphPad Prism, ***p < 0.001; error bars indicate standard error.

With the MUC1-p30 vaccine conjugates **15** and **16** in hand, we tested their ability to induce a strong and specific immune response in mice, in order to evaluate whether the trivalent MGL ligand S-GalNAc on the TPEaa glycocluster **16** would be beneficial compared to the non-functionalized derivative **15**. Groups of four female C57BL/6J mice were immunized with conjugates **15** and **16** four times in 2-week intervals (days 0, 14, 28, and 42) with equal amounts of MUC1 peptides per injection. Serum samples were collected 5 days post-immunization, and antibody titers were determined by ELISA (Figure 5A). Glycopeptide **11** was conjugated to BSA, immobilized on ELISA plates, and probed with the antibody sera at twofold dilutions. The TPEaa vaccine **16** induced higher IgG antibody titers than the vaccine construct **15**. At day 47, the IgG concentration in the sera of mice immunized with **16** is over sevenfold higher than that observed in mice immunized with **15** (Figure 5B). IgG subtyping analysis showed that vaccine **15** elicited IgG1, IgG2b, and IgG2c in a ratio 0.20:0.40:0.40, while vaccine **16** elicited IgG21, IgG2b, and IgG2c in a ratio 0.24:0.50:0.26 (Figure 5C). The antibody subtyping profile of 16, specifically the similar IgG1/IgG2c levels and higher IgG2b levels, suggest a mixed Th1/Th2 response. Previous work showed that the antibody subclasses profile of Qβ virus-like particles conjugated with 270 copies of STnMUC1(SAPDT*RPAP)^49,50^ predominantly yielded a Th1-type profile. Construct **16** could be used in complementary settings to Qβ-(STnMUC1)_270_ when a mixed Th1/Th2 response is desired.^51^ The produced IgG2b antibodies may activate antibody-dependent cell-mediated cytotoxicity and complement-dependent cytotoxicity, and future studies will investigate further avenues and therapeutic potential in detail.

### Antibodies Induced by Glycocluster-MUC1 Conjugate Vaccine Bind to Native Breast Cancer Cells

**Figure 6:**
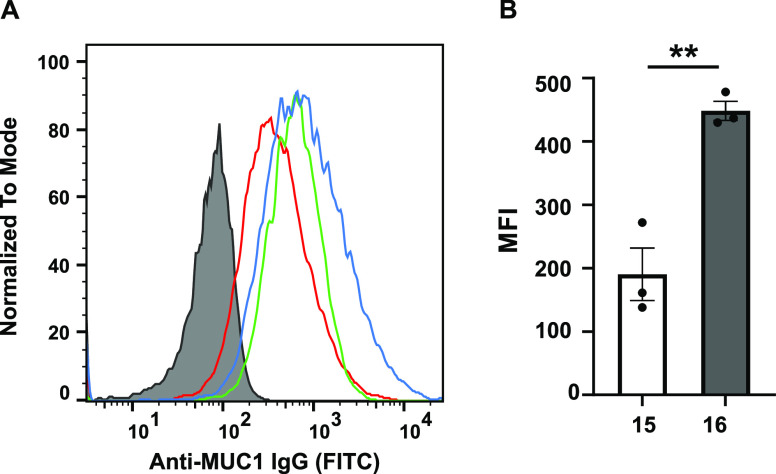
Flow cytometry analysis of the specific recognition of breast tumor cells T47D by antibodies. (A) MFIs of binding of T47D-MUC1 cells by sera (1:100) of three mice (red, green, and blue curves) immunized with construct **16**. The gray filled trace is from pre-immune serum. (B) Comparison of the MFIs of antibodies from sera of **15** and **16** (1:100). Error bars represent ±1 standard error from the mean of three biological replicates (*n* = 3). MUC1 expression on TD47 was confirmed by anti-MUC1 mAb HPMV (1:5 dilution) produced in-house.^53^ The *p*-values were determined through a two-tailed *t*-test using GraphPad Prism, **p < 0.01; error bars indicate standard error.

Synthetic glycopeptide vaccines often employ glycans with lower degrees of complexity compared to native tumor antigens. These simpler constructs are not always able to induce specific antibodies that can recognize native tumors.^14^ In order to determine the ability of the induced antibodies to recognize native cancer cells, sera collected at day 47 were incubated with T47D human breast cancer cells, which are known to overexpress aberrantly glycosylated MUC1.^52^ Bound antibodies were labeled with a fluorescent anti-IgG secondary antibody for detection by flow cytometry. The MUC1-Tn antibodies induced after immunization with **15** and **16** recognized and bound to native breast cancer cells. As observed previously with the ELISA, vaccine construct **16** elicited a higher antibody titer compared to **15** (Figure 6).

### Evaluation of Glycan Specificity of Antibodies Induced by **15** and **16**

**Figure 7:**
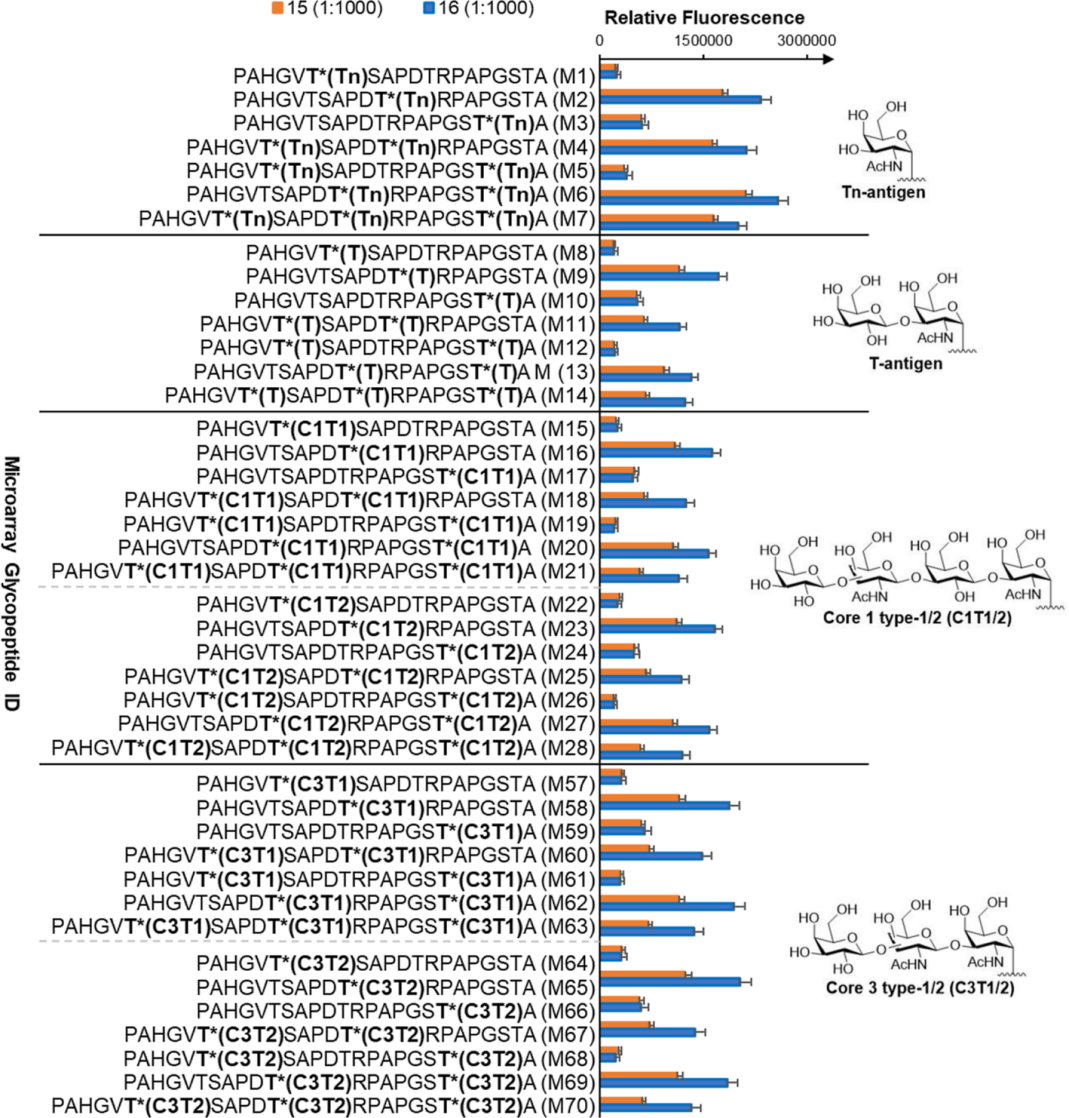
Binding specificity of antibodies generated by vaccines **15** and **16** to mucin core Tn antigen, T antigen, and **1** and **3** glycopeptides. Binding of sera at 1:1000 dilution to an array of varied mucin peptide sequences, glycosylation sites, and glycan structures are shown. Mouse sera derived from **15** and **16** showed similar recognition patterns. However, higher binding to MUC1 glycopeptides was observed for mice immunized with **16**. For complete figures, see S14 and S15. Bars represent standard error (*n* = 5).

In order to evaluate the ability of vaccine conjugates **15** and **16** to induce strong and specific immune responses in vivo, the binding specificities of the polyclonal mouse sera obtained from immunization with vaccines **15** and **16** were determined using a synthetic MUC1 glycopeptide microarray library (Table S2). The microarrays were incubated with the obtained sera at a 1:1000 dilution. Bound antibodies were detected using a secondary Cy5-labeled goat anti-mouse IgG antibody. Microarray analysis showed similar binding patterns for antibodies elicited by vaccines **15** and **16**, demonstrating that addition of the TPEaa does not affect antibody recognition (Figure 7). Additionally, higher antibody titers were again observed using construct **16**. Furthermore, the antibodies exhibited preferences for glycosylation in the immune-dominant PDTR region over peptides glycosylated in the GVTS or GSTA regions.

**Figure 8:**
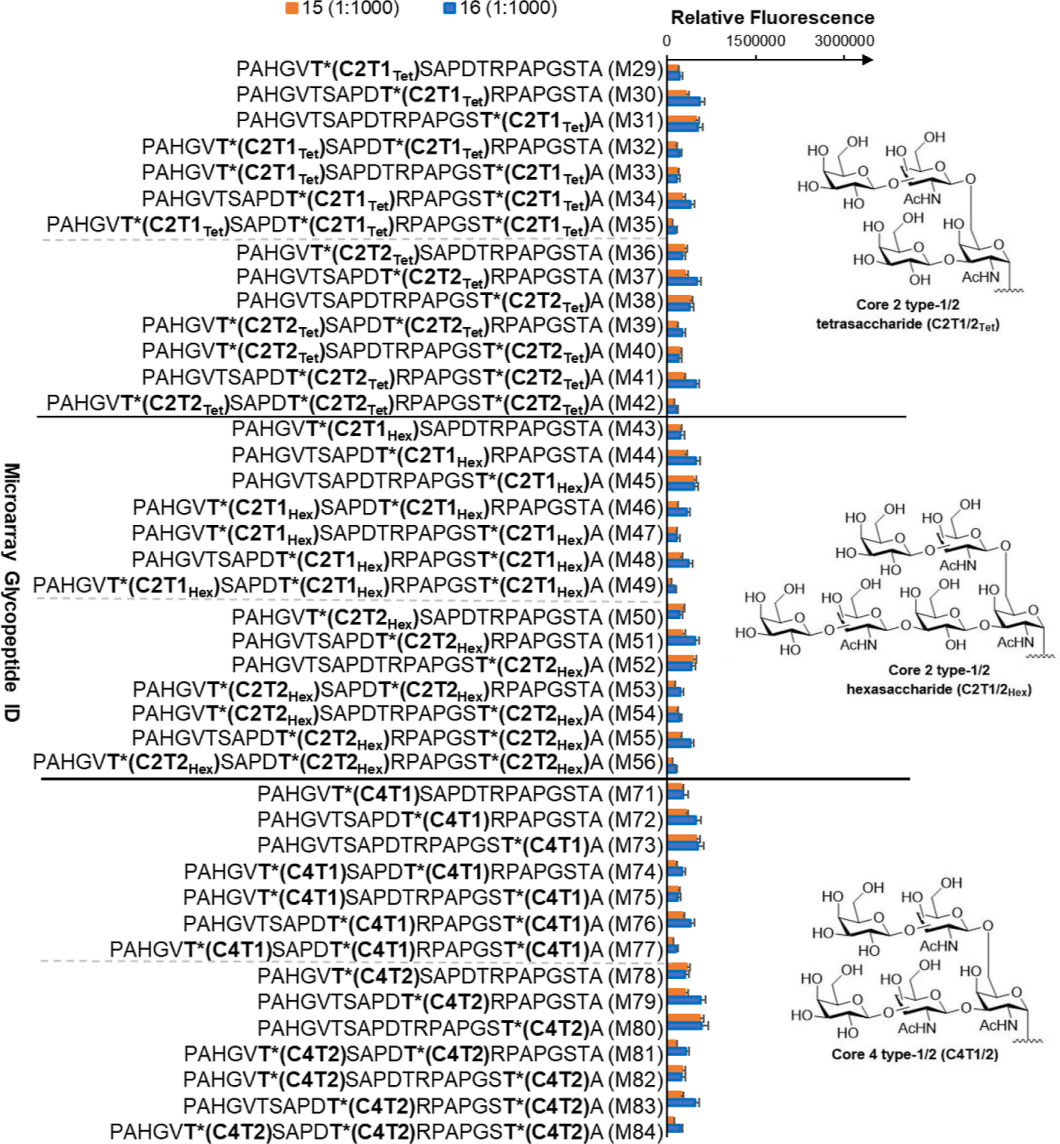
Binding specificity of antibodies generated by vaccines **15** and **16** to mucin core 2 and 4 glycopeptides. Binding of sera at 1:1000 dilution to an array of varied mucin peptide sequences, glycosylation sites, and glycan structures are shown. Mouse sera derived from **15** and **16** showed preferential binding to tumor-associated mucin core. For complete figures, see S14 and S15. Bars represent standard error (*n* = 5).

Tn antigens and shorter linear mucin-type *O*-glycan core structures, typically found on cancer cells in patients with poor prognosis,^54,55^ were better recognized by all sera compared to the peptides with extensive glycosylation with branched core 2 and core 4 structures, typically found on healthy cells. These latter diminished antibody recognitions of all antisera (Figure 8), suggesting that vaccine conjugates preferentially induced a tumor-specific humoral immune response.

Variations in selectivity among the sera from mice individuals for Tn antigens and other more extended glycan structures, as well as preferences for peptide epitopes glycosylated in the GSTA region, were observed. Importantly, these variations did not depend on the respective vaccine construct but could be attributed to the natural antibody production of the respective mouse.

No significant cross-reactivity with MUC4 and MUC5B glycopeptides carrying T- and Tn-antigens could be observed (Figures S16 and S17), indicating that the induced antibodies were specific for tumor-associated MUC1 glycopeptides rather than the glycan epitope only.

## Conclusions

We leveraged the increased affinity of TPE glycocluster constructs for MGL to develop a novel anti-cancer vaccine. We established a synthetic route to generate a synthetic tripartite vaccine containing a trivalent TPE glycocluster based on S-GalNAc to enhance MGL binding, a P30 T helper epitope and an antigenic Tn-modified MUC1 glycopeptide to induce tumor-specific antibodies in mice. ELISA experiments with recombinant MGL showed that MUC1-Tn glycopeptides displaying the trivalent TPE glycocluster were stronger binders compared to the corresponding MUC1-Tn glycopeptides without the trivalent S-GalNAc ligand. Additionally, flow cytometry analysis showed that the glycocluster facilitated increased internalization by DCs via the MGL receptor compared to the glycopeptide alone. The highest MGL binder was then introduced into the tripartite vaccine for immunization, leading to high titers of anti-Tn-MUC1 antibodies in vivo. The obtained antibodies were able to recognize MUC1-positive breast cancer cells. Microarray analysis showed that immunization with the vaccine construct bearing the multivalent S-GalNAc glycocluster led to increased antibody production without causing loss of selectivity for the target antigen. Furthermore, the antibodies exhibited preferences for short and linear mucin *O*-glycan core structures in the immune-dominant PDTR MUC1 region. Our results indicate that the vaccine construct **16** efficiently targets MGL, thus increasing antigen endocytosis by the MGL receptor and consequently eliciting a strong humoral response.

Thus, we created a structurally defined tripartite vaccine to enhance endocytosis and evaluated it in vitro and in vivo. The reported construct is based on a modular design and synthesis approach and offers the potential to transfer this modularity concept more widely to vaccine design. For example, different glycocluster ligands of high affinity for other DC lectins could be incorporated to investigate their properties,^56^ or other glycopeptide constructs more efficiently targeted to DCs via MGL using the TPE ligand. Overall, our findings prove, for the first time, that the concept of targeting clinically relevant vaccine epitopes with a rationally designed MGL-ligand is applicable successfully in vivo, resulting in a boosted immune response.

## Materials and Method

### Mouse Immunization

8 weeks old C57BL/6J female mice (Charles River) were housed and maintained in microisolator cages under specific pathogen-free conditions at the animal facility of Johannes Gutenberg-University according to institutionally approved protocols (permission was obtained from the Landesuntersuchungsamt Koblenz, 23 177-07/G 19-1-099). The mice received the first immunization s.c. with CFA on day 0 in a total volume of 50 μL into the right flank. The mice were immunized i.p. on days 14, 28, and 42 with IFA in a volume of 100 μL with a dose of 2.5 nmol per injection. Blood was collected from the tail vein five days post each vaccination, and serum was prepared by centrifugation for 15 min at 2500*g*.

### Anti-Muc1 Antibody Quantification and Subtyping

ELISA was used for the analysis of induced anti-Muc1 antibodies in antiserum. 96-well MaxiSorp plates were coated by incubating overnight at 4 °C with 1 μg/mL glycopeptide 11 in BSA, washed three times with washing buffer (PBS containing 0.05% Tween-20), and blocked for 30 min at 37 °C with blocking buffer (washing buffer supplemented with 1% BSA). Dilutions of sera in blocking buffer were added and incubated for 2 h at 37 °C. The plates were washed three times with washing buffer and incubated with the different antibody subtypes IgG1, IgG2b (BD Pharmigen), IgG2a (PharMingen), IgG2c (Abcam), and IgG3 (BioLegend). After incubation and three washes, horseradish peroxidase-conjugated goat anti-mouse IgG (H + L) (Abcam) diluted 1:1000 in blocking buffer was added for 1 h at 37 °C. The plates were then washed three times and incubated for 10 min with 50 μL per well of 2,2′-azino-di-(3-ethylbenzothiazoline sulfonic acid substrate in citrate buffer (pH 4.4 and 1:4000) 30% H_2_O_2_. The reaction was quenched by adding 50 μL of 1 M aqueous sulfuric acid to each well. Plates were read at 410 nm using a Tecan Infinite F200 Pro microplate reader. For each IgG of the antiserum, optical densities were measured and the absorbance curves were fitted to obtain antibody titers. There were no IgGs detectable for the negative controls (untreated mice).

### Anti-Muc1 Antibody Recognition of Breast Cancer Cells by Flow Cytometry

100.000 MUC1-positive T47D tumor cells (ATCC, Maryland USA) were incubated with 1 μL of the serum for 30 min at 4 °C in PBS. The cells were then washed two times with PBS and incubated with an Alexa Fluor 488 conjugated goat-anti-mouse-IgG secondary antibody (Invitrogen) and with fixable viability dye eFluor780 (Invitrogen) to exclude false-positive dead cells. The cells were washed two times and subsequently taken up and analyzed. Flow cytometry data were acquired on a Canto flow cytometer (BD Biosciences) and analyzed with FlowJo Software (FlowJoLLC).

## Supporting Information Available

Experimental details and characterization of the synthetic antigens (PDF)
