# Supplementary material for: MUC1 Glycopeptide Vaccine Modified with a GalNAc Glycocluster Targets the Macrophage Galactose C-type Lectin on Dendritic Cells to Elicit an Improved Humoral Response
Source: J Am Chem Soc. 2023 Jun 6;145(24):13027–37. doi: 10.1021/jacs.2c12843 (PMC10288512; doi:10.1021/jacs.2c12843)
Supplement: Supplementary file 1 — ja2c12843_si_001.pdf [file ja2c12843_si_001.pdf]

MUC1 Glycopeptide Vaccine Modified with a GalNAc Glycocluster Targets the Macrophage Galactose C-Type Lectin on Dendritic Cells to Elicit an Improved Humoral Response

Adele Gabba[1,2*], Riem Attariya[3], Sandra Behren[4], Christian Pett[4,] Joost C. van der Horst[5,6], Hajime Yurugi[3], Jin Yu[7], Moritz Urschbach[2], Juan Sabin[8,9], Gabriel Birrane[10], Edgar Schmitt[3], Sandra J. van Vliet[5,6,**], Pol Besenius[2,**], Ulrika Westerlind[4,**], Paul V. Murphy[1,11**]

[1]School of Biological and Chemical Sciences, University of Galway, University Rd., Galway H91 TK33, Ireland.

[2]Department of Chemistry Johannes Gutenberg University Mainz Duesbergweg10–14,55128 Mainz, Germany.

[3]Institute of Immunology University Medical Center Mainz Langenbeckstr.1, 55131 Mainz, Germany.

[4]Umeå University, Department of Chemistry, KBC-building, Linneaus väg 6, S-907 36 Umeå, Sweden.

[5]Amsterdam UMC location Vrije Universiteit Amsterdam, department of Molecular Cell Biology and Immunology, De Boelelaan 1117, Amsterdam, the Netherlands

[6]Amsterdam Institute for Infection and Immunity, Cancer Immunology, Amsterdam, the Netherlands

[7]Glycosciences Laboratory, Imperial College London, W12 0NN UK

[8] AFFINImeter Scientific & Development Team, Software 4 Science Developments, Santiago de Compostela, A Coruña 15782, Spain.

[9]Departamento de Física Aplicada, Facultad de Física, Universidad de Santiago de Compostela, Santiago de Compostela, Spain.

[10]Division of Experimental Medicine, Department of Medicine, Beth Israel Deaconess Medical Center and Harvard Medical School, Boston, Massachusetts 02215, United States.

[11]SSPC, the Science Foundation Ireland Research Centre for Pharmaceuticals, University of Galway, University Rd., Galway H91 TK33, Ireland.

*Corresponding author and first author

** Joint senior author

Email: adele.gabba@gmail.com

Supporting information

1 Glycocluster affinity experiments 3
1.1 Isothermal titration calorimetry: protein expression and purification 3
1.2 Isothermal titration calorimetry experiments 5
2 Synthesis general experimental conditions 11
2.1 Synthesis of TPE ligand 1 12
3 Solid phase peptide synthesis and related purification: General conditions 34
3.1 General protocol for automated glycopeptide-solid phase peptide synthesis 35
3.2 Manual coupling protocol for Glycosylated amino acids 35
3.3 Spacer manual coupling protocol 35
3.4 Biotinylated lysine manual coupling protocol 36
3.5 Manual coupling protocol 36
3.6 Release of the peptides from the solid phase resin protocol 36
3.7 Removal of the carbohydrate acetyl protecting groups protocol 37
3.8 Synthesis yields and analytical data for peptides 9 to 14 38
3.9 Synthesis yields and analytical data for peptides M96 to M98 42
4 *In vitro & in cellulo* peptide uptake study 44
4.1 Biotinylated MUC1 peptides/MGL-Fc ELISA protocol 44
4.2 Murine bone marrow derived dendritic cell generation 45
4.3 Flow cytometry uptake 45
4.4 Microscopy 46
5 Post immunization and sera analysis 46
5.1 Generation of BSA-$(11)_n$ for ELISA plate coating 46
6 Microarray fabrication and binding studies 50
6.1 General spotting conditions 50
6.2 Microarray binding studies with mouse sera 51
7 References 56

1 Glycocluster affinity experiments

1.1 Isothermal titration calorimetry: protein expression and purification

hMGL CRD, amino acids 181−277, was expressed as previously published.[1] Briefly, a gene fragment corresponding to the extracellular domain of MGL isoform 3 was purchased from IDT (Coraville, IA) and amplified by polymerase chain reaction (PCR) using appropriate primers. The amplification conditions contained a single denaturation cycle at 98 ºC for 2 min followed by 30 cycles of denaturation at 98 ºC for 10 sec, primer annealing at 60 ºC for 15 sec and template extension at 72 ºC for 30 sec, followed by a final 72 ºC extension step for 5 min. The PCR product was digested with the restriction nucleases BamH1-HF and EcoR1-HF, purified and ligated into a modified pTriEx expression vector at the BamH1-HF and EcoR1-HF sites. DH5α cells were transformed with the ligated product and the resulting colonies were screened for the presence of the insert by restriction digestion followed by agarose gel electrophoresis. The clone was verified by sanger sequencing.

hMGL extracellular portion, amino acids 81-316, was expressed and purified as briefly reported below. 100ng of plasmid DNA was used to transform C41(DE3) cells using standard procedures and colonies were grown overnight on LB agarose plates containing 100μg/ml ampicillin. A single colony was used to inoculate a 10 ml overnight culture containing 100μg/ml ampicillin at 37 ºC. 10 ml of the overnight culture was diluted into 1 L of Miller Broth containing 100 μg/ml of ampicillin and the cells were grown at 37 ºC until OD_{600} of 0.6 was reached. Protein expression was induced at 37 ºC for 4h by adding IPTG to a final concentration of 1mM. The cells were harvested by centrifugation, washed with PBS buffer and stored at -80 °C until further use. The protein was expressed as inclusion bodies and purified by a refolding procedure. 1L of cell pellet was resuspended in 25ml of PBS, lysed by sonication and the inclusion bodies were harvested by centrifugation. The inclusion bodies were further purified by cycles of resuspension, sonication and centrifugation using buffer **1** (20mM Tris pH 8.0, 0.5% Triton-100, 5 mM EDTA pH 8, 25 mM b-ME) followed by buffer **2** (20mM Tris pH 8.0, 1 % Triton-100, 5 mM EDTA pH 8, 25 mM b-ME, 500 mM NaCl) and finally buffer **3** (20mM Tris pH 8.0, 5 mM EDTA pH 8, 25 mM b-ME, 1 M NaCl). The pellet after centrifugation was solubilized by stirring in 25 mL of 2 M NH_4OH at room temperature for 1h.

The solubilized protein was slowly diluted tenfold into refolding buffer (20mM Tris pH 7.5, 500 mM NaCl, 25 mM $CaCl_2$, 1mM reduced glutathione, 0.1 mM oxidized glutathione), filtered and dialysis twice against 5L of the same buffer for a period of 8-16 hrs. The addition of 400 mM L-arginine allows refolding in 4 h. The refolded protein was filtered and purified by affinity chromatography using cOmplete His-Tag purification resin (Roche), (Figure S1). The soluble protein was incubated with the resin at 4 ºC for 5 h, washed with 20 mM Tris pH 7.5, 500 mM NaCl and 5 mM imidazole and eluted with 20 mM Tris, 150 mM NaCl and 200 mM imidazole, pH 7.5.

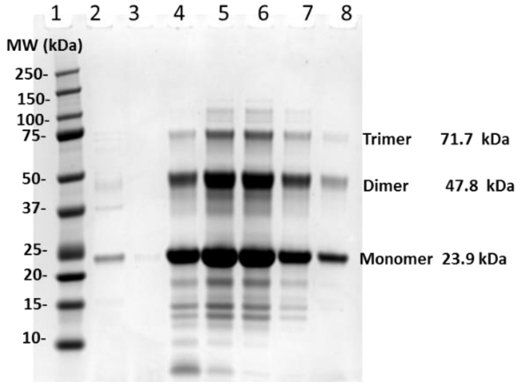

Figure S1.SDS-PAGE of Ni-NTA purification, 6His hMGL (81-309) monomer 23.9 kDa, dimer 47.8 kDa, trimer 71.7 kDa. Lane 1 Bio-rad Precision Plus Protein kaleidoscope marker (10 µL), lane 2 flow through, lane 3 wash step, lane 4 elution test tube #1, lane 5 elution test tube #2, lane 6 elution test tube #3, lane 7 elution test tube #4, lane 8 elution test tube #4.

The protein was concentrated and dialysed to reduce the salt concentration. The 6xHis tag was removed by treating the protein with TEV protease (generated in-house) at 4 ºC for 16 h.

Samples were further purified with a HiPrep QFF 16/10 anion exchange chromatography column (Cytiva) in buffer A (20 mM Tris pH 7.5, 40 mM galactose, 2 mM $CaCl_2$) and eluted with a gradient of buffer B (buffer A supplemented with 1M NaCl). Pooled samples containing MGL were concentrated 1ml and applied to a Superdex S200 size exclusion chromatography column (Cytiva) with a running buffer containing 20 mM Tris pH 7.5, 40 mM galactose, 2 mM $CaCl_2$ and 150 mM NaCl. Fractions corresponding to MGL were collected, concentrated and applied to a polyacrylamide column equilibrated with 20 mM Tris pH 7.5, 2 mM $CaCl_2$, 150 mM NaCl to remove the galactose.

1.2 Isothermal titration calorimetry experiments

Binding constants of the ligand **1S**, (Figure S2) with hMGL CRD (181-277) and hMGL extracellular portion (81-309), were measured by isothermal titration calorimetry (ITC) using a MicroCal VP-ITC MicroCalorimeter (Malvern). hMGL was dialyzed against a buffer containing 20 mM Tris pH 7.5, 2 mM $CaCl_2$, 150 mM NaCl. The lyophilised glycocluster ligands were dissolved in the same buffer. The experiments were performed at 25 °C. The sample cell was loaded with hMGL solution, and the protein was titrated with the glycocluster solution as typical for forward ITC analysis. Protein concentration was kept low (7-20 µM) to avoid precipitation and it was determined by measuring the absorbance at 280 nm after centrifuging the sample at high speed for 10 min. We used molar concentrations of multivalent carbohydrate analogues, not their epitope equivalence.

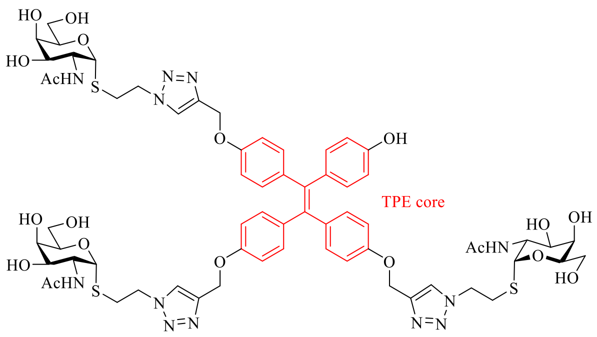

1S

Figure S2. Chemical structure of the trivalent glyclusters **S1** based on the tetraphenyl ethylene core. **S1** was synthesized as previously reported.[2]

A total of 33 automatic injections of 8 µl each were carried out with a delay of 320 seconds between each injection. The heats of dilution, determined by the titration of the sugar into the same buffer alone, were subtracted from the raw titration data before data analysis. AFFINImeter ITC[3] software (Software 4 Science Developments S.L., Spain) was used to create stoichiometric binding models considering one, three and four sequential binding events:

$M + A \leftrightarrow MA$

$M + A \leftrightarrow MA + M \leftrightarrow M_2A + M \leftrightarrow M_3A$

Where M is the monomeric or trimeric lectin in the cell and A is the monovalent or trivalent glycocluster.

The 1:1 experiment were used to determine the correction in active concentration of the protein in cell (r_m). While r_m was kept fixed for the rest of experiments, the correction in the active concentration of the ligand in syringe (r_a) was used as a fitted parameter to determine the degree of aggregation of the ligand. Figures S3 and S4 show a representative example of the analysis, while Table S1 shows the thermodynamic parameters of these analysis. k_{on} and k_{off} values were calculated using AFFINImeter (Figure S5). Figure S6 shows the rA trend with increasing ligand valency.

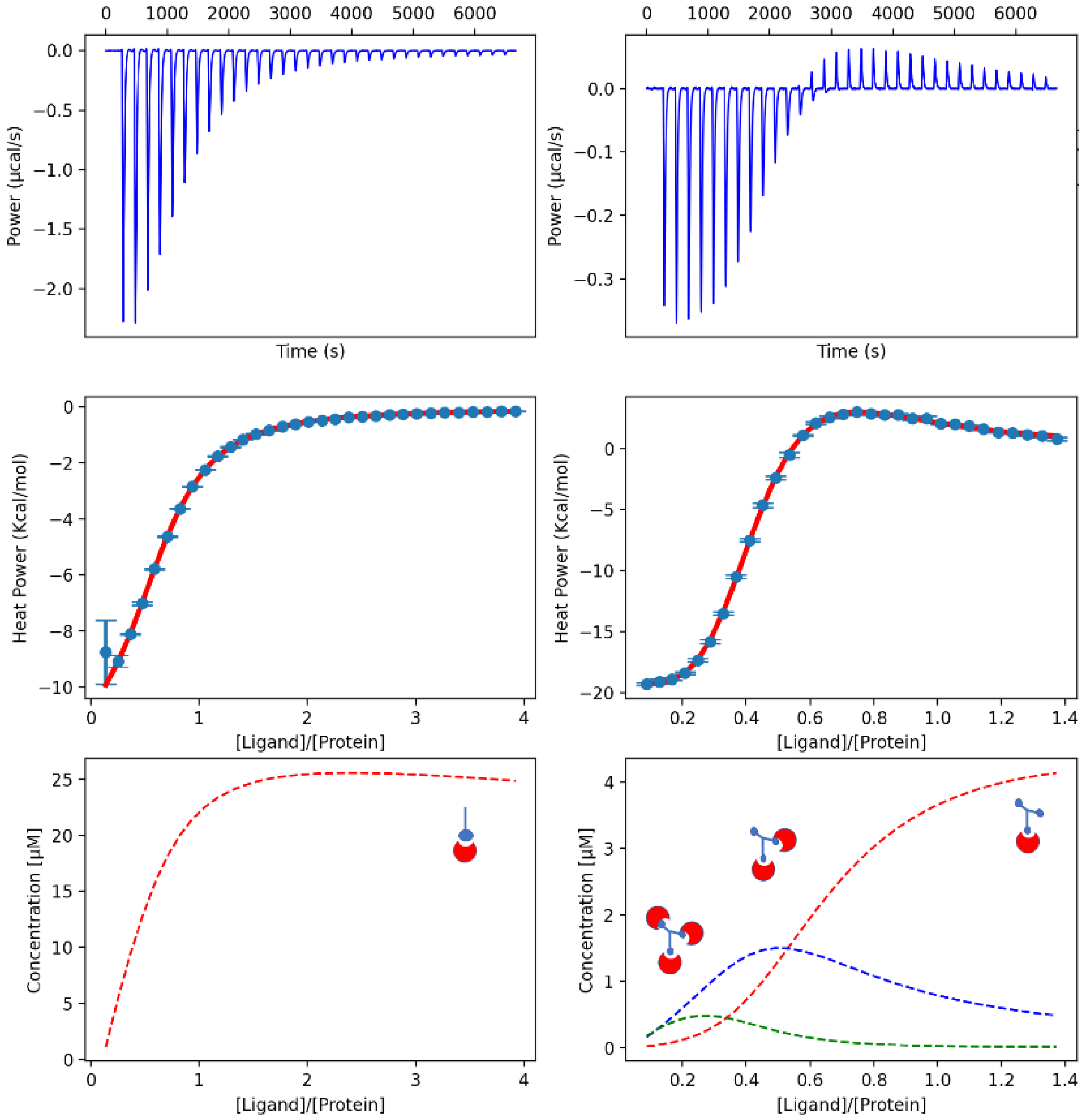

Figure S3. ITC analysis of MGL CRD (181-277) with monovalent and trivalent ligands.

Upper panels show the raw data for titration of ligands into lectins. Middle panels show the corresponding isotherms after peak integration with the analysis fittings using the binding model described above. Lower panels show the distribution of each complex during the titration (green for M_3A, blue for M_2A and red for MA)

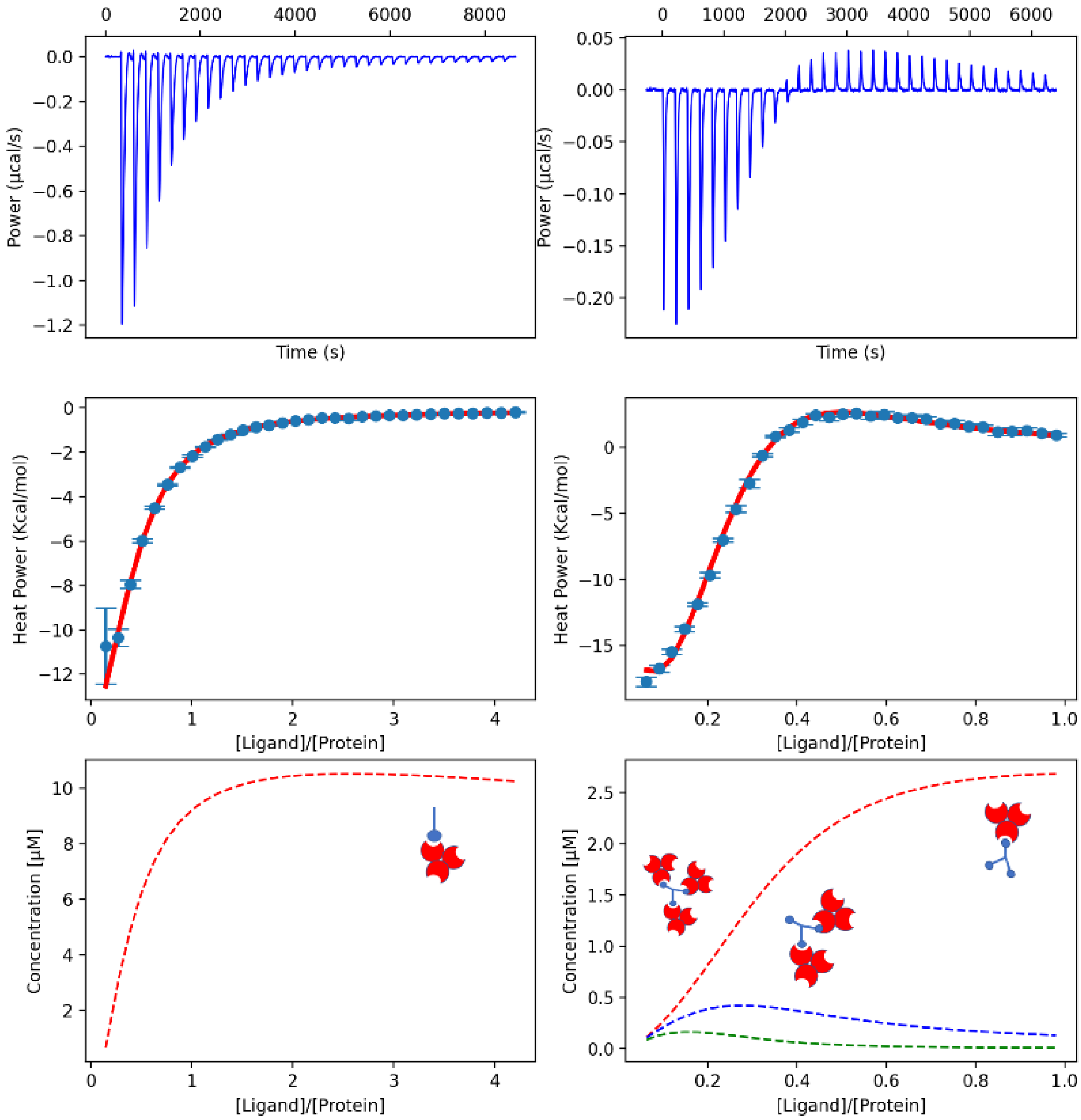

Figure S4. ITC analysis of MGL extracellular domain (81-309) with the monovalent and trivalent glyclusters.

Upper panels show the raw data for titration of ligands into lectins. Middle planes show the corresponding isotherms after the integration of the peaks with the analysis fittings using the binding model described above. Lower panels show the distribution of each complex during the titration (green for M_3A, blue for M_2A and red for MA)

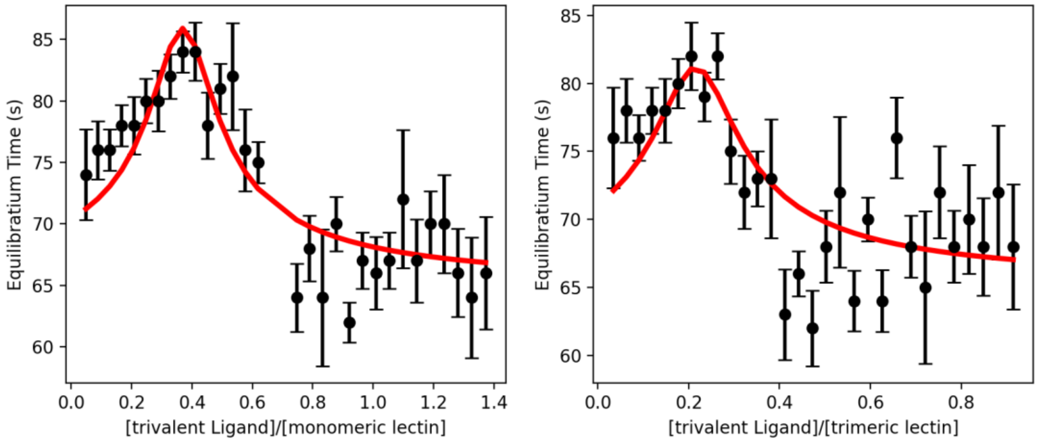

Figure S5. ITC kinetic analysis of MGL CRD (181-277) and MGL extracellular domain (81-309) with the trivalent ligand **1S**. An overall kinetics of the interactions between the trivalent ligand with the monomeric and trimeric lectins were calculated by measuring the equilibrium time of each injection along the ITC titration. The calculated kon and koff values are reported in Table S1. The fitted response time of the calorimeter was 11 ± 1 s, which falls into the expected values for a VP-ITC calorimeter. This confirms the reliability of the kinetic calculations.

Table S1. ITC data. rM: fraction of active protein, rA: fraction of the ligand that is active.

Interaction studied	[Protein cell] (mM)	[Ligand] (monomer, mM)	rM	rA	Ka (M^{-n})	ΔH (cal/mol)	k_{off} (s^{-1})	k_{on} ($M^{-1}s^{-1}$)
CRD GalNAc	0.05	1.0	0.62	1.00	1.4	-14700	--	--
CRD 1S	0.01	0.21	0.62	0.73	$1.482x10^7$<br>$1.486x10^6$<br>$2.12x10^5$	$-6.744x10^3$<br>$-2.612x10^4$<br>$1.085x10^4$	$2.5 x10^{-2}$	$3.7 x10^5$
CRD-Stalk GalNAc	0.04	0.75	0.37	1.00	$1.4x10^5$	-21900	--	--
CRD-Stalk 1S	0.01	0.05	0.37	0.90	$4.001x10^6$<br>$3.080x10^5$<br>$2.591x10^5$	$-3.284x10^3$<br>$-6.689x10^4$<br>$1.000x10^5$	$2.2 x10^{-2}$	$2.5 x10^5$

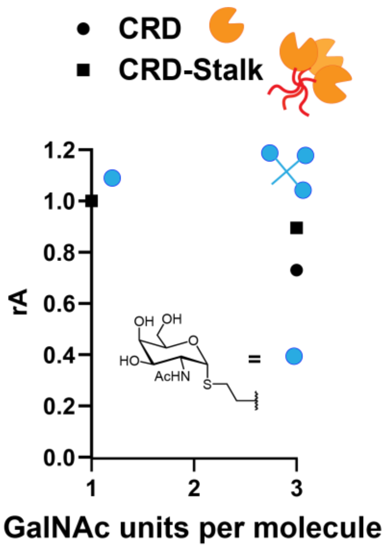

Figure S6. rA trend when increasing ligand valency. . rA is the fraction of multivalent ligands interacting with the protein. rA values lower that 1 can be explained by formation of supramolecular aggregates that prevent all the glycocluster glycans from interaction with the protein carbohydrate recognition domain. rA values of monomer titrated with MGL CRD and MGL extracellular domain are equal to 1.

2 Synthesis general experimental conditions

NMR spectra were recorded with either a 500 or 600 MHz Varian spectrometer at 295 K. The reported values for the chemical shifts δ (ppm) were calibrated to the residual proton or carbon resonance signal of the deuterated solvent, relatively correlated to the corresponding tetramethylsilane signal. Signal multiplicity is assigned as follows: s = singlet, d = doublet, t = triplet, q = quartet, m = multiplet, br = broad. Chemical shifts are reported relative to internal Me_4Si in $CDCl_3$ (δ 0.0), HOD for D_2O (δ 4.84) or CD_2HOD (δ 3.31) for ^1H and $CDCl_3$ (77.16) or CD_3OD (49.05) for ^{13}C. NMR spectra were processed and analysed using MestReNova software. ^1H NMR signals were assigned with the aid of gCOSY. ^{13}C NMR signals were assigned with the aid of APTand/or gHSQCAD and/or gHMBCAD. Coupling constants are reported in Hertz. Low and high-resolution mass spectra were measured on a Waters LCT Premier XE Spectrometer, measuring in both positive and/or negative mode as, using MeCN, H_2O and/or MeOH as solvent. Thin layer chromatography (TLC) was performed on aluminium sheets precoated with silica gel 60 (HF254, E. Merck) and spots visualized by UV and charring with H_2SO_4-EtOH (1:20), cerium molybdate stain, or phosphomolybdic acid stain. Flash chromatography was carried out with silica gel 60 (0.040-0.630 mm, E. Merck or Aldrich) and using a stepwise solvent polarity gradient (starting with the conditions indicated for each experiment and increasing the polarity as required). Chromatography solvents, cyclohexane, EtOAc, CH_2Cl_2 and MeOH were used as obtained from suppliers (Fisher Scientific and Sigma-Aldrich). Anhydrous pyridine was purchased from Sigma Aldrich or other anhydrous solvents were obtained from a Pure Solv™ Solvent Purification System.

2.1 Synthesis of TPE ligand 1

Scheme S1. Synthesis of TPEaa1

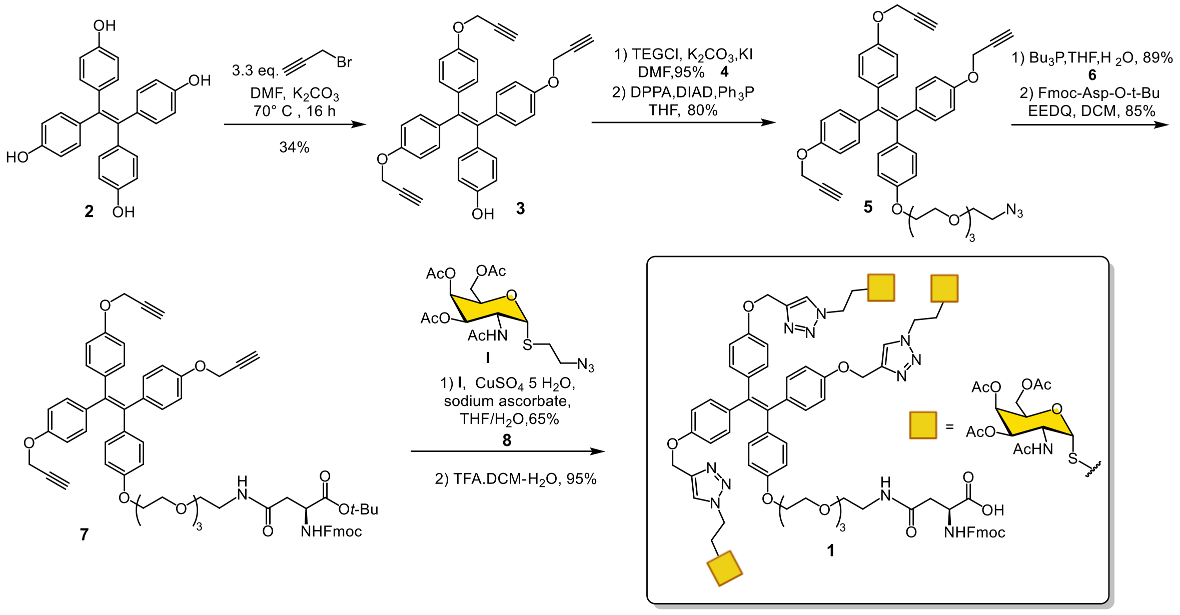

4-(1,2,2-Tris(4-(prop-2-yn-1-yloxy)phenyl)vinyl)phenol (3)

To a solution of **2** (2.50 g, 6.3 mmol) in dry DMF (50 mL) K_2CO_3 (2.79 g, 20.2 mmol) was slowly added, followed by propargyl bromide 80% in toluene (2.29 ml, 20.8 mmol). The reaction mixture was stirred at 70° C for 16 h, cooled to room temperature, diluted with DCM and washed with 1M HCl, brine and water.

The organic phase was dried over Na_2SO_4, filtered and the solvent removed under reduced pressure. Colum chromatography (cy-EtOAc 8.7:1.3, isocratic elution) yielded the title compound (1.1 g, 34%).

^{1}H (500 MHz, Chloroform-d) δ 6.93 (d, J = 8.7 Hz, 6H, aromatic H), 6.87 (d, J = 7.7 Hz, 2H, aromatic H), 6.70 (d, J = 8.0 Hz, 6H, aromatic H), 6.56 (d, J = 8.6 Hz, 2H, aromatic H), 4.62 (s, 6H, CH_2), 3.50 (br s, 1H, OH), 2.50 (s, 3H, propargyl);

^{13}C NMR (125 MHz, Chloroform-d) δ 158.9 (C), 156.1 (C), 154.0 (C), 138.8 (C), 137.6 (C), 132.9 (CH), 132.7 (CH), 114.8 (CH), 114.2 (CH), 78.8 (C), 75.5 (CH), 55.9 (CH_2);

ES-HRMS calcd for $C_{35}H_{25}O_4$ 509.1753, found m/z 509.1759 $[M-H]^-$.

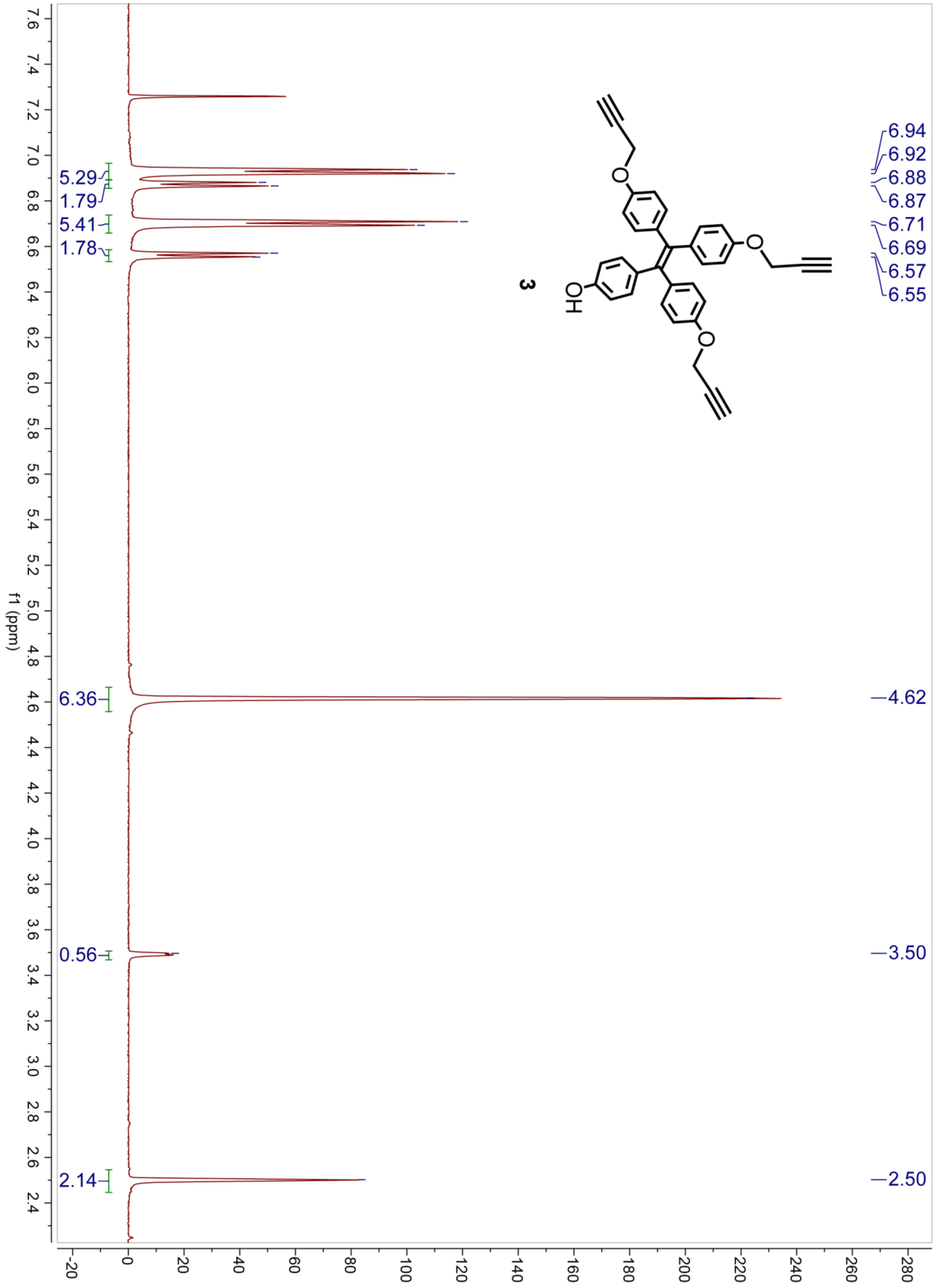

6.94
6.92
6.88
6.87
6.71
6.69
6.57
6.55
4.62
3.50
2.50
5.29
1.79
5.41
1.78
6.36
0.56
2.14
3
OH
f1 (ppm)

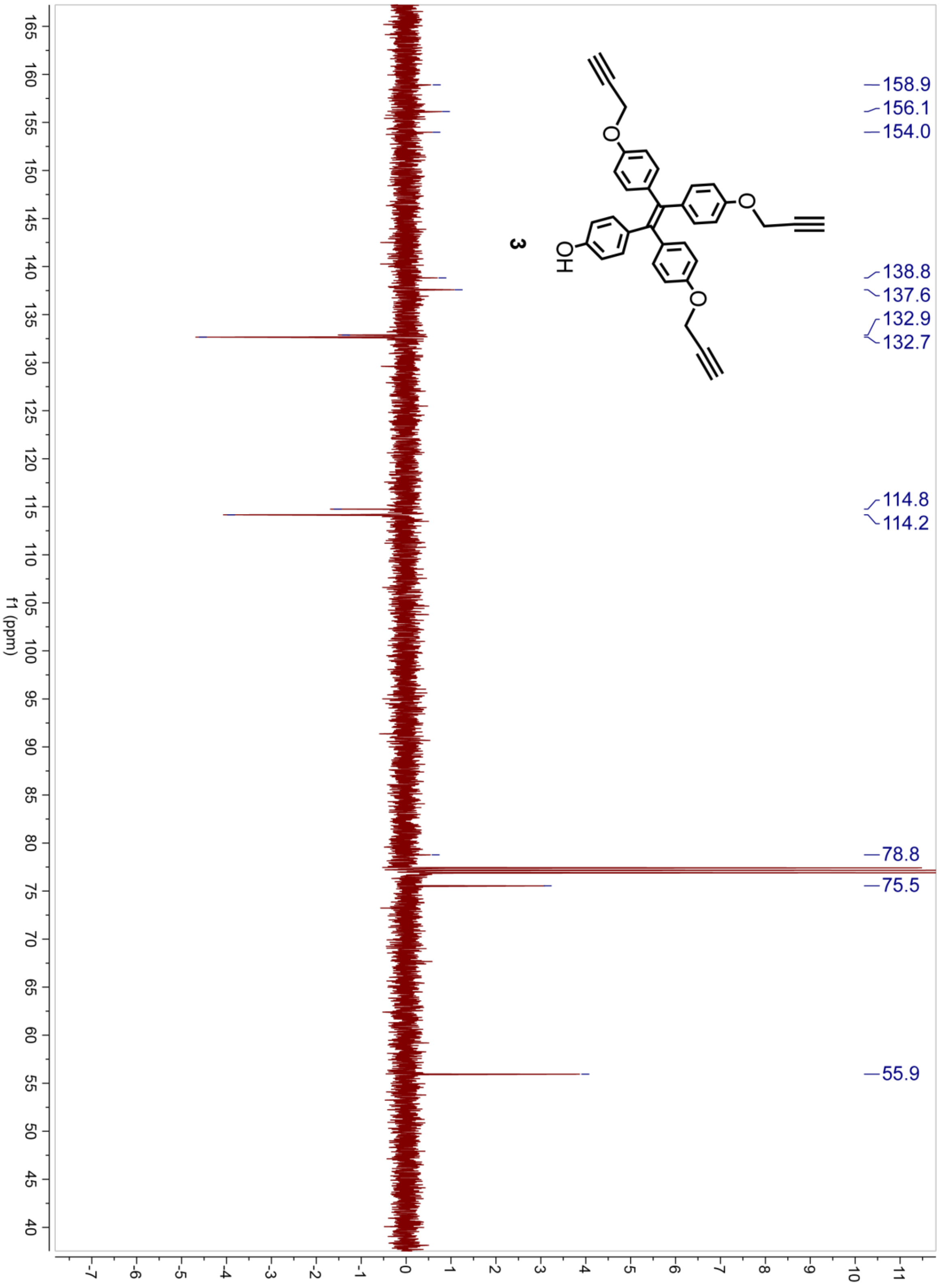

158.9
156.1
154.0
138.8
137.6
132.9
132.7
114.8
114.2
78.8
75.5
55.9
3
OH
f1 (ppm)

2-(2-(2-(4-(1,2,2-Tris(4-(prop-2-yn-1-yloxy)phenyl)vinyl)phenoxy)ethoxy)ethoxy)ethan-1-ol (4)

To a solution of **3** (1 g, 1.95mmol) in dry DMF (50 mL), 2-[2-(2-chloroethoxy)ethoxy]ethanol (697 µL, 4.89 mmol), K_2CO_3 (2.02g, 14.64 mol) and potassium iodine (324 mg, 1.95 mmol) were added respectively. The reaction mixture was stirred at 100 °C for 16 h, allowed to cool to room temperature and then diluted with DCM and sat. aq. NH_4Cl. The organic phase was washed with water and brine and was dried over Na_2SO_4. The solvent was removed under reduced pressure to yield the title compound (1.1 g, 95%).

^{1}H NMR (500 MHz, Chloroform-d) δ 6.97 – 6.87 (m, 8H, Ar), 6.70 (d, J = 8.7 Hz, 6H, O-Ar), 6.64 (d, J = 8.1 Hz, 2H, O-Ar), 4.61 (s, 6H, CH_2), 4.06 (t, J = 4.3 Hz, 2H, CH_2), 3.82 (t, J = 4.3 Hz, 2H, CH_2), 3.75 – 3.66 (m, 6H, CH_2), 3.65 – 3.55 (m, 2H, CH_2), 2.54 – 2.47 (m, 2H, propargyl), 2.42 – 2.36 (m, 1H, propargyl);

^{13}C NMR (125 MHz, Chloroform-d) δ 157.1 (C), 156.1 (C), 138.8 (C), 138.5 (C), 137.6 (C), 137.6 (C), 137.6 (C), 136.9 (C), 132.6 (CH), 114.1 (CH), 113.9 (CH), 78.7 (C), 75.6 (CH), 75.5 (CH), 72.6 (CH_2), 70.9 (CH_2), 70.5 (CH_2), 69.9 (CH_2), 67.3 (CH_2), 61.9 (CH_2), 55.9 (CH_2);

ES-HRMS calcd for $C_{41}H_{37}O_7$ 641.2539, found m/z 641.2534 $[M-H]^-$.

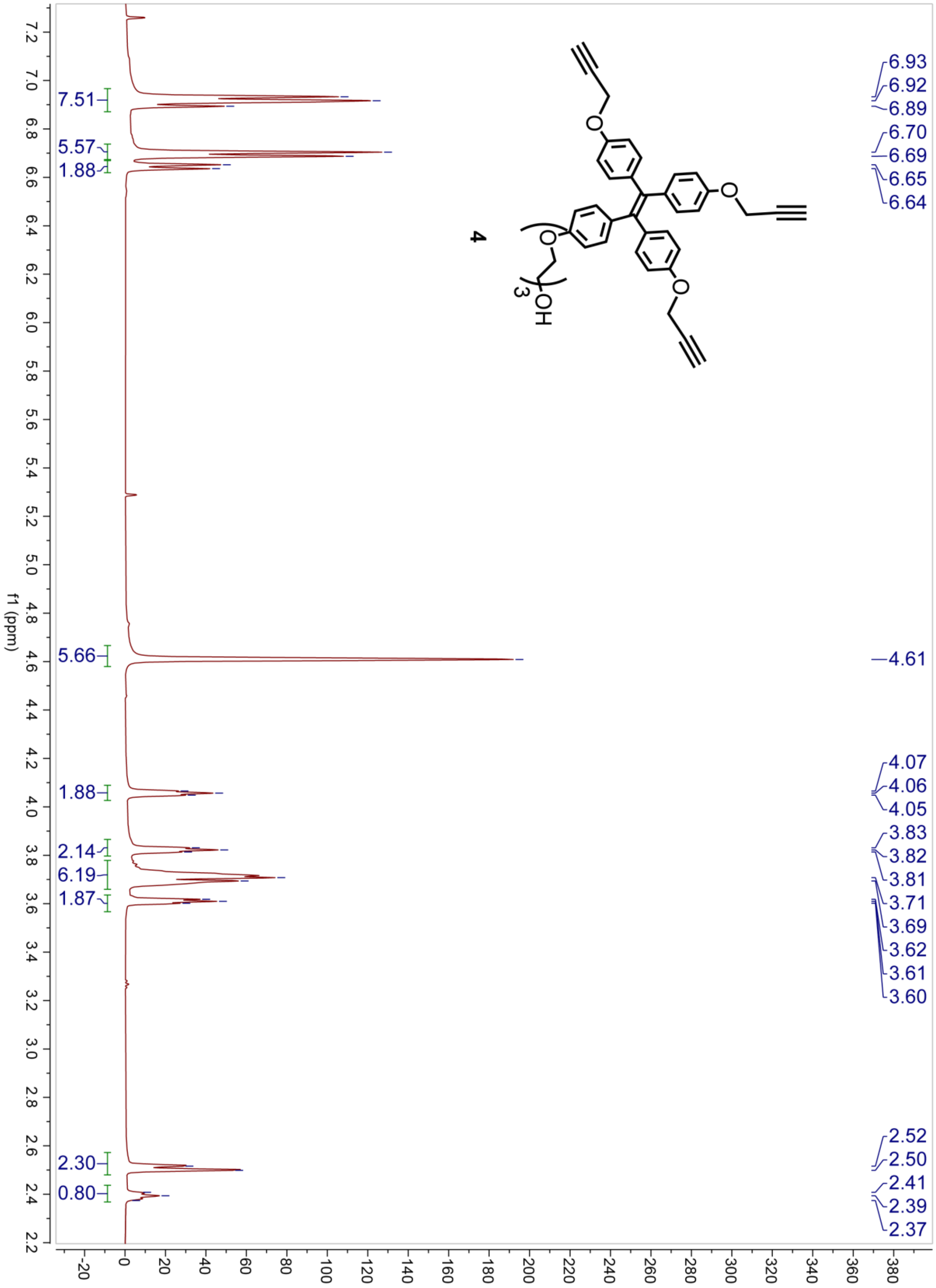

4
OH
3
O
6.93
6.92
6.89
6.70
6.69
6.65
6.64
4.61
4.07
4.06
4.05
3.83
3.82
3.81
3.71
3.69
3.62
3.61
3.60
2.52
2.50
2.41
2.39
2.37
7.51
5.57
1.88
5.66
1.88
2.14
6.19
1.87
2.30
0.80
f1 (ppm)

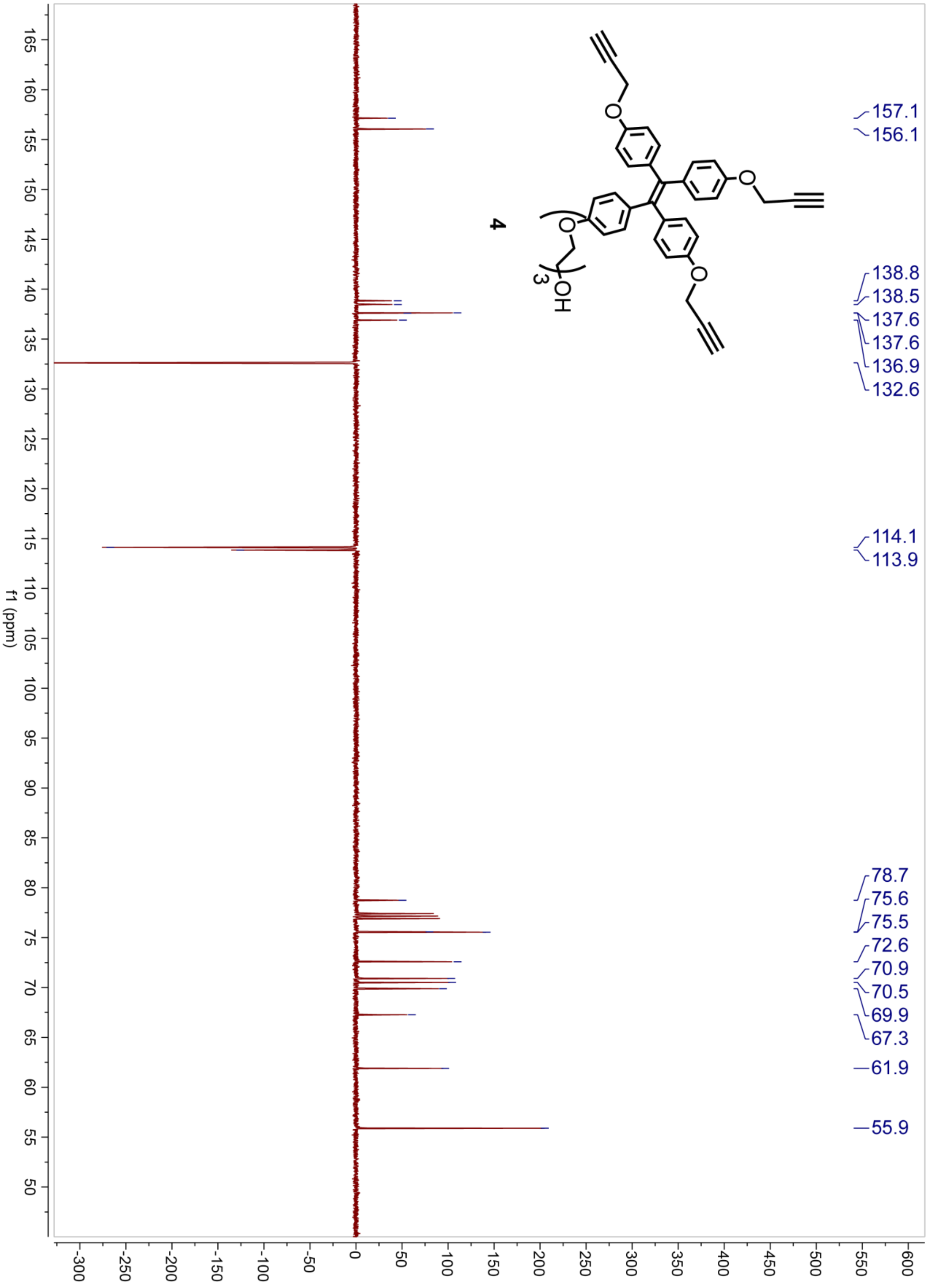
157.1
156.1
138.8
138.5
137.6
137.6
136.9
132.6
114.1
113.9
78.7
75.6
75.5
72.6
70.9
70.5
69.9
67.3
61.9
55.9
4
f1 (ppm)

4,4',4''-(2-(4-(2-(2-(2-azidoethoxy)ethoxy)ethoxy)phenyl)ethene-1,1,2-triyl)tris((prop-2-yn-1-yloxy)benzene) (5)

To cooled solution of **4** (600 mg, 0.93 mmol, 1 eq.) at 0°C in THF (10 mL), PPh_3 (783 mg, 3mmol, 3.2 eq.) and DIAD (603 mg, 3 mmol, 3,2 eq.) were added. The reaction mixture was stirred at 0°C until TLC confirmed full conversion of the starting material (cy/EtOAc 7:3). *(NOTE: reagent complete consumption takes ca 1h. In case PPh_3 is still present, add DIAD or vice versa).* DPPA (847 mg, 3.1 mmol, 3.3 eq.) was then added and the reaction was allowed to attain room temperature and was then stirred for a further 2 h. The reaction was diluted with EtOAc (100 mL) and washed with H_2O (3 x 15 mL). The organic phase was dried over Na_2SO_4 and the solvent was removed under reduced pressure. Column chromatography (cy-EtOAc 6:4, isocratic elution) yielded the title compound (500 mg, 80%).

^{1}H NMR (500 MHz, Chloroform-d) δ 6.96 – 6.88 (m, 8H, Ar), 6.70 (d, J = 7.9 Hz, 6H, O-Ar), 6.64 (d, J = 8.7 Hz, 2H, O-Ar), 4.61 (d, J = 2.2 Hz, 6H, CH_2), 4.06 (t, J = 5.2 Hz, 2H, CH_2), 3.83 (t, J = 5.0 Hz, 2H, CH_2), 3.75 – 3.71 (m, 2H, CH_2), 3.70 – 3.65 (m, 4H, CH_2), 3.38 (t, J = 5.0 Hz, 2H, CH_2), 2.53 – 2.48 (m, 3H, propargyl);

^{13}C NMR (125 MHz, Chloroform-d) δ 157.2 (C), 156.1 (C), 138.9 (C), 138.5 (C), 137.7 (C), 136.9 (C), 132.6 (CH), 114.1(CH), 113.9 (CH), 78.8 (C), 75.6 (CH), 75.5 (CH), 71.0 (CH_2), 70.9 (CH_2), 70.2 (CH_2), 70.0 (CH_2), 67.3 (CH_2), 55.9 (CH_2), 50.8 (CH_2);

ES-HRMS calcd for $C_{41}H_{37}N_3O_6Na$ 690.2580, found m/z 690.2584 $[M+Na]^+$.

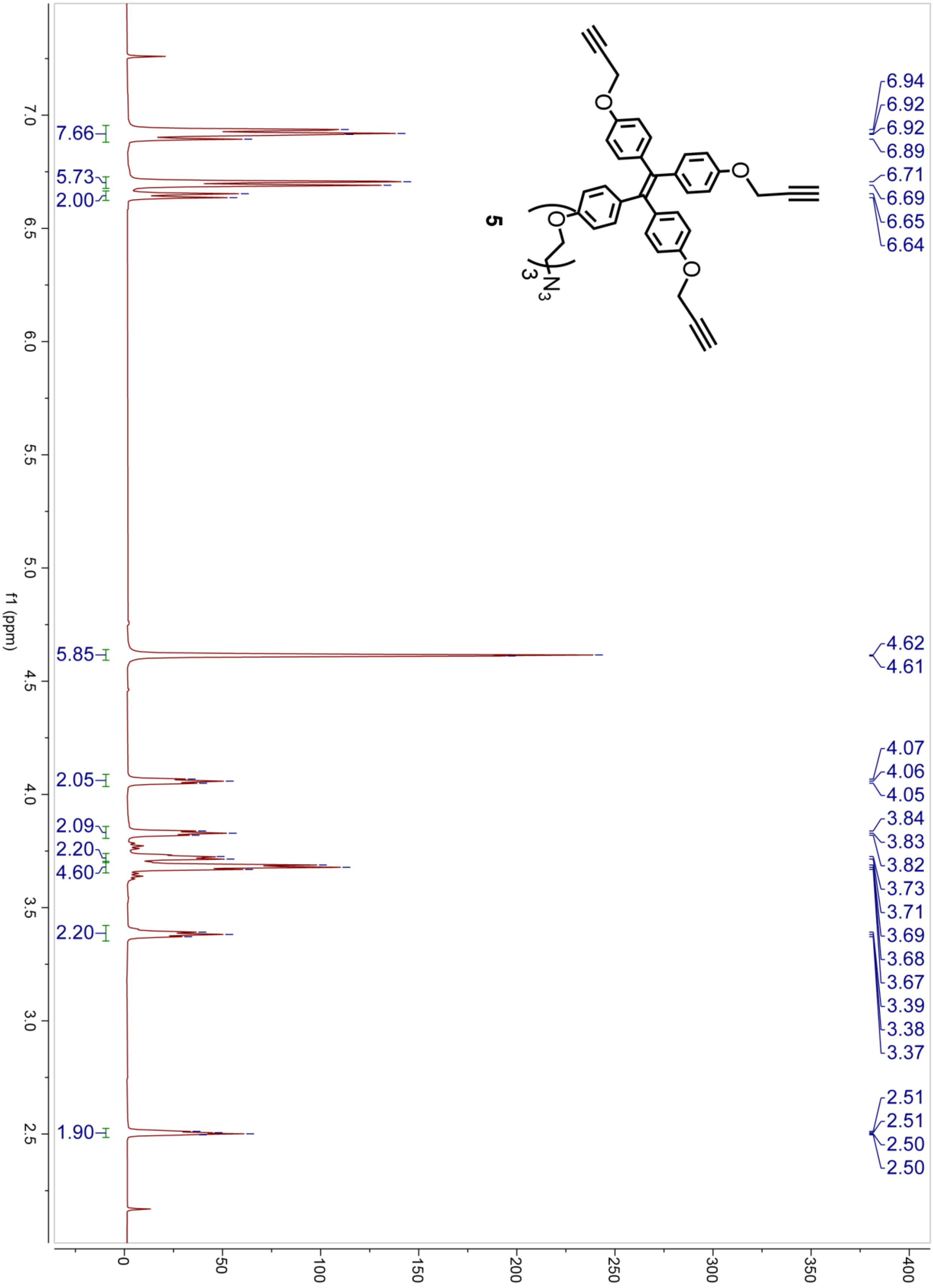
5
N3
3
6.94
6.92
6.92
6.89
6.71
6.69
6.65
6.64
4.62
4.61
4.07
4.06
4.05
3.84
3.83
3.82
3.73
3.71
3.69
3.68
3.67
3.39
3.38
3.37
2.51
2.51
2.50
2.50
7.66
5.73
2.00
5.85
2.05
2.09
2.20
4.60
2.20
1.90
f1 (ppm)

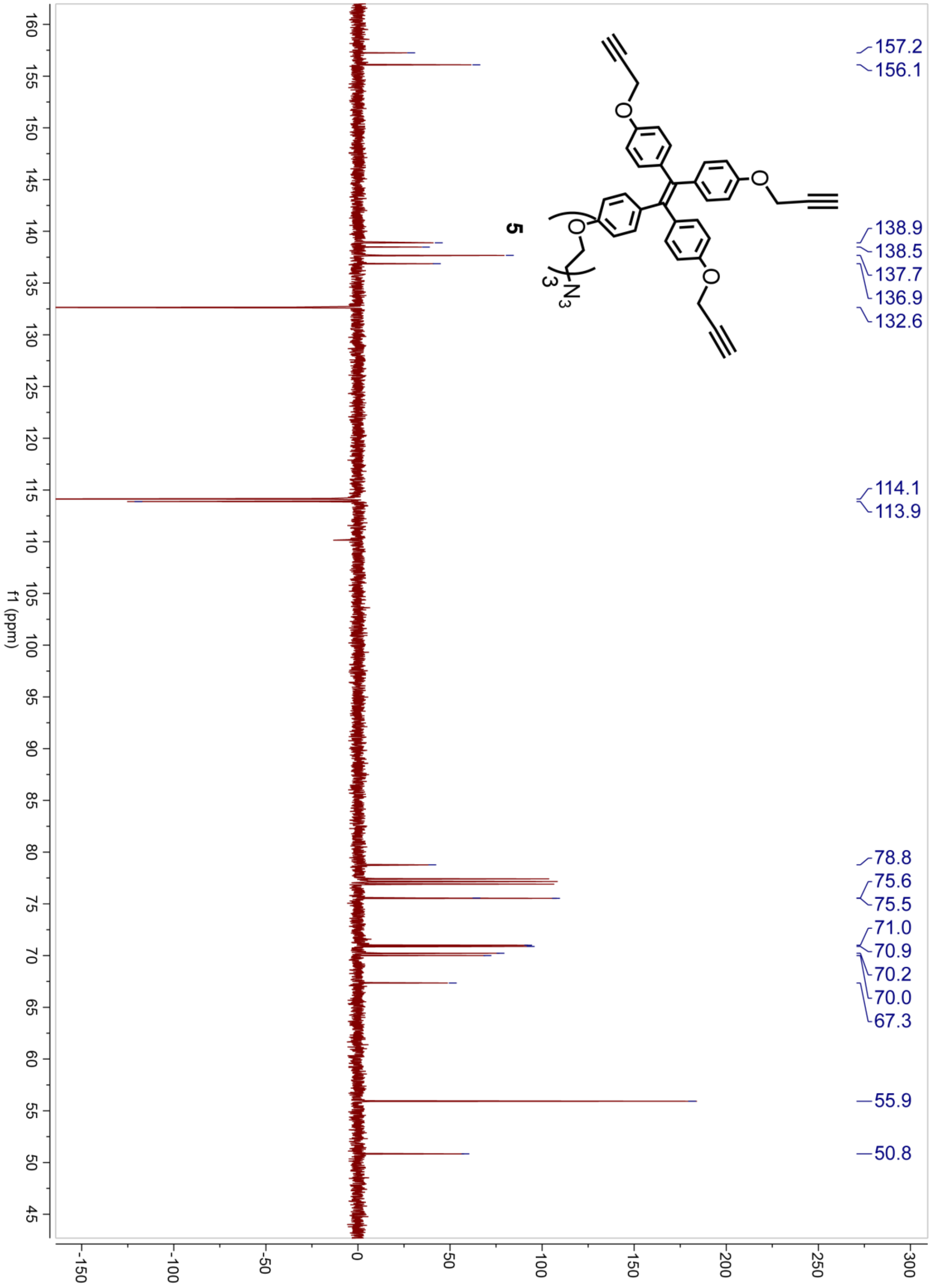
157.2
156.1
138.9
138.5
137.7
136.9
132.6
114.1
113.9
78.8
75.6
75.5
71.0
70.9
70.2
70.0
67.3
55.9
50.8
5
f1 (ppm)

2-(2-(2-(4-(1,2,2-tris(4-(prop-2-yn-1-yloxy)phenyl)vinyl)phenoxy)ethoxy)ethoxy)ethan-1-amine (6)

To a solution of **5** (500 mg, 0.75 mmol) in THF-H_2O (5 mL 9:1), PBu_3 (303 mg, 1.5 mmol) was added. The reaction mixture was stirred at 25°C for 2 h. The solvent was removed in vacuo. Colum chromatography ($CHCl_3$-MeOH-NH_4OH 95:05→ 85:13:02, gradient elution) yielded the titled compound (427 mg, 85%).

^{1}H NMR (500 MHz, Chloroform-d) δ 6.97 – 6.86 (m, 8H, Ar), 6.70 (d, J = 8.6 Hz, 6H, O-Ar), 6.66 – 6.60 (m, 2H, O-Ar), 4.72 – 4.52 (m, 6H, CH_2), 4.11 – 3.99 (m, 2H, CH_2), 3.85 – 3.77 (m, 2H, CH_2), 3.74 – 3.69 (m, 2H, CH_2), 3.68 – 3.61 (m, 2H, CH_2), 3.52 (t, J = 5.2 Hz, 2H, CH_2), 2.87 (t, J = 5.1 Hz, 2H, CH_2), 2.61 – 2.42 (m, 3H, propargyl);

^{13}C NMR (125 MHz, Chloroform-d) δ 157.1(C), 155.9 (C), 138.7 (C), 138.3 (C), 137.5 (C), 136.7 (C), 132.5 (CH), 114.0 (CH), 113.7 (CH), 78.6 (C), 75.4 (CH), 75.4 (CH), 73.2(CH_2), 70.8 (CH_2), 70.3 (CH_2), 69.8 (CH_2), 67.2 (CH_2), 41.7 (CH_2); ES-HRMS calcd for $C_{41}H_{40}NO_6$ 642.2777, found m/z 642.2780 $[M+H]^+$.

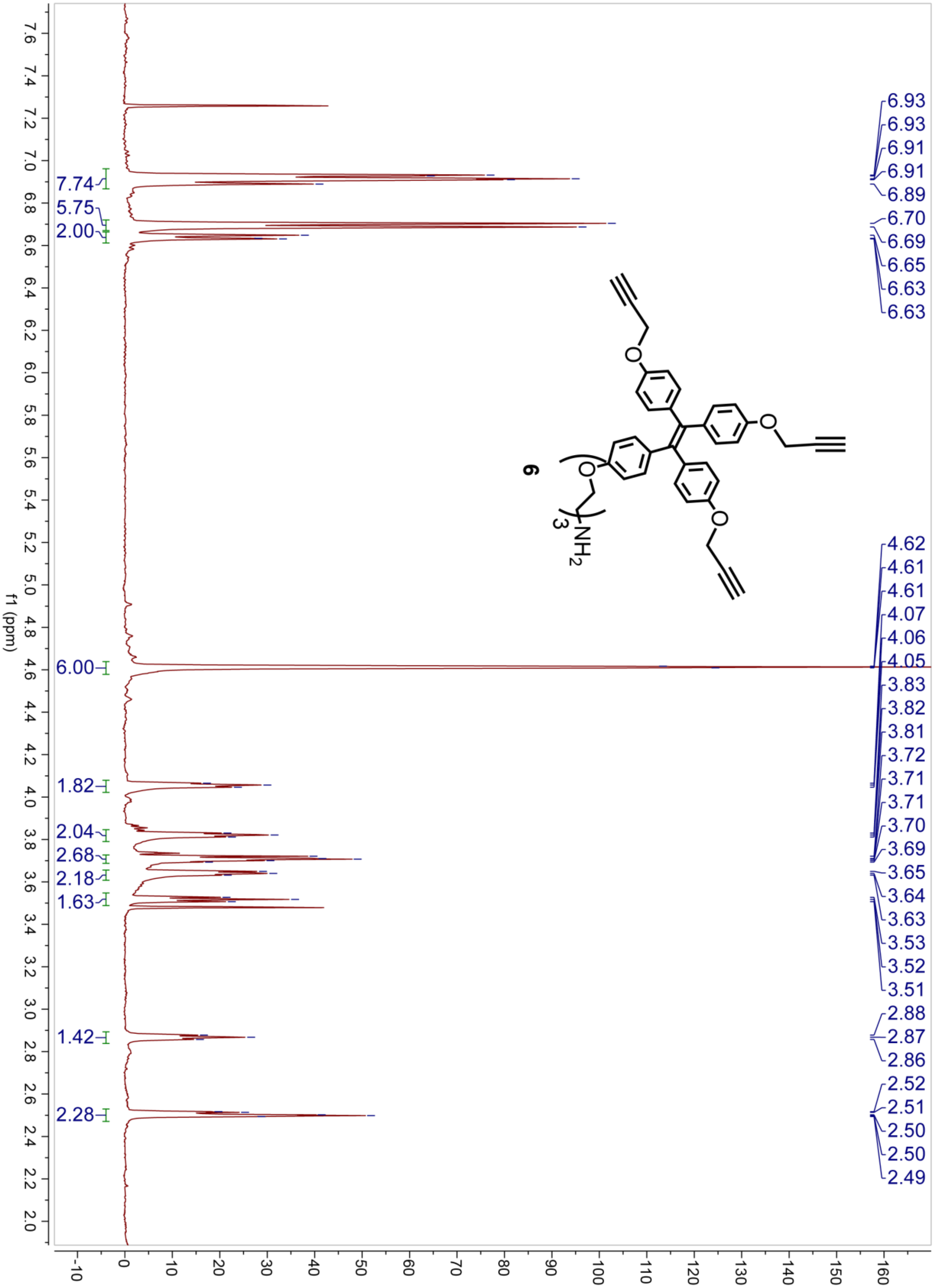
6.93
6.93
6.91
6.91
6.89
6.70
6.69
6.65
6.63
6.63
4.62
4.61
4.61
4.07
4.06
4.05
3.83
3.82
3.81
3.72
3.71
3.71
3.70
3.69
3.65
3.64
3.63
3.53
3.52
3.51
2.88
2.87
2.86
2.52
2.51
2.50
2.50
2.49
7.74
5.75
2.00
6.00
1.82
2.04
2.68
2.18
1.63
1.42
2.28
6
NH2
3
f1 (ppm)

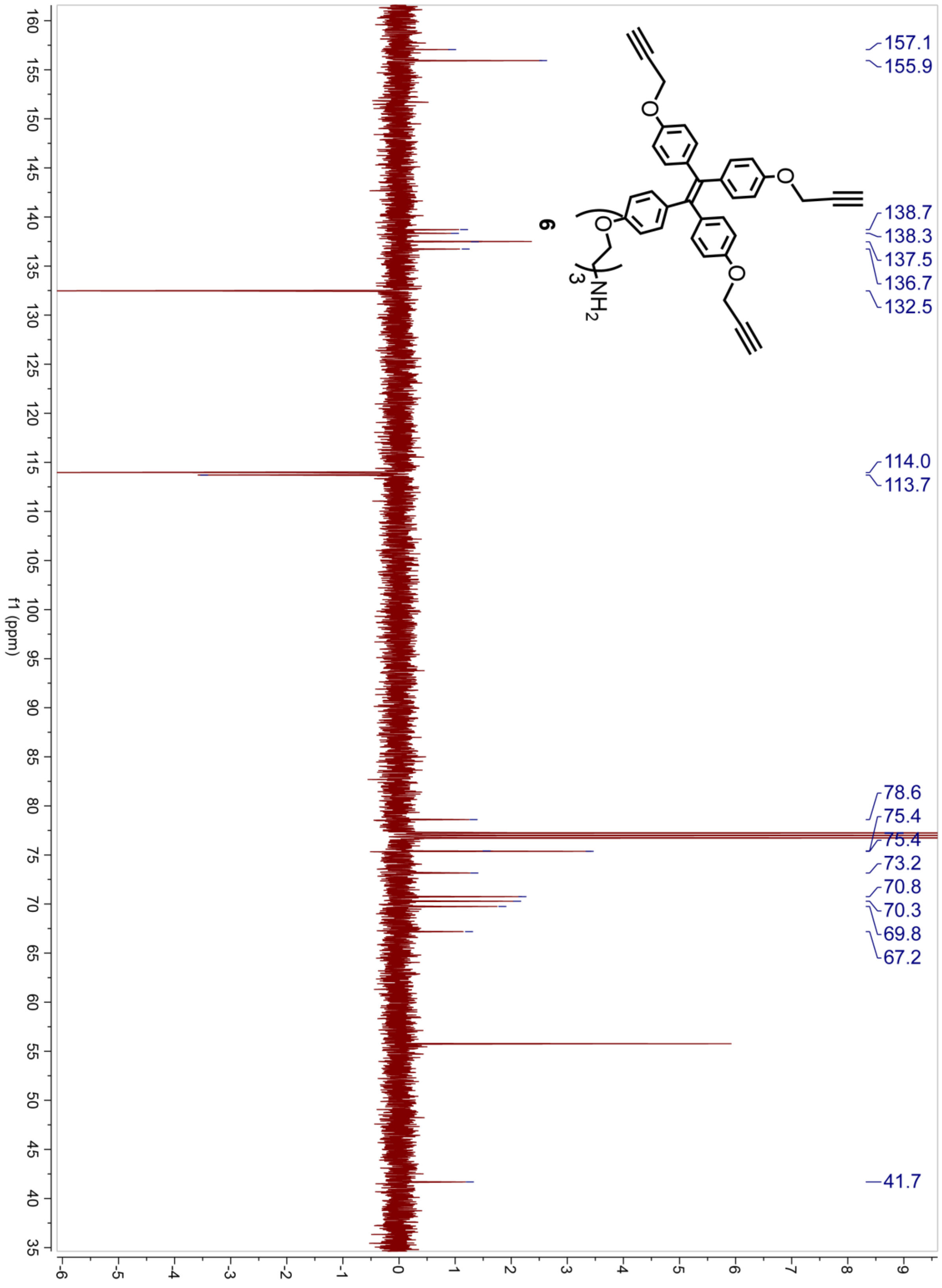
157.1
155.9
138.7
138.3
137.5
136.7
132.5
114.0
113.7
78.6
75.4
75.4
73.2
70.8
70.3
69.8
67.2
41.7
6
NH2
f1 (ppm)

tert-butyl-N-(((9H-fluoren-9-yl)methoxy)carbonyl)-N-(2-(2-(2-(4-(1,2,2-tris(4-(prop-2-yn-1-yloxy)phenyl)vinyl)phenoxy)ethoxy)ethoxy)ethyl)-L-asparaginate (7)

To a solution of **6** (363 mg, 0.57 mmol) in dry DCM (20 mL), Fmoc-L-Asp O*t*-Bu (234 mg, 0.57 mmol) and EEDQ (155 mg, 0.63 mmol) were added. The reaction mixture was stirred at 30 °C for 16 h. The solvent was removed in vacuo. Colum chromatography (cy-EtOAc 8:2→ 1:1, gradient elution) yielded the titled compound (524 mg, 89%).

^{1}H NMR (500 MHz, Chloroform-d) δ 7.75 (d, J = 7.6 Hz, 2H, Ar Fmoc), 7.58 (d, J = 7.3 Hz, 2H, Ar Fmoc), 7.39 (t, J = 7.5 Hz, 2H, Ar Fmoc), 7.30 (t, J = 7.4 Hz, 2H, Ar Fmoc), 6.96 – 6.85 (m, 8H, Ar), 6.79 (br s, 1H, NH), 6.74 – 6.65 (m, 6H, O-Ar), 6.61 (d, J = 8.7 Hz, 2H, O-Ar), 5.93 (d, J = 8.1 Hz, 1H, NH), 4.67 – 4.56 (m, 6H, CH_2), 4.51 (s, 1H, CH Asp), 4.41 (t, J = 6.1 Hz, 2H, CH Fmoc), 4.25 – 4.18 (m, 1H, CH Fmoc), 4.01 (t, J = 4.7 Hz, 2H, CH_2), 3.77 (t, J = 4.7 Hz, 2H, CH_2), 3.66 (d, J = 5.1 Hz, 2H, CH_2), 3.61 (d, J = 4.2 Hz, 2H, CH_2), 3.54 (d, J = 4.9 Hz, 2H, CH_2), 3.49 – 3.39 (m, 2H, CH_2), 2.94 – 2.82 (m, 1H, CH_2 Asp), 2.60 (dd, J = 16.7, 6.2 Hz, 1H, CH_2 Asp), 2.50 (d, J = 2.4 Hz, 3H, propargyl), 1.44 (s, 9H, *t*-butyl);

^{13}C NMR (125 MHz, Chloroform-d) δ 157.0 (C), 155.9 (C), 155.9 (C), 143.7 (C), 141.3 (C), 138.7 (C), 138.3 (C), 137.5 (C), 136.7 (C), 132.5 (CH), 127.8 (CH), 127.1 (CH), 125.0 (CH), 120.0 (CH), 114.0 (CH), 113.7 (CH), 78.6 (C), 75.4 (CH), 75.4 (CH), 70.7 (CH_2), 70.4 (CH_2), 69.7 (CH_2), 69.6 (CH_2), 67.1 (CH_2), 55.7 (CH_2), 51.3 (CH, HSQC), 47.1 (CH), 39.5 (CH_2), 28.0 (CH_3);

ES-HRMS calcd for $C_{64}H_{63}N_2O_{11}$ 1035.4432, found m/z 1035.4441 $[M+H]^+$.

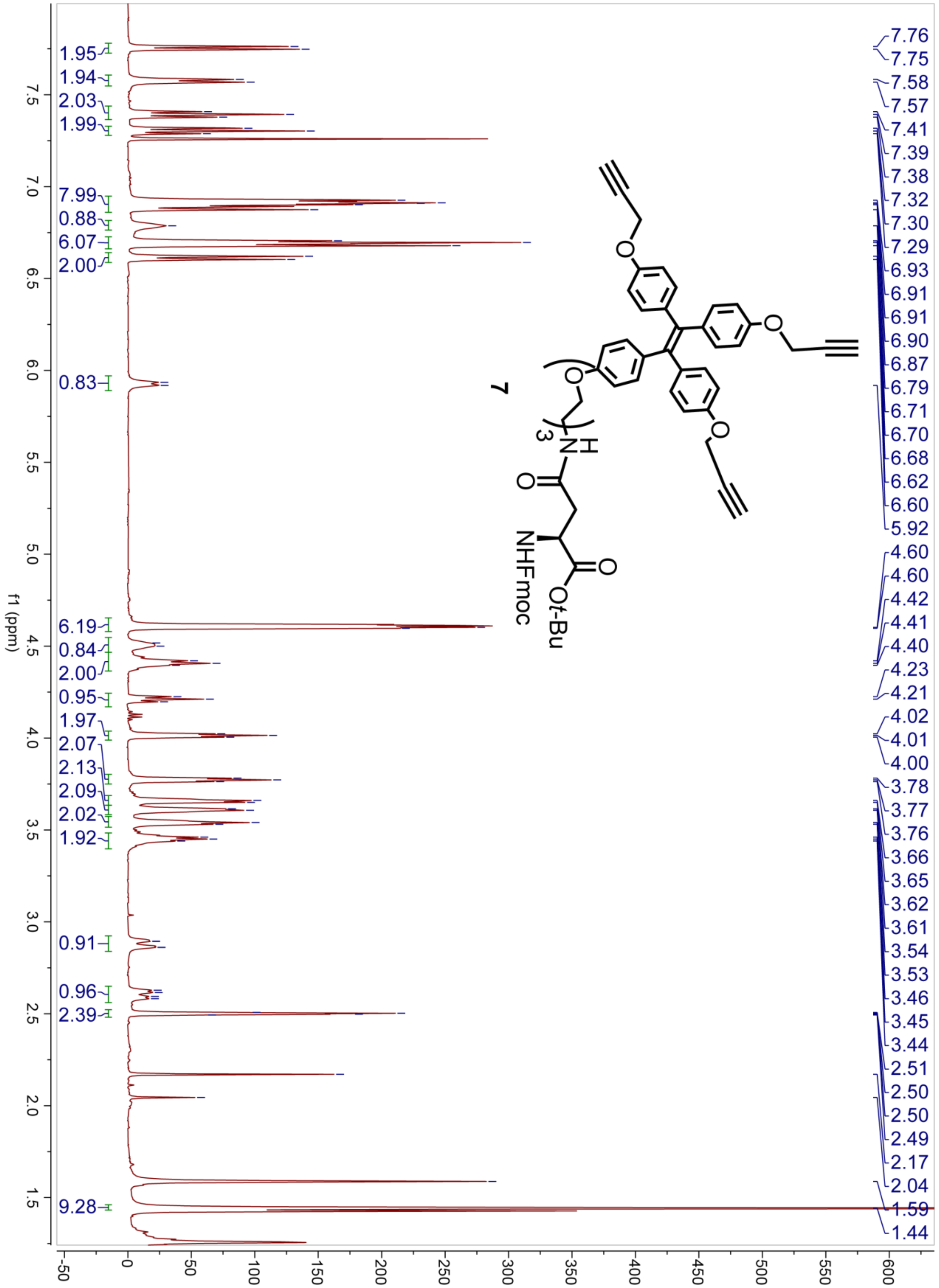

7
NHFmoc
Ot-Bu
O
H
N
3
f1 (ppm)
7.76
7.75
7.58
7.57
7.41
7.39
7.38
7.32
7.30
7.29
6.93
6.91
6.91
6.90
6.87
6.79
6.71
6.70
6.68
6.62
6.60
5.92
4.60
4.60
4.42
4.41
4.40
4.23
4.21
4.02
4.01
4.00
3.78
3.77
3.76
3.66
3.65
3.62
3.61
3.54
3.53
3.46
3.45
3.44
2.51
2.50
2.50
2.49
2.17
2.04
1.59
1.44
1.95
1.94
2.03
1.99
7.99
0.88
6.07
2.00
0.83
6.19
0.84
2.00
0.95
1.97
2.07
2.13
2.09
2.02
1.92
0.91
0.96
2.39
9.28

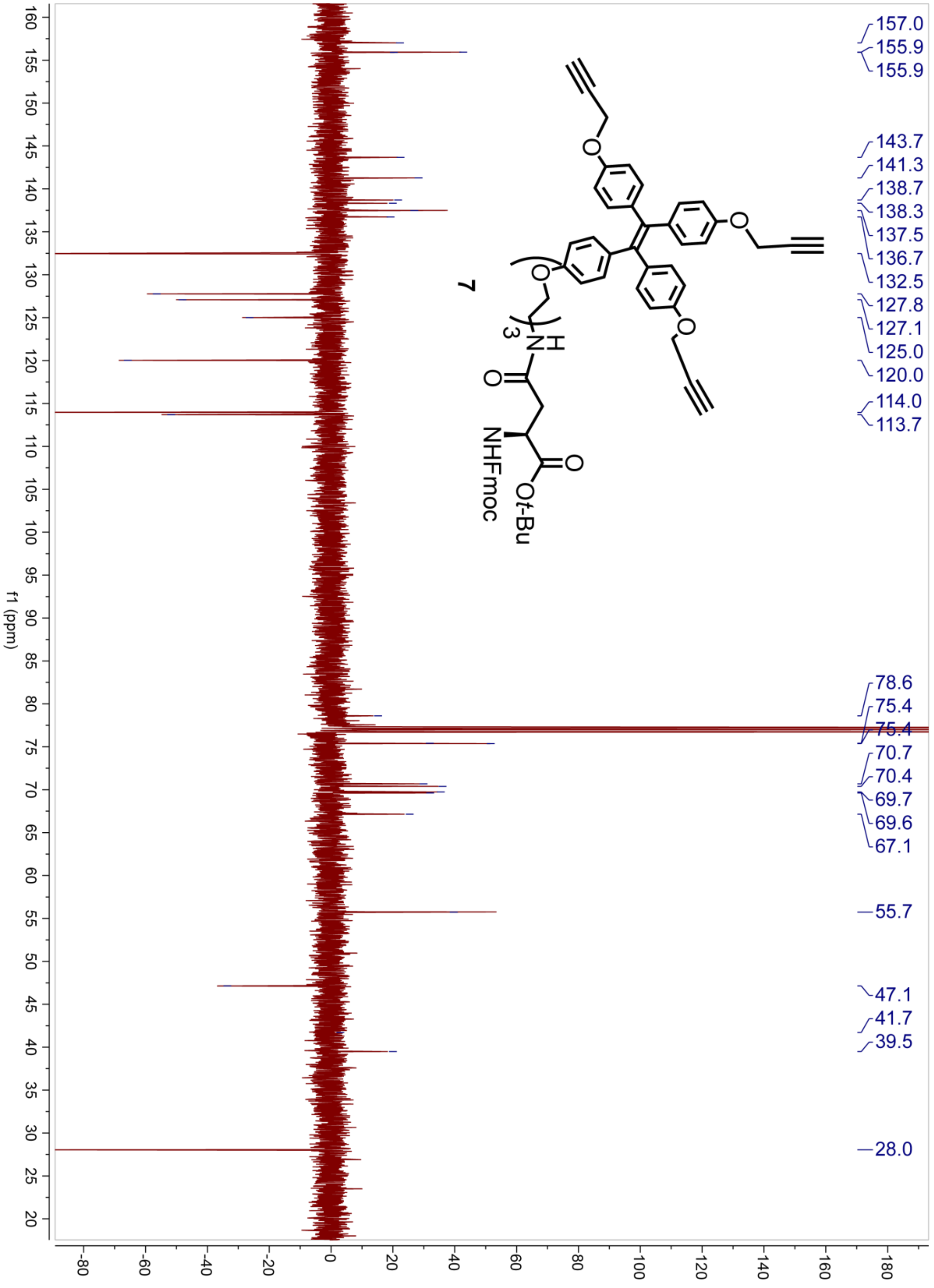
157.0
155.9
155.9
143.7
141.3
138.7
138.3
137.5
136.7
132.5
127.8
127.1
125.0
120.0
114.0
113.7
78.6
75.4
75.4
70.7
70.4
69.7
69.6
67.1
55.7
47.1
41.7
39.5
28.0
f1 (ppm)
7
NHFmoc
Ot-Bu

Acetylated glycocluster (8)

To a solution of **7** (374 mg, 0.36 mmol) in degassed THF-H_2O (50 mL, 1:1), **I** (548 mg, 1.27 mmol), sodium ascorbate (62 mg, 0.31 mmol) and $CuSO_4 \cdot 5H_2O$ (74 mg, 0.3 mmol) were added. The reaction mixture was stirred at 35 °C for 16 h. THF was removed in vacuo and water phase was extracted with DCM (3 x 25 mL). The organic phase was dried over Na_2SO_4, filtered and the solvent was removed under reduced pressure. Column chromatography (DCM-MeOH 98:2→ 95:05) yielded the titled compound (631 mg, 78%).

^{1}H NMR (500 MHz, chloroform-*d*) δ 7.74 (d, *J* = 7.5 Hz, 2H, Fmoc aromatic protons), 7.66 (s, 3H, triazole), 7.59 (dd, *J* = 7.2, 2.5 Hz, 2H, Fmoc protons), 7.38 (t, *J* = 7.4 Hz, 2H, Fmoc protons), 7.28 (t, *J* = 7.3 Hz, 2H, Fmoc protons), 6.96 - 6.85 (m, 8H, aromatic protons), 6.70 (t, *J* = 7.7 Hz, 6H, aromatic protons), 6.63 (d, *J* = 7.2, 2H, , aromatic protons), 6.35 (brs, 1H, NH), 6.11 (d, *J* = 8.1 Hz, 1H, NH), 6.01 (d, *J* = 8.0 Hz, 1H, NHAc), 5.97 (d, *J* = 8.2 Hz, 1H, NHAc), 5.84 (d, *J* = 7.9 Hz, 1H, NHAc), 5.70 – 5.63 (m, 3H, H-1), 5.38 (br s, 3H, H-4), 5.13 (s, 2H, CH_2), 5.12 (s, 2H, CH_2), 5.11 (s, 2H, CH_2), 5.00 (dd, *J* = 11.8, 3.1 Hz, 3H, H-3), 4.77 – 4.68 (m, 3H, H-2), 4.65 – 4.54 (m, 6H, CH_2), 4.51 (q, *J* = 7.8, 6.5 Hz, 3H, H-5), 4.44 (dt, *J* = 8.4, 4.6 Hz, 1H, CH Asp), 4.41 – 4.36 (m, 1H, CH_2 Fmoc), 4.33 – 4.25 (m, 1H, CH_2 Fmoc), 4.21 (t, *J* = 7.1 Hz, 1H,CH Fmoc), 4.17 – 4.07 (m, 6H, H-6), 4.05 – 4.02 (m, 2H, CH_2 TEG), 3.80 – 3.76 (m, 2H, CH_2 TEG), 3.64 (d, *J* = 4.7 Hz, 2H, CH_2 TEG), 3.59 (d, *J* = 4.5 Hz, 2H, CH_2 TEG), 3.53 (t, *J* = 5.1 Hz, 2H, CH_2 TEG), 3.42 (t, *J* = 5.3 Hz, 2H, CH_2 TEG), 3.21 – 3.11 (m, 3H, CH_2), 3.10 – 2.97 (m, 3H, $H_{20}{}^2$), 2.83 (dd, *J* = 15.5, 4.9 Hz, 1H, CH_2 Asp), 2.66 (dd, *J* = 15.7, 4.2 Hz, 1H, CH_2 Asp), 2.16 (s, 9H, NHAc), 1.99 (s, 9H, OAc), 1.97(s, 9H, OAc) ,1.96 (s, 9H, OAc), 1.45 (s, 9H, *t*-Bu);

^{13}C NMR (125 MHz, chloroform-*d*) δ 171.0 (C), 170.6 (C), 170.6 (C), 170.5 (C), 170.4 (C), 170.4 (C), 170.2 (C), 170.1 (C), 156.9 (C), 156.6 (C), 156.5 (C), 144.3 (C), 143.9 (C), 141.2 (C), 138.6 (C) , 138.4 (C), 137.3 (C), 137.2 (C), 136.8 (C), 132.5 (CH, aromatic), 127.7 (CH, Fmoc aromatic), 127.1 (CH, Fmoc aromatic), 125.2 (CH, Fmoc aromatic), 123.1 (CH, Fmoc aromatic), 119.9 (CH, triazole), 113.9 (CH, aromatic) , 113.9 (CH, aromatic), 113.7 (CH, aromatic), 85.1 (CH, C-1), 82.2 (C), 70.6 (CH_2, TEG), 70.3 (CH_2, TEG), 69.7 (CH_2, TEG), 68.2 (CH, C-3), 67.6 (CH, C-5), 67.1 (CH, C-4), 62.0 (CH_2, C-6), 61.9 (CH_2, C-6), 51.5 (CH, Asp), 49.6 (CH_2), 48.5

(CH, C-2), 47.1 (CH, Fmoc), 39.3 (CH_2, TEG), 37.9 (CH_2 Asp), 31.1 (CH_2), 27.9 (CH_3, *t*-Bu), 23.2 (CH_3, NHAc), 20.7 (CH_3, OAc), 20.7 (CH_3, OAc), 20.6 (CH_3, OAc);

ES-HRMS calcd for $C_{112}H_{134}N_{14}NaO_{35}S_3$ 2353.8196, found m/z 2353.8196 $[M+Na]^+$.

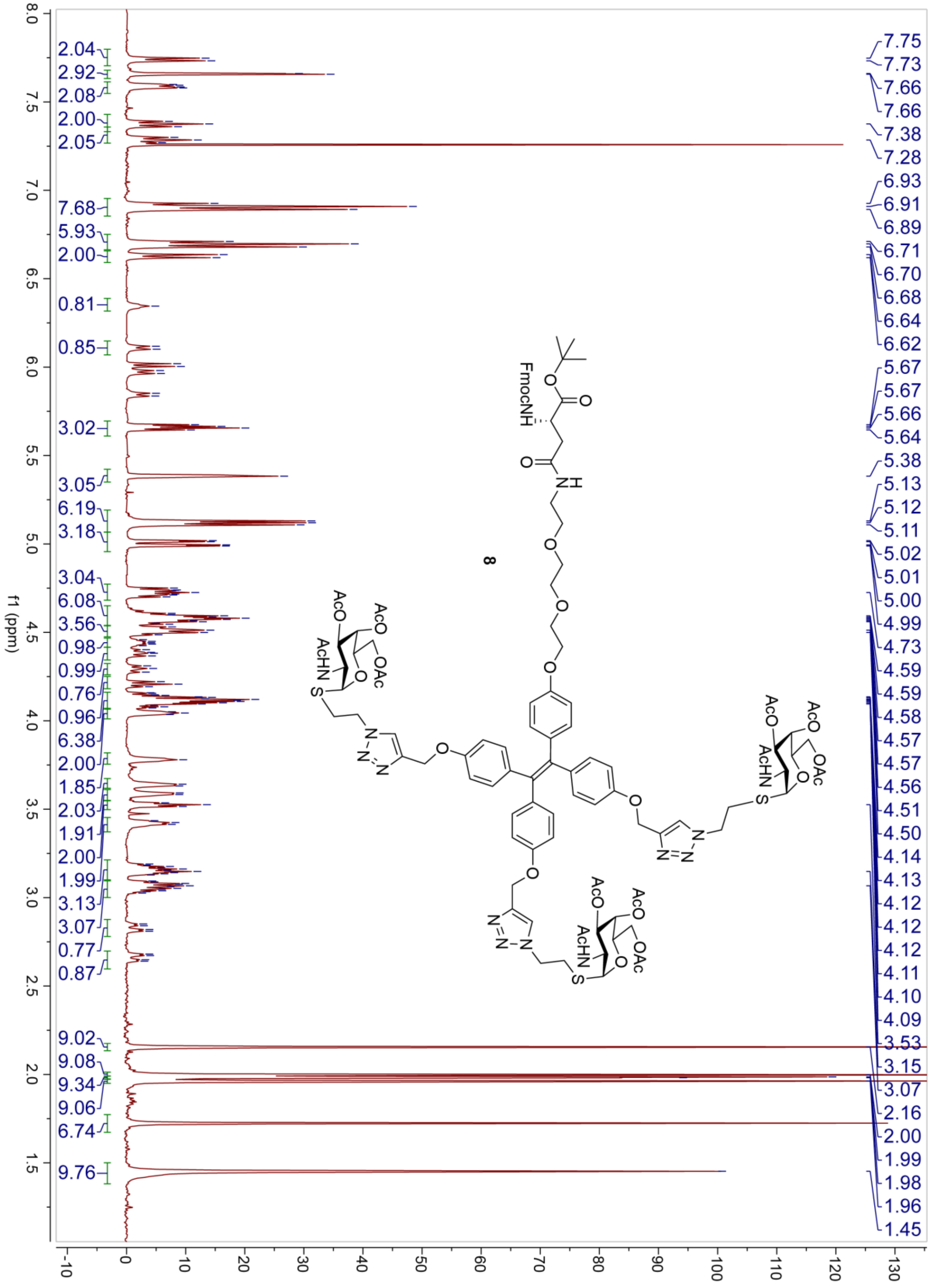
f1 (ppm)
8
FmocNH
AcO
AcHN
OAc

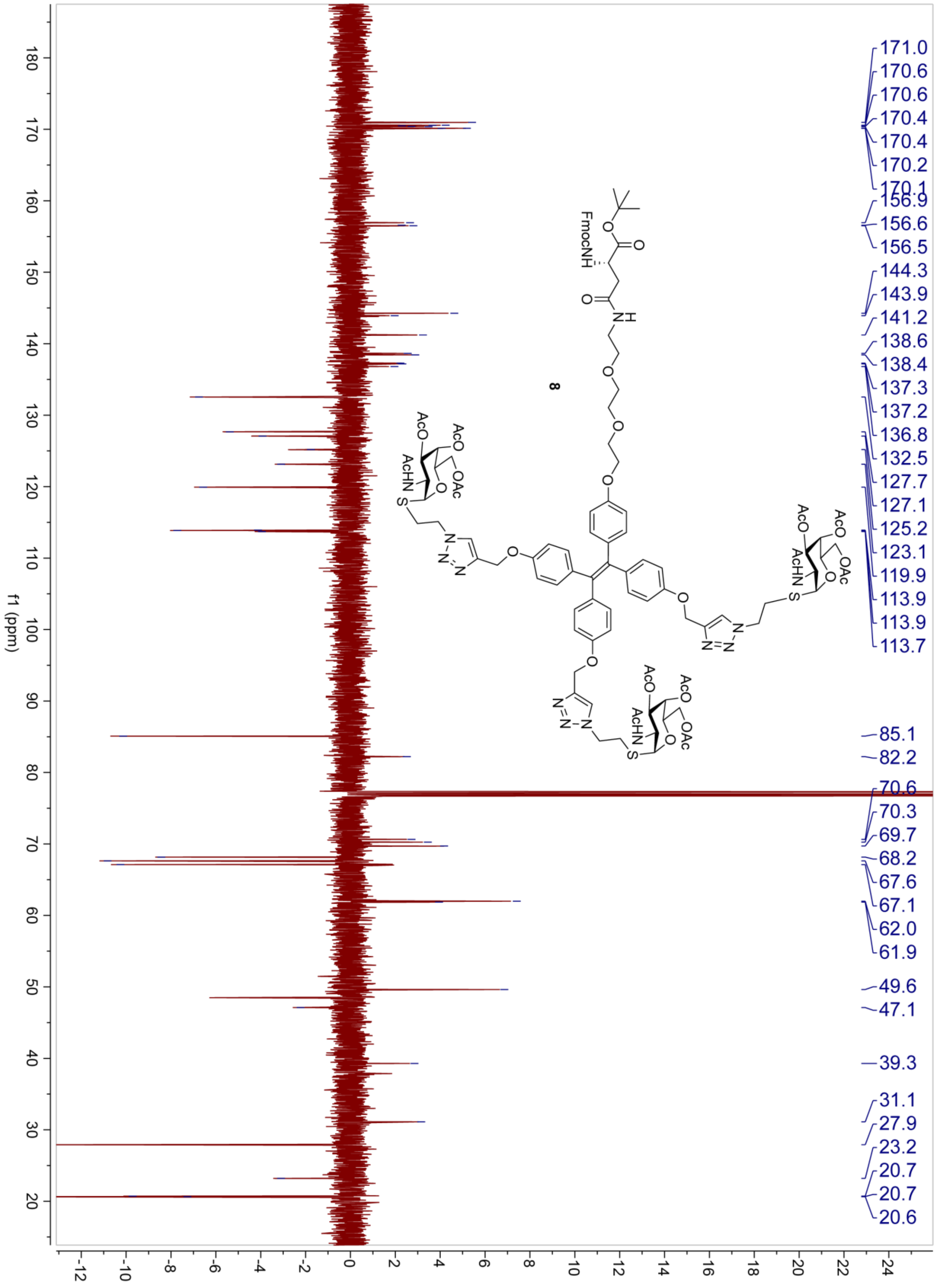

171.0
170.6
170.6
170.4
170.4
170.2
170.1
156.9
156.6
156.5
144.3
143.9
141.2
138.6
138.4
137.3
137.2
136.8
132.5
127.7
127.1
125.2
123.1
119.9
113.9
113.9
113.7
85.1
82.2
70.6
70.3
69.7
68.2
67.6
67.1
62.0
61.9
49.6
47.1
39.3
31.1
27.9
23.2
20.7
20.7
20.6
8
f1 (ppm)

TPEaa (1)

A solution of **8** (1.2 g, 0.53 mmol) in TFA-DCM-H_2O (48:48:4, 10 mL) was stirred at room temperature for 2h. The solvent was co-evaporated with toluene. Column chromatography (DCM-MeOH-AcOH 98:2:0.1→ 90:10:0.1, gradient elution) yielded the titled compound (1.15 g, 95%).

^{1}H NMR (600 MHz, chloroform-d) δ 7.82 – 7.75 (m, 2H, Fmoc protons), 7.76 – 7.70 (m, 3H, triazole), 7.58 (d, J = 6.4 Hz, 2H, Fmoc protons), 7.38 (t, J = 7.4 Hz, 2H, Fmoc protons), 7.28 (t, J = 7.3 Hz, 2H, Fmoc protons), 7.26 – 7.22 (m, 1H, Fmoc protons), 7.17 (d, J = 7.1 Hz, 1H, Fmoc protons), 6.97 – 6.85 (m, 8H, aromatic protons), 6.67 (s, 6H, aromatic protons), 6.61 (d, J = 8.4 Hz, 2H, aromatic protons), 6.54 (br s, 1H, NH), 6.20 (br s, 1H, NH), 5.70 (d, J = 4.8 Hz, 3H, H-1), 5.39 (s, 3H, H-4), 5.21 – 5.07 (m, 6H, CH_2), 5.01 (d, J = 11.7 Hz, 3H, H-3), 4.71 (dt, J = 11.8, 7.4 Hz, 3H, H-2), 4.66 – 4.56 (m, 6H, CH_2), 4.55 – 4.45 (overlapped protons, 4H, CH Asp and H-5), 4.39 – 4.28 (m, 2H, CH_2 Fmoc), 4.22 – 4.17 (m, 2H, CH Fmoc), 4.17 – 4.08 (m, 6H, H-6), 4.08 – 4.01 (m, 2H, CH_2 TEG), 3.86 – 3.79 (m, 2H, CH_2 TEG), 3.72 – 3.67 (m, 2H, CH_2 TEG), 3.66 – 3.58 (m, 2H, CH_2 TEG), 3.57 – 3.47 (m, 2H, CH_2 TEG), 3.39 – 3.30 (m, 2H, CH_2 TEG), 3.23 – 3.14 (m, 3H, CH_2), 3.14 – 3.04 (m, 3H, CH_2), 2.86 – 2.78 (m, 1H, CH_2 Asp), 2.83 - 2.79 (m, 1H, CH_2 Asp), 2.17 (d, J = 2.0 Hz, 9H, NHAc), 2.08 – 1.96 (m, 27H, OAc);

^{13}C NMR (150 MHz, chloroform-d) δ 173.2 (C), 171.2 (C), 170.8 (C), 170.4 (C), 159.2 (C), 159.0 (C), 156.7 (C), 156.2 (C), 143.6 (C), 141.2 (C), 138.7 (C), 138.5 (C), 137.4 (C), 132.6 (CH, aromatic), 129.0 (CH, Fmoc), 128.2 (CH, Fmoc), 127.8 (CH, Fmoc), 127.1 (CH, Fmoc), 125.3 (CH, Fmoc), 125.1 (CH, Fmoc), 120.0 (CH, triazole), 113.8 (CH, aromatic), 113.7 (CH, aromatic), 84.6 (CH,C-1), 70.7 (CH_2, TEG), 70.4 (CH_2, TEG), 70.0 (CH_2, TEG), 69.7 (CH_2, TEG), 69.0 (CH_2, TEG), 68.0 (CH, C-3), 67.6 (CH, C-5), 67.3 (CH_2, TEG), 66.9 (CH, C-4), 61.9 (CH_2, C-6), 61.0 (CH_2,) , 50.9 (CH, Asp), 50.2 (CH_2), 49.0 (CH, C-2), 46.9 (CH, Fmoc), 39.8 (CH_2, TEG), 37.8 (CH_2, Asp), 30.9 (CH_2), 22.6 (CH_3, NHAc), 22.6 (CH_3, NHAc), 22.5 (CH_3, NHAc), 20.6 (CH_3, OAc), 20.6 (CH_3, OAc), 20.6 (CH_3, OAc), 20.6 (CH_3, OAc);

ES-HRMS calcd for $C_{108}H_{125}N_{14}O_{35}S_3$ 2273.7594, found m/z2273.7592 $[M-H]^{-v}$;

[α]$_D$^{20} + 59 (c 0.13, $CHCl_3$).

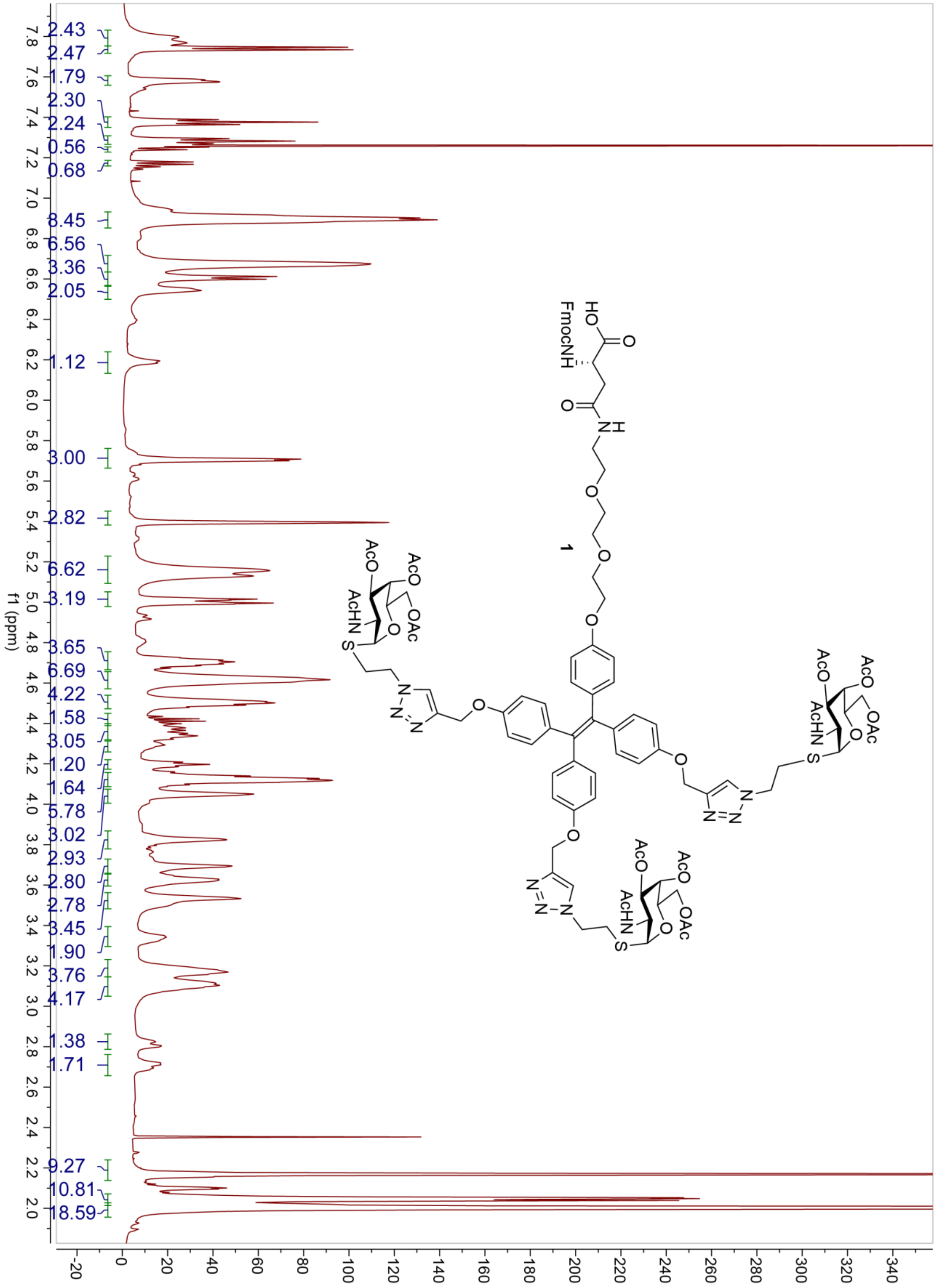
f1 (ppm)
1
HO
FmocNH
AcO
OAc
AcHN

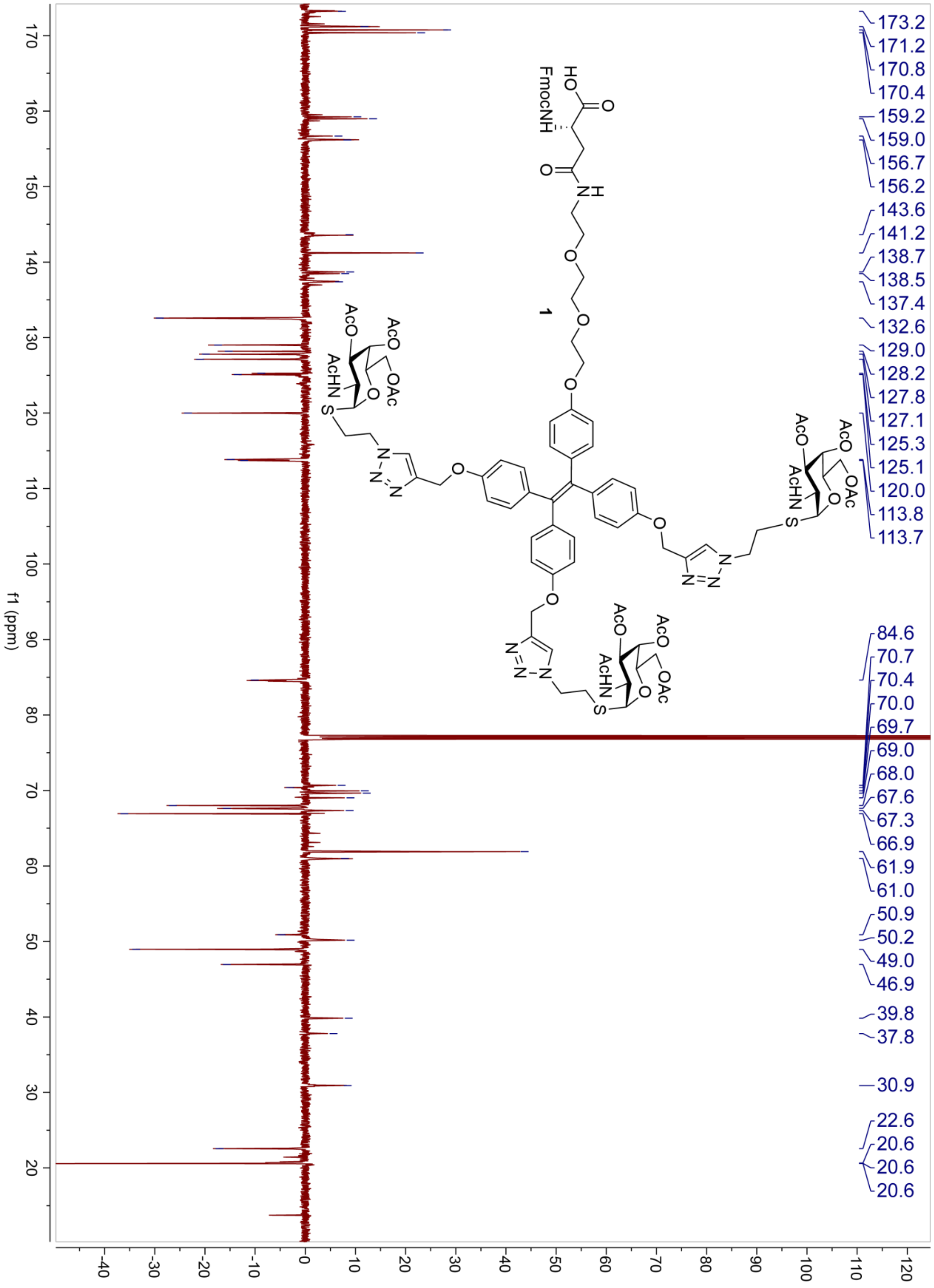
173.2
171.2
170.8
170.4
159.2
159.0
156.7
156.2
143.6
141.2
138.7
138.5
137.4
132.6
129.0
128.2
127.8
127.1
125.3
125.1
120.0
113.8
113.7
84.6
70.7
70.4
70.0
69.7
69.0
68.0
67.6
67.3
66.9
61.9
61.0
50.9
50.2
49.0
46.9
39.8
37.8
30.9
22.6
20.6
20.6
20.6
f1 (ppm)
1
FmocNH
HO
AcO
OAc
AcHN
S

3 Solid phase peptide synthesis and related purification: General conditions

Solid phase resins were purchased from Rapp Polymere GmbH, Tübingen. Protected amino acid building blocks for Fmoc-SPPS (Novabiochem) were purchased from Merck KGaA, Darmstadt and Merck Schuchardt OHG, Hohenbrunn. N,N-dimethylformamide, N-methypyrrolidone, trifluoroacetic acid and piperidine were purchased from Biosolve Chimie SARL, Dieuze, France. Coupling reagents HBTU and HATU (Novabiochem) were purchased from Merck KGaA, Darmstadt. HOBt hydrate was purchased from Sigma-Aldrich GmbH, Steinheim and recrystallized from absolute ethanol and dried at reduced pressure. HOAt was purchased from GL Biochem, Shanghai, China.

RP-HPLC: Analytic RP-HPLC was performed on a Dionex U-3000 (Thermo Scientific) system (DR-3600 six channel degasser, LPG-3x00 pump, TCC-3100 column compartment, DAD-3000 UV/VIS diode array detector, WPS-3000 autosampler). A Luna C18(2) (3 µm, 100Å, 150 x 2.0 mm) column from Phenomenex was applied for analytical HPLC. The flow rate was set to 1 mL/min. Preparative RP-HPLC was performed on a Dionex U-3000 system (HPG-3200P, VWD-3400 UV/VIS detector, AFC-3000 sampler). A Luna C18(2) (10 µm, 100Å, 250 x 21.2 mm) column from Phenomenex was applied for preparative HPLC. The flow rate was set to 20 mL/min. Eluent water-acetonitrile +0.1 % TFA.

(Glyco-)peptides were detected at a wavelength of 214 nm. Both systems were operated and chromatograms analyzed with Dionex Chromeleon (version 6.80DU10a Build 2826(171948)).

3.1 General protocol for automated glycopeptide-solid phase peptide synthesis

Peptides were synthesized on a Syro I automated peptide synthesizer (Multisyntech GmbH, Witten) using a 24 x 2 mL reactor plate. The synthesis scale is reported in the experimental section for individual peptides. Stock solutions of the relevant Fmoc-aa-OH 0.5M in DMF (Fmoc-Phe-OH 0.5 M in NMP), HBTU and HOBt 0.45 M in DMF, DIPEA 2M in NMP and 20 % piperidine in DMF were prepared. Preloaded TentaGel-Fmoc-aa-Trt was used, if not otherwise specified. The resin was manually swelled with 500 µL of DCM for 30 min and then washed with DMF for 5 times (500 µL, 40s vortex, 20s vac). The Fmoc-protecting group was initially cleaved using 20% piperidine in DMF (46.2 µL per 1 µmol batch size; 2 x 3 min + 1 x 9 min; 15s vortex, 45 s break), then the resin was washed with DMF (46.2 µL per 1 µmol batch size; 6 x 1 min, 15 s vortex; 45 s break). In automated reaction cycles the corresponding Fmoc-aa-OH (8 eq), HBTU (7.6 eq), HOBt (8 eq) and DIPEA (16 eq) were added to the reactor and mixed by vortexing for 40 min in 15 sec pulses with a 2.75 min interval. A reaction cycle was concluded by Fmoc deprotection before the next cycle was carried out. Glycosylated amino acids, spacer and **1** with the adopted coupling reagents were added manually into the synthesis reactors. The coupling condition for individual non-traditional amino acids are reported in below.

3.2 Manual coupling protocol for Glycosylated amino acids

HATU (1.4 eq), HOAt (1.4 eq) and DIPEA (2.8 eq) were added to a solution of glycosylated amino acids (T* and S*) in DMF (1.5 eq in 200µL). The solution was quickly added to the reactor and mixed by vortex for 15s vortex; 2.45 min break) at room temperature for 8h. After the reaction, the resin was washed with DMF for 5 times (500 µL, 40s vortex, 20s vac). The Fmoc group was cleaved by triple addition of 500 µL each of 20 vol% piperidine in DMF (3 x 3 x 9 min; 15s vortex, 45 s break) and the resin was washed with DMF (5 x 1 min 6 x 500 µL, 15 s vortex; 45 s break).

3.3 Spacer manual coupling protocol

HBTU (3 eq) , HOBT (3 eq) and DIPEA (6 eq) were added to a solution of 3-(2-(2-(2-aminoethoxy)ethoxy)ethoxy)propanoic acid in DMF (3 eq in 200µL). The solution was quickly added to the reactor and mixed by vortexing in 15 sec pulses followed by 2.45 min intervals at room temperature for 3hrs. After the reaction, the resin was washed with DMF for 5 times (500 µL, 40 s vortex, 20 s vac). The Fmoc-group was cleaved by triple addition of 500 µL each of 40

vol% piperidine in DMF (3 x 3 x 9 min; 15s vortex, 45 s break) and the resin was washed with DMF (5 x 1 min 6 x 500 µL, 15 s vortex; 45 s break).

3.4 Biotinylated lysine manual coupling protocol

HBTU (3 eq), HOBT (3 eq) and DIPEA (6 eq) were added to a solution of Fmoc-Lys(Biotin)-OH (aapptec) in NMP (3 eq in 200µL). The solution was quickly added to the reactor and mixed by vortexing in 15 sec pulses with 2.45 min intervals for 3h at room temperature. After the reaction, the resin was washed with DMF for 5 times (500 µL, 40s vortex, 20s vac). Then, the Fmoc-group was cleaved by triple addition of 500 µL each of 40 vol% piperidine in DMF (3 x 3 x 9 min; 15s vortex, 45 s break) and the resin washed with DMF (5 x 1 min 6 x 500 µL, 15 s vortex; 45 s break).

3.5 Manual coupling protocol

HATU (1.8 eq), HOAt (1.8 eq) and DIPEA (3.6 eq) were added to a solution of **1** in DMF (2 eq. in 200µL). The solution was quickly added to the reactor and shaken by vortex (15 s vortex; 2.45 min break) at room temperature for 8h. After the reaction, the resin was washed with DMF for 5 times (500 µL, 40s vortex, 20s vac). The the Fmoc-group was cleaved by triple addition of 500 µL each of 20 vol% piperidine in DMF (3 x 3 x 9 min; 15s vortex, 45 s break) and the resin washed with DMF (5 x 1 min 6 x 500 µL, 15 s vortex; 45 s break).

3.6 Release of the peptides from the solid phase resin protocol

After synthesis, the resin was washed with dichloromethane, isopropanol and diethylether (5 x 500 µL each; 5 x 1 min; 15 s vortex; 45 s break), dried in an airstream for 30 min and then transferred from the reactor to a 2 mL syringe containing a fritt. The glycopeptides were cleaved from the resin by three additions of TFA/TIPS/H_2O 15:0.9:0.9 (1 x 120 min + 2 x 10 min; 15 s vortex; 2.45 min break). The combined filtrates were co-evaporated with toluene. The glycosylated peptide was dissolved in 5ml of water and loaded on a prepacked (MeOH 5mL x 5, H_2O 5 mL x 5) C18-column (Waters Sep-Pak Vac 6cc (1 g)). The column was eluted with 5 x 5ml of water and 7 x 5ml of CH_3CN/H_2O 70:30 solution. Fractions containing acetonitrile were combined and the organic solvent was removed by vacuum. The aqueous residue was lyophilized to give the crude glycopeptide product.

3.7 Removal of the carbohydrate acetyl protecting groups protocol

The crude glycopeptides were dissolved in 5-10 mL methanol and were treated with 1% sodium methoxide in methanol until a pH of 9.5 was reached (wet pH paper). The reaction was stirred at room temperature and followed by analytical HPLC and MALDI-TOF (16-24 h).

The reaction mixture was neutralized with acetic acid and the solvent removed in vacuo. The crude glycopeptide was purified by preparative HPLC and lyophilized from water to give the product as a colourless lyophilizate foam.

3.8 Synthesis yields and analytical data for peptides 9 to 14

Peptide **9**, Sequence: Biotin-FNNFTVSFWLRVPKVSASHLE

Yield: 39% (13.26 mg, 4.90 µmol). Analytical HPLC Rt= 52.10 min (Phenomenex Luna C18 (2), 2.0 x 150 mm, 3 µm, Grad: eluent A/B + 0.1% TFA (5:95)→(60:40), 5-60 min, wavelength=214 nm). Semi-preparative HPLC Rt= 32.57 min (GLSciences Inc, InertSustain C18, 5 µm, 6.0x250mm, Grad: eluent A/B + 0.1 % TFA (20:80)→(50:50), 5-35min, wavelength=214 nm); *HR-ESI-MS* (Thermo LTQ Orbitrap XL™ hybride FT), *m/z*: 1353.1920 ($[M+2H]^{2+}$, calc. 1353.1911), 902.4638 ($[M+3H]^{3+}$, calc. 902.4633).

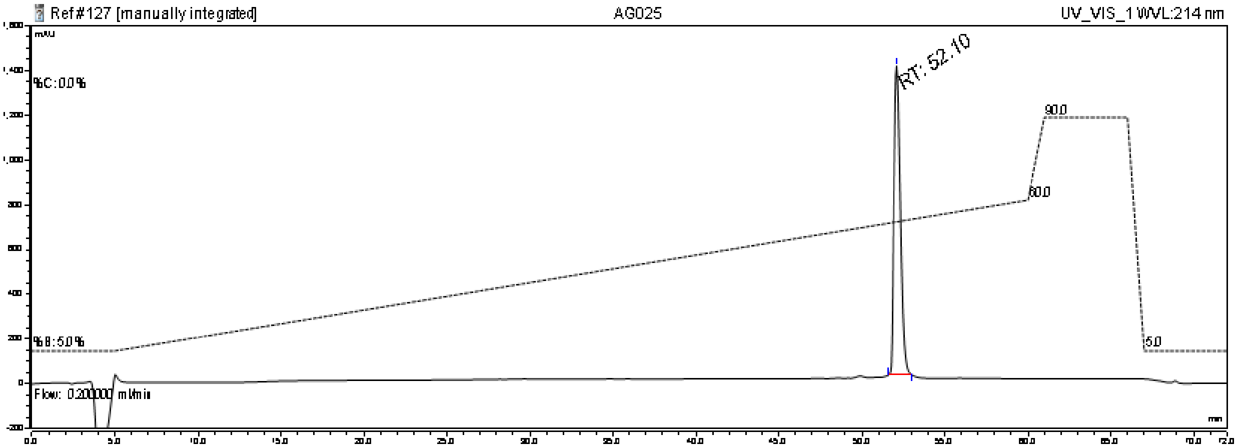

Peptide **10**, sequence: NH_2-HGVTSAPDTRPAPGSTAPK(Biotin)A

Yield: 48% (7.77 mg, 3.62 µmol). Analytical HPLC: Rt= 28.48 min (Phenomenex Luna C18 (2), 2.0 x 150 mm, 3 µm, Grad: eluent A/B 0.1% TFA (5:95)→(60:40), 5-60 min, wavelength=214 nm). Preparative: HPLC Rt= 19.46 min (GLSciences Inc, InertSustain C18, 5 µm, 6.0x250mm, Grad: eluent A/B + 0.1 % TFA (15:85)→(35:65), 5-85 min, wavelength=214 nm); *HR-ESI-MS* (Thermo LTQ Orbitrap XL hybride FT), *m/z*: 695.6739 ($[M+3H]^{3+}$, calc. 695.6739), *HR-ESI-MS* (Thermo LTQ Orbitrap XL hybride FT), *m/z*: 1073.0344 ($[M+2H]^{2+}$, calc. 1073.0335), 715.6912 ($[M+3H]^{3+}$, calc. 715.6916).

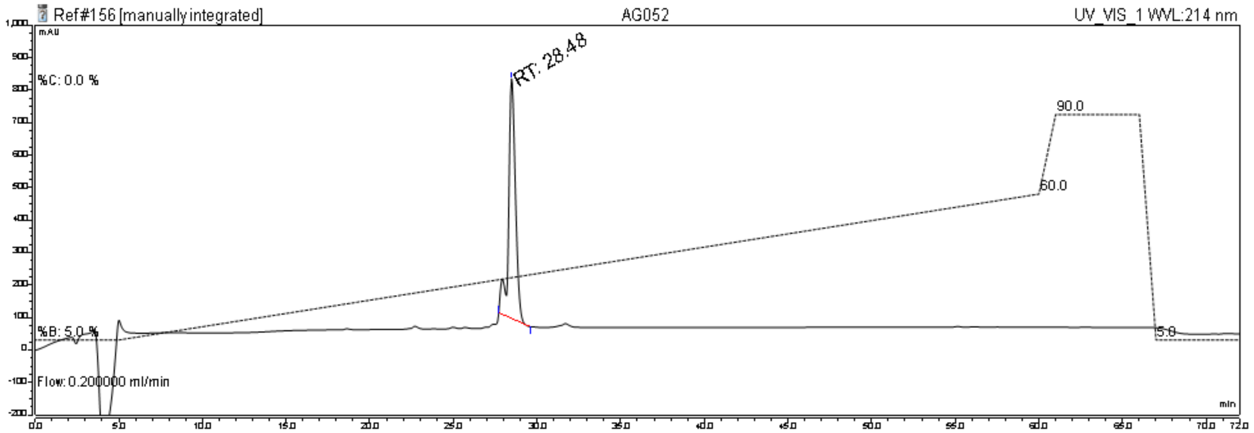

Peptide **11**, Sequence: NH_2-HGVTSAPDT*RPAPGS*T*APK(Biotin)A, *=GalNAc

Yield: 38% (13.1 mg, 4.76 µmol). Analytical HPLC Rt= 26.31 min (Phenomenex Luna C18 (2), 2.0 x 150 mm, 3 µm, Grad: eluent A/B + 0.1% TFA (5:95)→(60:40), 5-60 min, wavelength=214 nm). Preparative HPLC Rt= 33.83 min (GLSciences Inc, InertSustain C18, 5 µm, 6.0x250mm, Grad: eluent A/B + 0.1 % TFA (10:90)→(35:65), 5-105 min, wavelength=214 nm); *HR-ESI-MS* (Thermo LTQ Orbitrap XL hybride FT), *m/z*: 1377.6538 ($[M+2H]^{2+}$, calc. 1377.6526), 918.7718 ($[M+3H]^{3+}$, calc. 918.7710).

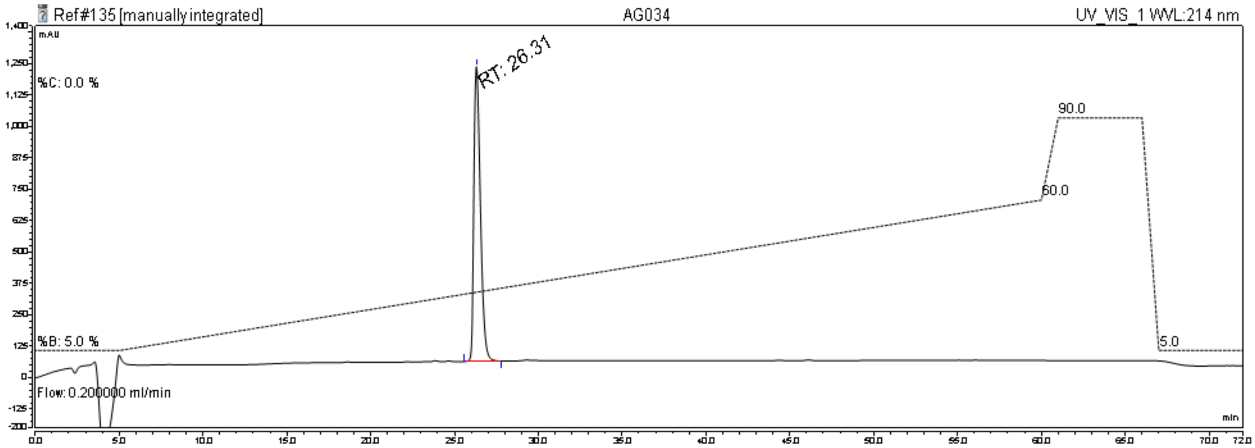

Peptide **12**, Sequence: NH_2-**TPEaa**-HGVTSAPDT*RPAPGS*T*APK(Biotin)A, *=GalNAc

Yield: 20% (6.62 mg, 1.50 µmol). Analytical HPLC Rt= 40.83 min (Phenomenex Luna C18 (2), 2.0 x 150 mm, 3 µm, Grad: eluent A/B + 0.1% TFA (5:95)→(60:40), 5-60 min, wavelength=214 nm). Preparative HPLC Rt= 47.15 min (GLSciences Inc, InertSustain C18, 5 µm, 6.0x250mm, Grad: eluent A/B + 0.1 % TFA (20:80)→(55:45), 5-145min, wavelength=214 nm); *HR-ESI-MS*

(Thermo LTQ Orbitrap XL hybride FT), *m/z*: 1471.3036 ($[M+3H]^{3+}$, calc. 1471.3033), 1103.7301 ($[M+4H]^{4+}$, calc. 1103.7294).

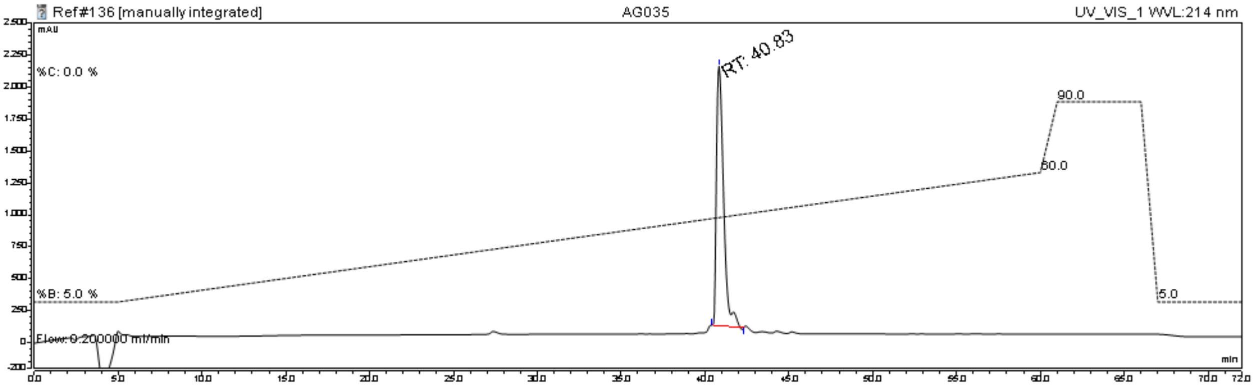

Peptide **13**, Sequence: NH_2- **TPEaa** -HGVTSAPDTRPAPGSTAPK(Biotin)A

Yield: 29% (8.33 mg, 2.19 µmol). Analytical HPLC Rt= 41.62 min (Phenomenex Luna C18 (2), 2.0 x 150 mm, 3 µm, Grad: eluent A/B + 0.1% TFA (5:95)→(60:40), 5-60 min, wavelength=214 nm). Preparative HPLC Rt= 51.23 min (GLSciences Inc, InertSustain C18, 5 µm, 6.0x250mm, Grad: eluent A/B + 0.1 % TFA (20:80)→(55:45), 5-145min, wavelength=214 nm); *HR-ESI-MS* (Thermo LTQ Orbitrap XL hybride FT), *m/z*: 1901.9331 ($[M+2H]^{2+}$, calc. 1901.8319), 1268.2244 ($[M+3H]^{3+}$, calc. 1268.2239).

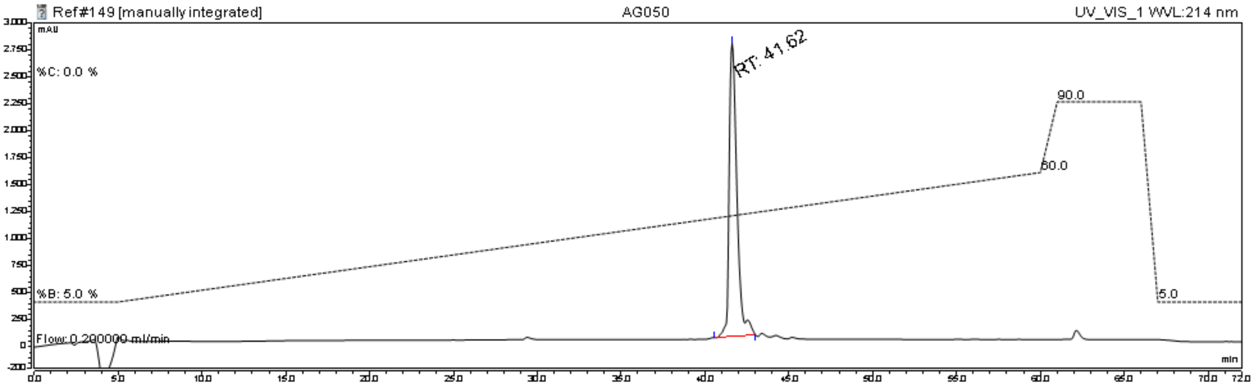

Peptide **14**, Sequence: NH_2- **TPEaa** -Spacer-HGVTSAPDTRPAPGSTAPK(Biotin)A

Yield: 31% (9.36 mg, 2.34 µmol). Analytical HPLC Rt= 41.79 min (Phenomenex Luna C18 (2), 2.0 x 150 mm, 3 µm, Grad: eluent A/B + 0.1% TFA (5:95)→(60:40), 5-60 min, wavelength=214 nm). Preparative HPLC Rt= 31.63 min (GLSciences Inc, InertSustain C18, 5 µm, 6.0x250mm, Grad: eluent A/B + 0.1 % TFA (25:75)→(60:44), 5-145min, wavelength=214 nm); *HR-ESI-MS*

(Thermo LTQ Orbitrap XL hybride FT), *m/z*: 1335.9294 ($[M+3H]^{3+}$, calc. 1335.9291), 1002.1995 ($[M+4H]^{4+}$, calc. 1002.1988).

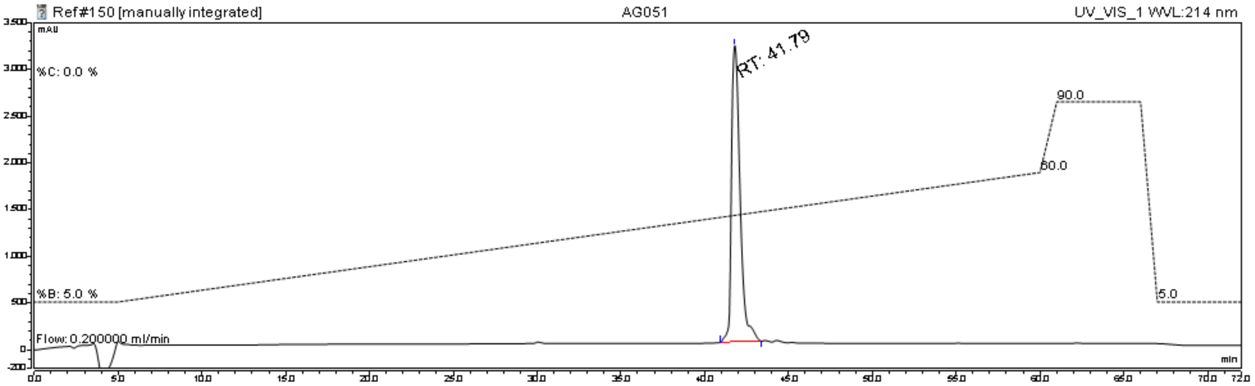

Peptide **15**, Sequence: NH_2-FNNFTVSFWLRVPKVSASHLE-Spacer-HGVTSAPDT*RPAPGS*T*APPA, *=GalNAc

Yield: 6% (3.59 mg, 0.70 μmol). Analytical HPLC Rt= 44.07 min (Phenomenex Luna C18 (2), 2.0 x 150 mm, 3 μm, Grad: eluent A/B + 0.1% TFA (5:95)→(60:40), 5-60 min, wavelength=214 nm). Preparative HPLC Rt= 59.80 min (GLSciences Inc, InertSustain C18, 5 μm, 6.0x250mm, Grad: eluent A/B + 0.1 % TFA (20:80)→(50:50), 5-125min, wavelength=214 nm); *HR-ESI-MS* (Thermo LTQ Orbitrap XL hybride FT), *m/z*: 1290.9000 ($[M+4H]^{4+}$, calc. 1290.8988), 1032.9216 ($[M+5H]^{5+}$, calc. 1032.9206), 860.9352 ($[M+6H]^{6+}$, calc. 860.9351).

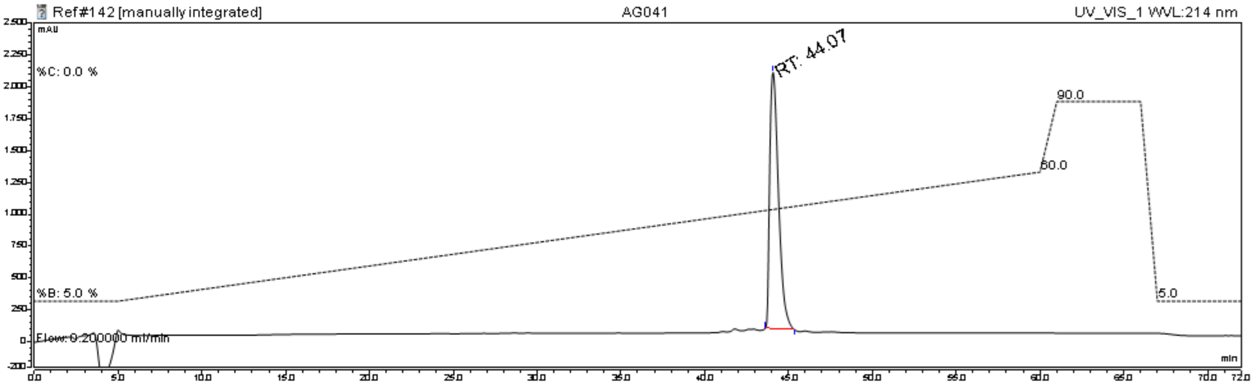

Peptide **16**, Sequence: NH_2- **TPEaa** -FNNFTVSFWLRVPKVSASHLE-Spacer-HGVTSAPDT*RPAPGS*T*APPA, *=GalNAc

Yield: 1% (1.01 mg, 0.15 μmol). Analytical HPLC Rt= 47.48 min (Phenomenex Luna C18 (2), 2.0 x 150 mm, 3 μm, Grad: eluent A/B + 0.1% TFA (5:95)→(60:40), 5-60 min, wavelength=214 nm). Preparative HPLC Rt= 74.47 min (GLSciences Inc, InertSustain C18, 5 μm, 6.0x250mm,

Grad: eluent A/B + 0.1 % TFA (20:80)→(55:45), 5-145min, wavelength=214 nm); *HR-ESI-MS* (Thermo LTQ Orbitrap XL hybride FT), *m/z*: 1364.4409 ($[M+5H]^{5+}$, calc. 1364.4399), 1137.2017 ($[M+6H]^{6+}$, calc. 1137.2013).

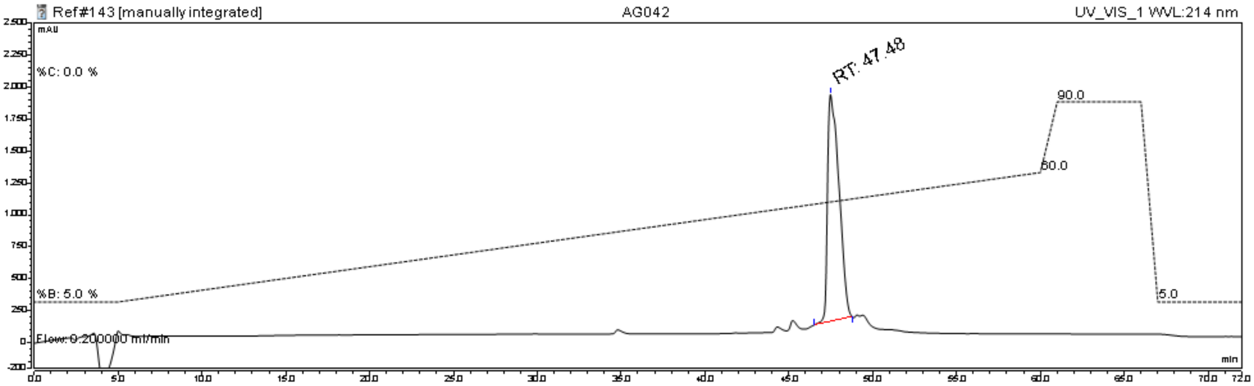

3.9 Synthesis yields and analytical data for peptides M96 to M98

Peptide **M96**: MUC5B(13mer) T-antigen: H_2N-(TEG)-AT*PSST*PGTTHTP-OH

Yied: 62% (17.7 mg, 8.1 μmol). Analytical HPLC R_t = 14.22 min (A/B: (95:5) → (65:35), 200 μL/min, 30 min); Preparative HPLC R_t = 12.86 min (A/B: (95:5) → (70:30), 20 mL/min, 25 min); *HR-ESI-MS*, *m/z*: 1094.9930 ($[M+2H]^{2+}$, calc. 1094.9934), 911.9259 ([M-{Galβ(1,4)-GalNac}+2H]$^{2+}$, calc. 911.9353), 366.1395 ([Galβ(1,4)-GalNac]$^{+}$, calc. 366.1395).

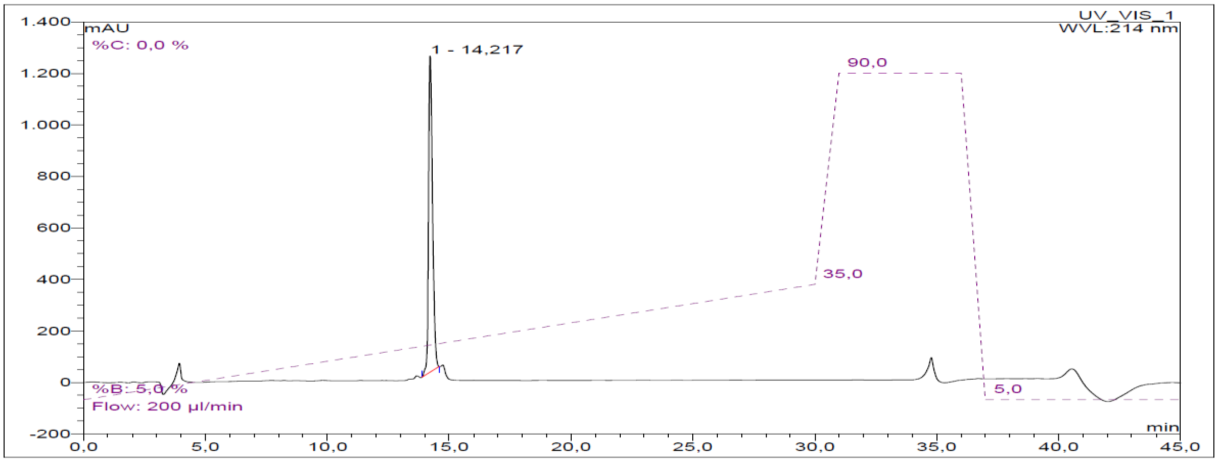

Peptide **M97**: MUC5B(13mer) T-antigen: H_2N-(TEG)-AT*PSSTPGT*THTP-OH

Yield: 79% (22.3 mg, 10.2 µmol).Analytical HPLC R_t = 14.24 min (A/B: (95:5) → (65:35), 200 µL/min, 30 min); Preparative HPLC R_t = 12.63 min (A/B: (95:5) → (70:30), 20 mL/min, 25 min); *HR-ESI-MS*, *m/z*: 1094.9937 ($[M+2H]^{2+}$, calc. 1094.9934), 730.3307 ($[M+3H]^{3+}$, calc. 730.3314), 557.4867 ($[M+K+3H]^{4+}$, calc. 557.4893).

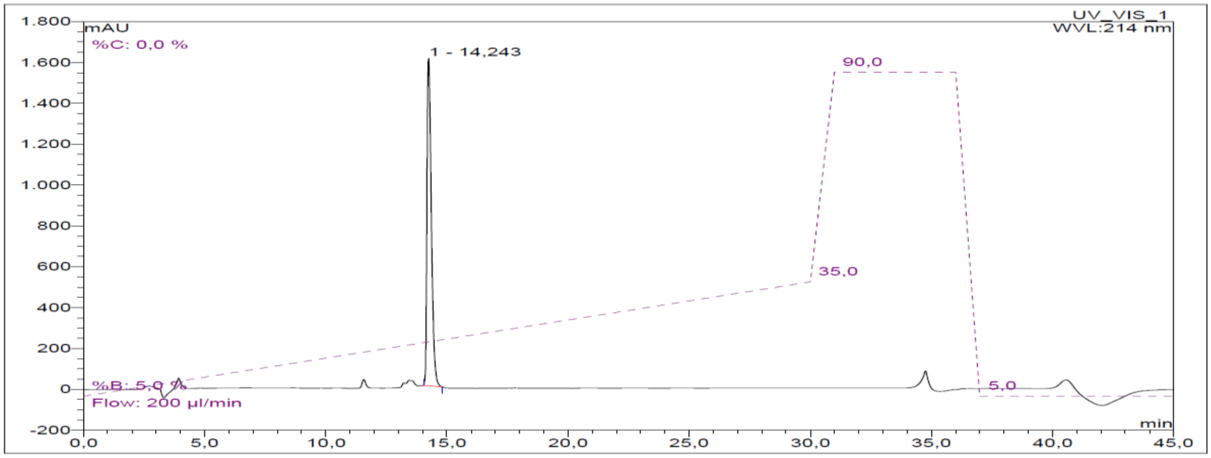

Peptide **M98**: MUC5B(13mer) T-antigen: H_2N-(TEG)-AT*PSST*PGT*THTP-OH

Yield: 61% (20.2 mg, 8.1 µmol).Analytical HPLC R_t = 13.79 min (A/B: (95:5) → (65:35), 200 µL/min, 30 min); Preparative HPLC R_t = 13.82 min (A/B: (95:5) → (70:30), 20 mL/min, 25 min); *HR-ESI-MS*, *m/z*: 1277.5626 ($[M+2H]^{2+}$, calc. 1277.5595), 852.0418 ($[M+3H]^{3+}$, calc. 852.0421), 648.7709 ($[M+K+3H]^{4+}$, calc. 648.7724).

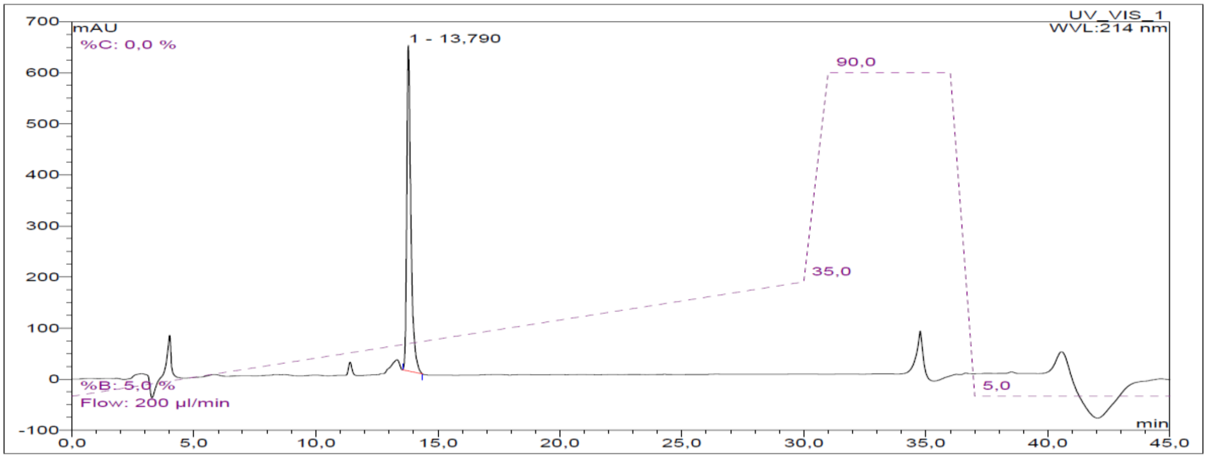

4 *In vitro* & *in cellulo* peptide uptake study

4.1 Biotinylated MUC1 peptides/MGL-Fc ELISA protocol

The optimal peptides/protein concentration was determined by a pilot titration experiment using biotinylated Polyacrylamide coupled-GalNAc polymer (PAA-GalNAc), (Lectinity), Figure S7.

Streptavidin-coated plates (Thermo Scientific, cat. no. 15125) were washed 3 times with TSM (20 mM Tris-HCl, pH 7.4, 150 mM NaCl, 2 mM $MgCl_2$, 1 mM $CaCl_2$) followed by 2 h incubation at room temperature with the biotinylated peptides in a 2x dilution series starting from 1 μM in assay buffer (0.5% BSA in TSM). The plates were washed 3 times with TSM and MGL-Fc 0.5 μg/ml from a house stock of 1.8 mg/ml in assay buffer was added and incubated for 2 h at room temperature. The plates were then washed 3 times with TSM / 0.05% Tween-20.

Goat-anti-human-PO (Jackson ImmunoResearch, cat. no. 109-036-098) diluted 1:1500 in TSM/0.05 % Tween-20 was added and incubated for 30 min at room temperature. The plates were then washed 6 times with TSM/0.05% Tween-20.

100 μl of substrate solution was added to each well until positive wells turn bright blue at which point 50 μl/well of stop solution was added. Substrate-buffer was prepared by dissolving 21.02 g of citric acid (Merck 244, $C_6H_8O_7 \cdot H_2O$) and 8.2 g of sodium acetate (J.T Baker, 0258, CH_3COONa) in 990 ml of deionized water and adjusting to pH 4.0 with acetic acid (Riedel de Haen, 33209) and making up to 1 L.

Substrate solution was prepared by adding 0.1 mL of 10 mg/ml TMB and 1μl 30% of H_2O_2 in 10ml substrate-buffer. This solution was used immediately. The stop solution was 0.8 M H_2SO_4. Absorption was measured at 450 nm.

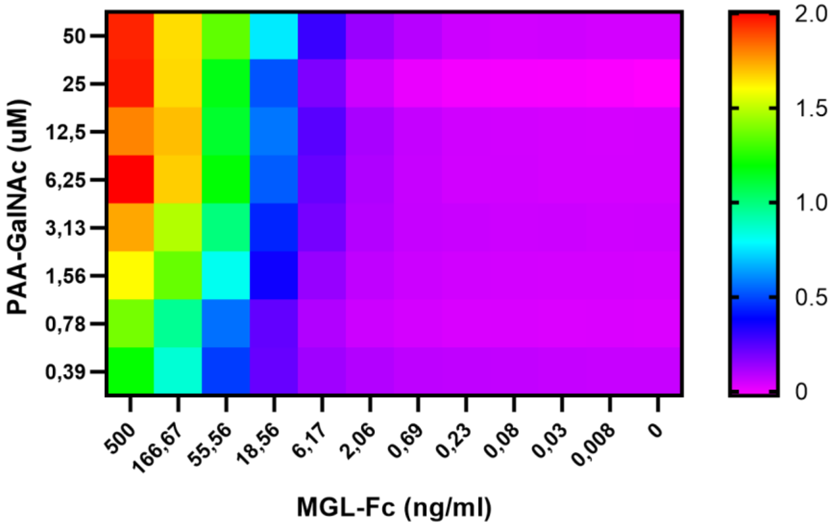

Figure S7. Heatmap of absorbance at 450nm derived from MGl-Fc and Biotin-Polyacrylamide coupled-GalNAc polymer (PAA-GalNAC) ELISA assay.

4.2 Murine bone marrow derived dendritic cell generation

Femur and tibia of the hind legs of C57BL/6J mice were sterilely prepared and the bone marrow was rinsed out with MEM using a 10 ml syringe with a 0.55 mm x 25 mm needle. The isolated bone marrow was resuspended with a pipette several times and then centrifuged for 10 min at 400 g. To prevent rupture of erythrocytes, cells were lysed with 1 ml mouse Geys lysis buffer. The reaction was stopped after 1 min by adding 4 mL of MEM supplemented with 2% FCS. The cell suspension was filtered using a 0.45 µm filter and re-centrifuged. The supernatant was discarded and the cells were counted after resuspending in fresh Iscoves media supplemented with 10% FCS, 2mM glutamine and 1mM sodium pyruvate. 5 x 10^6 cells were cultivated in 5 mls per well of a 6-well cell culture plate under dentritic cell (DC)-inducing conditions in order to bone marrow-derived DC (BMDCs) differentiation by adding 50 ng / mL GM-CFS into the growth medium. On day 3, the cells were fed with fresh medium. On day 6 DCs become non-adherent cells, the supernatant was removed and cultivated in a new 6 well culture plate with fresh medium. Cells were ready to use on day 7 - 9.

4.3 Flow cytometry uptake

20 µM Peptides were pre-incubated with Streptavidin (SA)-PE and then incubated with day 7 BMDCs cells in PBS buffer containing1mM $CaCl_2$ and 0.5mM $MgCl_2$ at 4°C or 37°C for 1h.

Wells were washed 2 times and stained for 30 mins at 4°C. Anti-CD11c-BV650 (Biolegend), MHCII-APC (eBiosience); fixable viability dye eFlour780 (eBiosience).

4.4 Microscopy

Day 6 BMDCs cells were seeded in a 96-well plate (150.000 cells per well) and were cultivated for 16h. The medium was removed and 5 µg/ml peptide was added in binding buffer (0.5% BSA in PBS supplemented with 2.5 mM $CaCl_2$ and 0.33mM $MgCl_2$) and incubated on ice for 1h. Cells were washed 2 times with the binding buffer and incubated with 5 µg/ml of Streptavidin-Alexa 594 in binding buffer for 30min on ice. The buffer was replaced with growth medium. The 0 h timepoint was imaged and the 1h sample incubated for another 1h at 37°C before imaging (Figure S8).

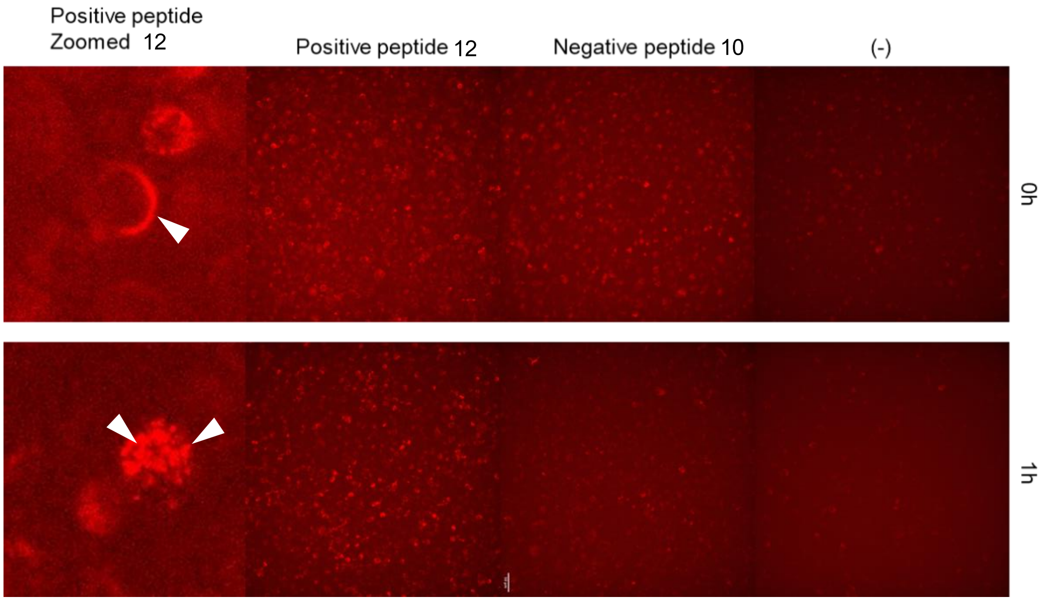

Figure S8. Binding and internalization of peptides **10** and **12** to BMDCs. Peptide **12** bind to the cells' suface at 0 °C and is internalized after 1h incubation at 37°C. No binding or internalization is observed with peptide **10**.

5 Post immunization and sera analysis

5.1 Generation of BSA-(11)$_n$ for ELISA plate coating

The glycosylated peptide where obtained following the procedure reported in previous paper.

Glycosylated peptide PEP1

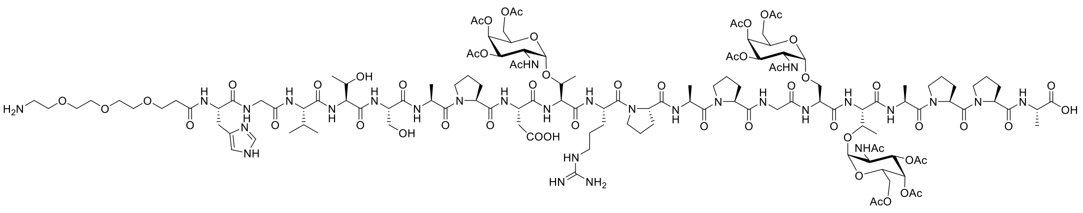

Figure S9. Chemical structure of the glycosylated peptide **PEP1**

The protected glycopeptide **PEP1** (Figure S9) was assembled using solid phase peptide synthesis following Fmoc-protocols with coupling using a HBTU-HOBt system. Glycosyated amino acids were introduced by the application of suitably protected glycosylated Fmoc-protected amino acids using HATU-HOAt as coupling conditions.

MALDI-ToF-MS: *m/z*: 3078.65 ($[M+H]^+$, calc. 3077.38).

Deprotected glycopeptide PEP2

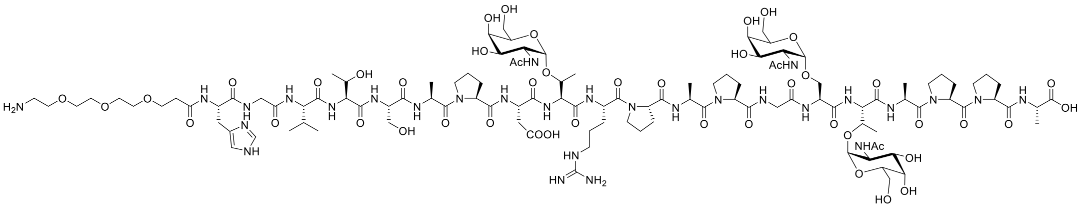

Figure S10. Chemical structure of the glycosylated peptide **PEP2**

The protected glycopeptide **PEP1** (52 mg, 17 mmol) was dissolved in aqueous NaOH (40 mL, pH 11.0) and stirred for 5 d until no more change in pH was observed. During the reaction time, the pH was readjusted to 11.0 from time to time. Thereafter, the mixture was neutralized by the addition of AcOH, followed by lyophilization and semipreparative HPLC to yield the deprotected glycopeptide **PEP2** (20 mg, 7.4 mmol, 44%) (Figure S10).

MALDI-ToF-MS: *m/z*: 2700.03 ($[M+H]^+$, calc. 2699.29).

Glycopeptide-squaric acid-conjugate PEP3

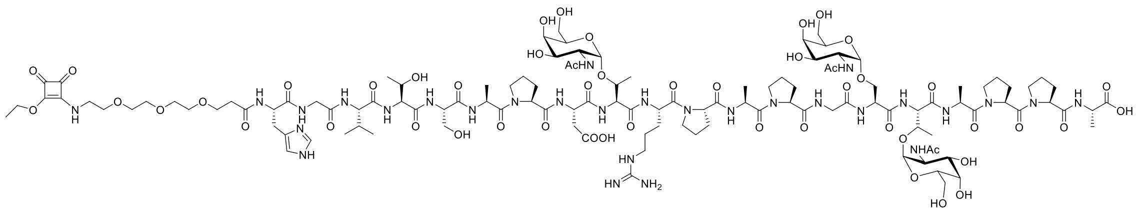

Figure S11. Chemical structure of the glycosylated peptide **PEP3**

The glycopeptide **PEP2** (20 mg, 7.4 µmol, 1.0 eq.) equipped with an amine terminated spacer was dissolved in a mixture of EtOH and H_2O (1:1 v/v, 2 mL). 3,4-Diethoxy-3-cyclobutene-1,2-dione (1.39 mg, 1.21 µL, 8.5 µmol, 1.15 eq.) were added and the pH was adjusted to 8.1 using a saturated aqueous Na_2CO_3. The reaction mixture was agitated for 4 h at room temperature and subsequently neutralized by addition of AcOH. The solvent was removed under reduced pressure and the crude product was subjected to semipreparative RP-HPLC (Phenomenex Luna C18 (2), 10 µm; 18.9 mL/min; H_2O-MeCN95:5 (0 min) → 60: 40 (35 min) → 0:100 (45 min)). Lyophilization of the product containing fractions yielded the squaric acid conjugate **PEP3** as colorless amorphous solid (12.4 mg, 4.4 µmol, 59%) (Figure S11).

MALDI-ToF-MS: *m/z*: 2823.25 ($[M+H]^+$, calc. 2823.31).

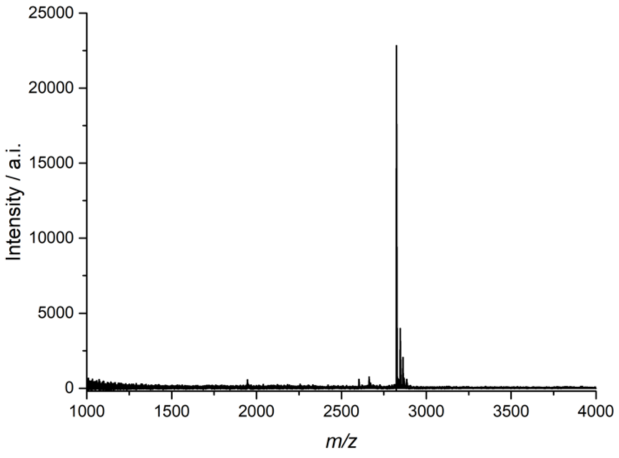

Glycopeptide-BSA-conjugate

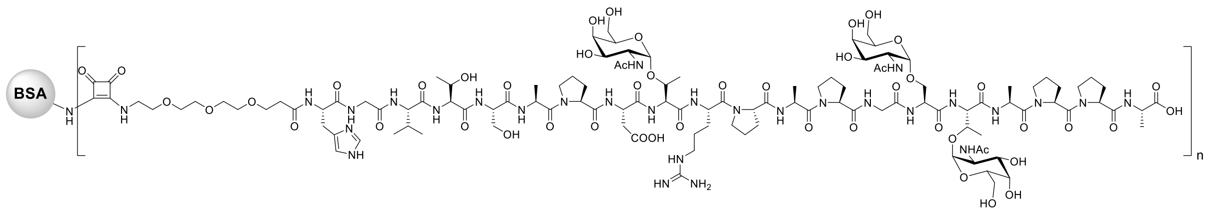

Figure S12. Chemical structure of the glycosylated peptide **PEP3**

Bovine serum albumin (BSA, 1.45 mg, 22 nmol, 1.0 eq.) and glycopeptide-squaric acid conjugate **PEP3** (1.85 mg, 654 nmol, 30 eq.) were dissolved in sodium hydrogenphosphate buffer (450 mM, pH 9.5, 1.5 mL). The reaction mixture was agitated for 3d at room temperature. Subsequently, the solution was filtrated using a ultrafiltration device equipped with a 30 kDa membrane to remove salts and free glycopeptide. The remainder was washed with deionized water (3 × 50 mL) until the pH of the filtrate was neutral. The supernatant was finally subjected to lyophilization to afford the BSA-glycopeptide conjugate (2.38 mg; the medium degree of functionalization determined by MALDI-ToF is 10 glycopeptides/BSA) (Figure S12).

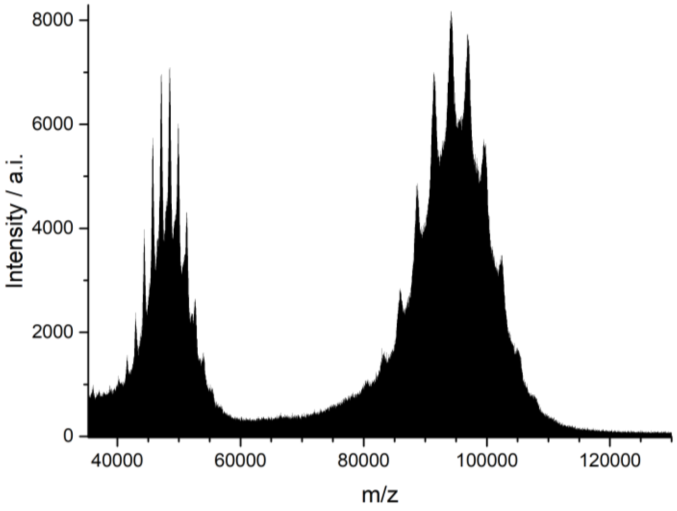

MALDI-TOF spectrum of glycopeptide-BSA-conjugate.

6 Microarray fabrication and binding studies

6.1 General spotting conditions

Glycopeptides were printed in 50 µM concentration in printing buffer (150 mM NaH_2PO_4/Na_2HPO_4, pH 8.5) in replicates of eight (150 pL per feature, 518 features per subarray and 8 subarrays per slide) on NHS-activated hydrogel slides (Nexterion slide H, Schott, Mainz, Germany) using a non-contact piezoelectric spotting device (iONE, M2 automation, Berlin, Germany) at a relative humidity of 55 %. The unreacted NHS-groups were blocked with 25 mM ethanolamine in 100 mM sodium tetraborate buffer (pH 9.0) for 1 h at room temperature. The full list of spotted glycopeptides on the microarray, table S2 and the core glycan structure, figure S13, are reported below.

Peptides M1-M87 were synthesised as previously reported.[4]
Peptides M88-M94 were synthesised as previously reported.[5]
Peptides M96-M98 were synthesised as reported in section 3.9 of the supporting information.

Table S2. Glycopeptides printed on the microarray. ID = Glycopeptide Microarray ID.

ID	Glycopeptide sequence MUC1	ID	Glycopeptide sequence
M1	PAHGV**T*(Tn)**SAPDTRPAPGSTA	M45	PAHGVTSAPDTRPAPGS**T*(C2T1$_{Hex}$)**A
M2	PAHGVTSAPD**T*(Tn)**RPAPGSTA	M46	PAHGV**T*(C2T1$_{Hex}$)**SAPD**T*(C2T1$_{Hex}$)**RPAPGSTA
M3	PAHGVTSAPDTRPAPGS**T*(Tn)**A	M47	PAHGV**T*(C2T1$_{Hex}$)**SAPDTRPAPGS**T*(C2T1$_{Hex}$)**A
M4	PAHGV**T*(Tn)**SAPD**T*(Tn)**RPAPGSTA	M48	PAHGVTSAPD**T*(C2T1$_{Hex}$)**RPAPGS**T*(C2T1$_{Hex}$)**A
M5	PAHGV**T*(Tn)**SAPDTRPAPGS**T*(Tn)**A	M49	PAHGV**T*(C2T1$_{Hex}$)**SAPD**T*(C2T1$_{Hex}$)**RPAPGS**T*(C2T1$_{Hex}$)**A
M6	PAHGVTSAPD**T*(Tn)**RPAPGS**T*(Tn)**A	M50	PAHGV**T*(C2T2$_{Hex}$)**SAPDTRPAPGSTA
M7	PAHGV**T*(Tn)**SAPD**T*(Tn)**RPAPGS**T*(Tn)**A	M51	PAHGVTSAPD**T*(C2T2$_{Hex}$)**RPAPGSTA
M8	PAHGV**T*(T)**SAPDTRPAPGSTA	M52	PAHGVTSAPDTRPAPGS**T*(C2T2$_{Hex}$)**A
M9	PAHGVTSAPD**T*(T)**RPAPGSTA	M53	PAHGV**T*(C2T2$_{Hex}$)**SAPD**T*(C2T2$_{Hex}$)**RPAPGSTA
M10	PAHGVTSAPDTRPAPGS**T*(T)**A	M54	PAHGV**T*(C2T2$_{Hex}$)**SAPDTRPAPGS**T*(C2T2$_{Hex}$)**A
M11	PAHGV**T*(T)**SAPD**T*(T)**RPAPGSTA	M55	PAHGVTSAPD**T*(C2T2$_{Hex}$)**RPAPGS**T*(C2T2$_{Hex}$)**A
M12	PAHGV**T*(T)**SAPDTRPAPGS**T*(T)**A	M56	PAHGV**T*(C2T2$_{Hex}$)**SAPD**T*(C2T2$_{Hex}$)**RPAPGS**T*(C2T2$_{Hex}$)**A
M13	PAHGVTSAPD**T*(T)**RPAPGS**T*(T)**A	M57	PAHGV**T*(C3T1)**SAPDTRPAPGSTA
M14	PAHGV**T*(T)**SAPD**T*(T)**RPAPGS**T*(T)**A	M58	PAHGVTSAPD**T*(C3T1)**RPAPGSTA
M15	PAHGV**T*(C1T1)**SAPDTRPAPGSTA	M59	PAHGVTSAPDTRPAPGS**T*(C3T1)**A
M16	PAHGVTSAPD**T*(C1T1)**RPAPGSTA	M60	PAHGV**T*(C3T1)**SAPD**T*(C3T1)**RPAPGSTA
M17	PAHGVTSAPDTRPAPGS**T*(C1T1)**A	M61	PAHGV**T*(C3T1)**SAPDTRPAPGS**T*(C3T1)**A
M18	PAHGV**T*(C1T1)**SAPD**T*(C1T1)**RPAPGSTA	M62	PAHGVTSAPD**T*(C3T1)**RPAPGS**T*(C3T1)**A
M19	PAHGV**T*(C1T1)**SAPDTRPAPGS**T*(C1T1)**A	M63	PAHGV**T*(C3T1)**SAPD**T*(C3T1)**RPAPGS**T*(C3T1)**A
M20	PAHGVTSAPD**T*(C1T1)**RPAPGS**T*(C1T1)**A	M64	PAHGV**T*(C3T2)**SAPDTRPAPGSTA
M21	PAHGV**T*(C1T1)**SAPD**T*(C1T1)**RPAPGS**T*(C1T1)**A	M65	PAHGVTSAPD**T*(C3T2)**RPAPGSTA
M22	PAHGV**T*(C1T2)**SAPDTRPAPGSTA	M66	PAHGVTSAPDTRPAPGS**T*(C3T2)**A
M23	PAHGVTSAPD**T*(C1T2)**RPAPGSTA	M67	PAHGV**T*(C3T2)**SAPD**T*(C3T2)**RPAPGSTA
M24	PAHGVTSAPDTRPAPGS**T*(C1T2)**A	M68	PAHGV**T*(C3T2)**SAPDTRPAPGS**T*(C3T2)**A
M25	PAHGV**T*(C1T2)**SAPD**T*(C1T2)**RPAPGSTA	M69	PAHGVTSAPD**T*(C3T2)**RPAPGS**T*(C3T2)**A
M26	PAHGV**T*(C1T2)**SAPDTRPAPGS**T*(C1T2)**A	M70	PAHGV**T*(C3T2)**SAPD**T*(C3T2)**RPAPGS**T*(C3T2)**A
M27	PAHGVTSAPD**T*(C1T2)**RPAPGS**T*(C1T2)**A	M71	PAHGV**T*(C4T1)**SAPDTRPAPGSTA
M28	PAHGV**T*(C1T2)**SAPD**T*(C1T2)**RPAPGS**T*(C1T2)**A	M72	PAHGVTSAPD**T*(C4T1)**RPAPGSTA
M29	PAHGV**T*(C2T1$_{Tet}$)**SAPDTRPAPGSTA	M73	PAHGVTSAPDTRPAPGS**T*(C4T1)**A
M30	PAHGVTSAPD**T*(C2T1$_{Tet}$)**RPAPGSTA	M74	PAHGV**T*(C4T1)**SAPD**T*(C4T1)**RPAPGSTA
M31	PAHGVTSAPDTRPAPGS**T*(C2T1$_{Tet}$)**A	M75	PAHGV**T*(C4T1)**SAPDTRPAPGS**T*(C4T1)**A
M32	PAHGV**T*(C2T1$_{Tet}$)**SAPD**T*(C2T1$_{Tet}$)**RPAPGSTA	M76	PAHGVTSAPD**T*(C4T1)**RPAPGS**T*(C4T1)**A
M33	PAHGV**T*(C2T1$_{Tet}$)**SAPDTRPAPGS**T*(C2T1$_{Tet}$)**A	M77	PAHGV**T*(C4T1)**SAPD**T*(C4T1)**RPAPGS**T*(C4T1)**A
M34	PAHGVTSAPD**T*(C2T1$_{Tet}$)**RPAPGS**T*(C2T1$_{Tet}$)**A	M78	PAHGV**T*(C4T2)**SAPDTRPAPGSTA
M35	PAHGV**T*(C2T1$_{Tet}$)**SAPD**T*(C2T1$_{Tet}$)**RPAPGS**T*(C2T1$_{Tet}$)**A	M79	PAHGVTSAPD**T*(C4T2)**RPAPGSTA

M36	PAHGV**T*(C2T2$_{Tet}$)**SAPDTRPAPGSTA	M80	PAHGVTSAPDTRPAPGS**T*(C4T2)**A
M37	PAHGVTSAPD**T*(C2T2$_{Tet}$)**RPAPGSTA	M81	PAHGV**T*(C4T2)**SAPD**T*(C4T2)**RPAPGSTA
M38	PAHGVTSAPDTRPAPGS**T*(C2T2$_{Tet}$)**A	M82	PAHGV**T*(C4T2)**SAPDTRPAPGS**T*(C4T2)**A
M39	PAHGV**T*(C2T2$_{Tet}$)**SAPD**T*(C2T2$_{Tet}$)**RPAPGSTA	M83	PAHGVTSAPD**T*(C4T2)**RPAPGS**T*(C4T2)**A
M40	PAHGV**T*(C2T2$_{Tet}$)**SAPDTRPAPGS**T*(C2T2$_{Tet}$)**A	M84	PAHGV**T*(C4T2)**SAPD**T*(C4T2)**RPAPGS**T*(C4T2)**A
M41	PAHGVTSAPD**T*(C2T2$_{Tet}$)**RPAPGS**T*(C2T2$_{Tet}$)**A	M85	PAHGVTSAPD**T*(C1T2)**RPAPG**S*(Tn)T*(Tn)**APPA
M42	PAHGV**T*(C2T2$_{Tet}$)**SAPD**T*(C2T2$_{Tet}$)**RPAPGS**T*(C2T2$_{Tet}$)**A	M86	PAHGVTSAPD**T*(C3T2)**RPAPG**S*(Tn)T*(Tn)**APPA
M43	PAHGV**T*(C2T1$_{Hex}$)**SAPDTRPAPGSTA	M87	PAHGVTSAPD**T*(C2T2)**RPAPG**S*(Tn)T*(Tn)**APPA
M44	PAHGVTSAPD**T*(C2T1$_{Hex}$)**RPAPGSTA	--	

**ID**	**Mucin**	**Glycopeptide sequence**
M88	MUC4	TSSASTGHATPLPVDT
M89	MUC4	T**S*(T)**SA**S*(T)**TGHATPLPVDT
M90	MUC4	TSSAS**T*(T)**GHA**T*(T)**PLPVDT
M91	MUC4	**T*(T)**SSAS**T*(T)**GHA**T*(T)**PLPVDT
M92	MUC4	T**S*(Tn)**SA**S*(Tn)**TGHATPLPVDT
M93	MUC4	TSSAS**T*(Tn)**GHA**T*(Tn)**PLPVDT
M94	MUC4	**T*(Tn)**SSAS**T*(Tn)**GHA**T*(Tn)**PLPVDT
M95	MUC5B	ATPSSTPGTTHTP
M96	MUC5B	A**T*(T)**PSS**T*(T)**PGTTHTP
M97	MUC5B	A**T*(T)**PSSTPG**T*(T)**THTP
M98	MUC5B	A**T*(T)**PSS**T*(T)**PG**T*(T)**THTP

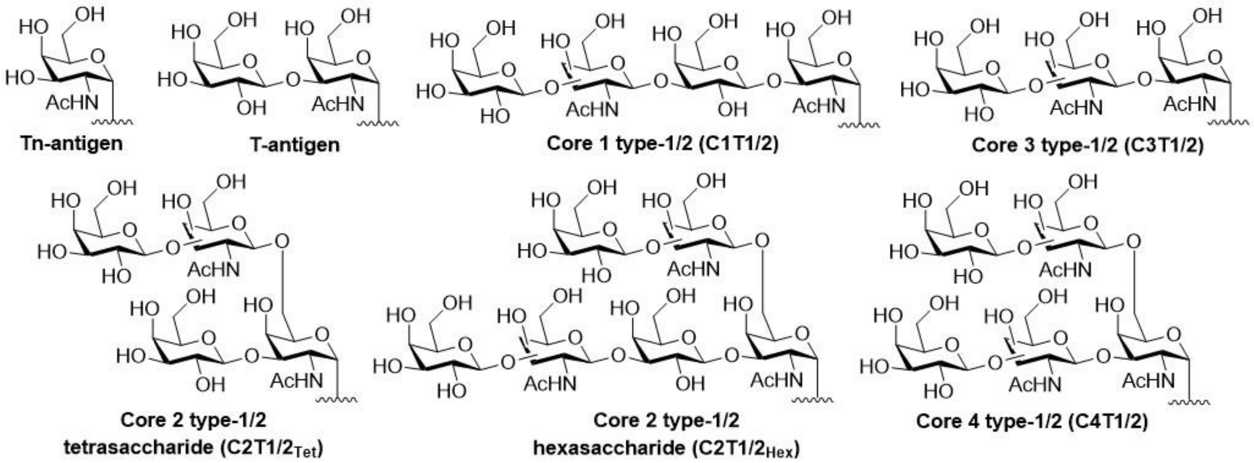

Figure S13. Mucin core structures.

6.2 Microarray binding studies with mouse sera

Incubations of slides were performed at 100 µL/well. The slides were incubated for 1 h at room temperature with mouse sera diluted 1:1000 in PBST (0.2 % Tween-20). Mouse antibodies were detected with a 1mg / ml goat anti-mouse Cy5 IgG (H+L) antibody (Invitrogen) diluted 1:500 in PBST (0.2 % Tween-20) and incubated for 1 h at room temperature. Finally, the microarray slides were scanned at 635 nm using a *GenePix 4300A* microarray fluorescence reader (Molecular Devices) and analyzed using *GenePix* Pro-7 software (Axon Instruments). Background was automatically subtracted by "spot edge average" background subtraction. A signal must have intensity higher than 3 times the background noise. The obtained data were analyzed using Excel (Microsoft). Reported signals represent the means of eight spot replicates

with standard deviations. Binding data for mouse sera obtained from immunization with **15** and **16** are shown in Figures S14-S17.

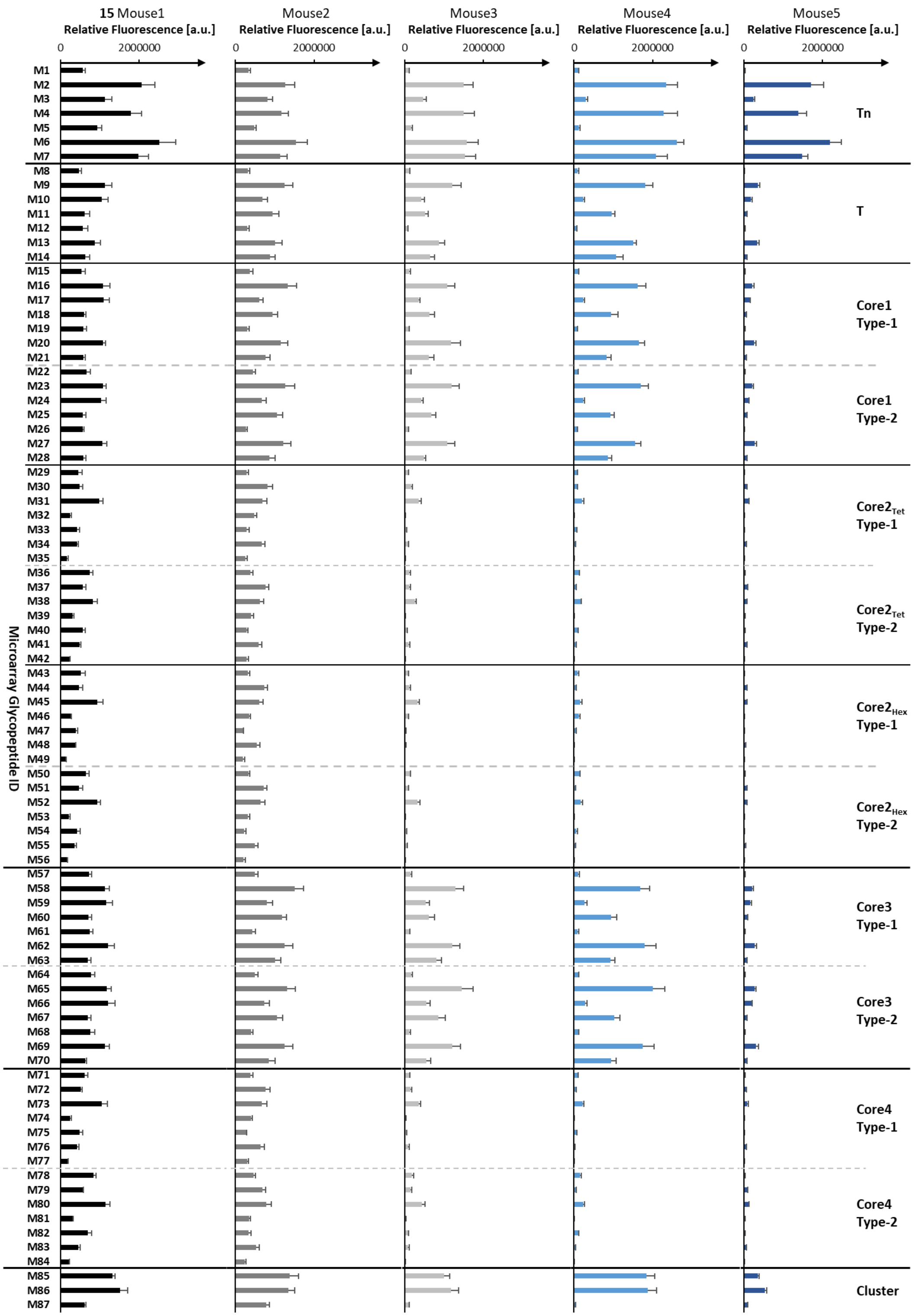

Figure S14. Binding of mouse sera derived from immunization with vaccine **15** to mucin core glycopeptides at 1:1000 dilution.

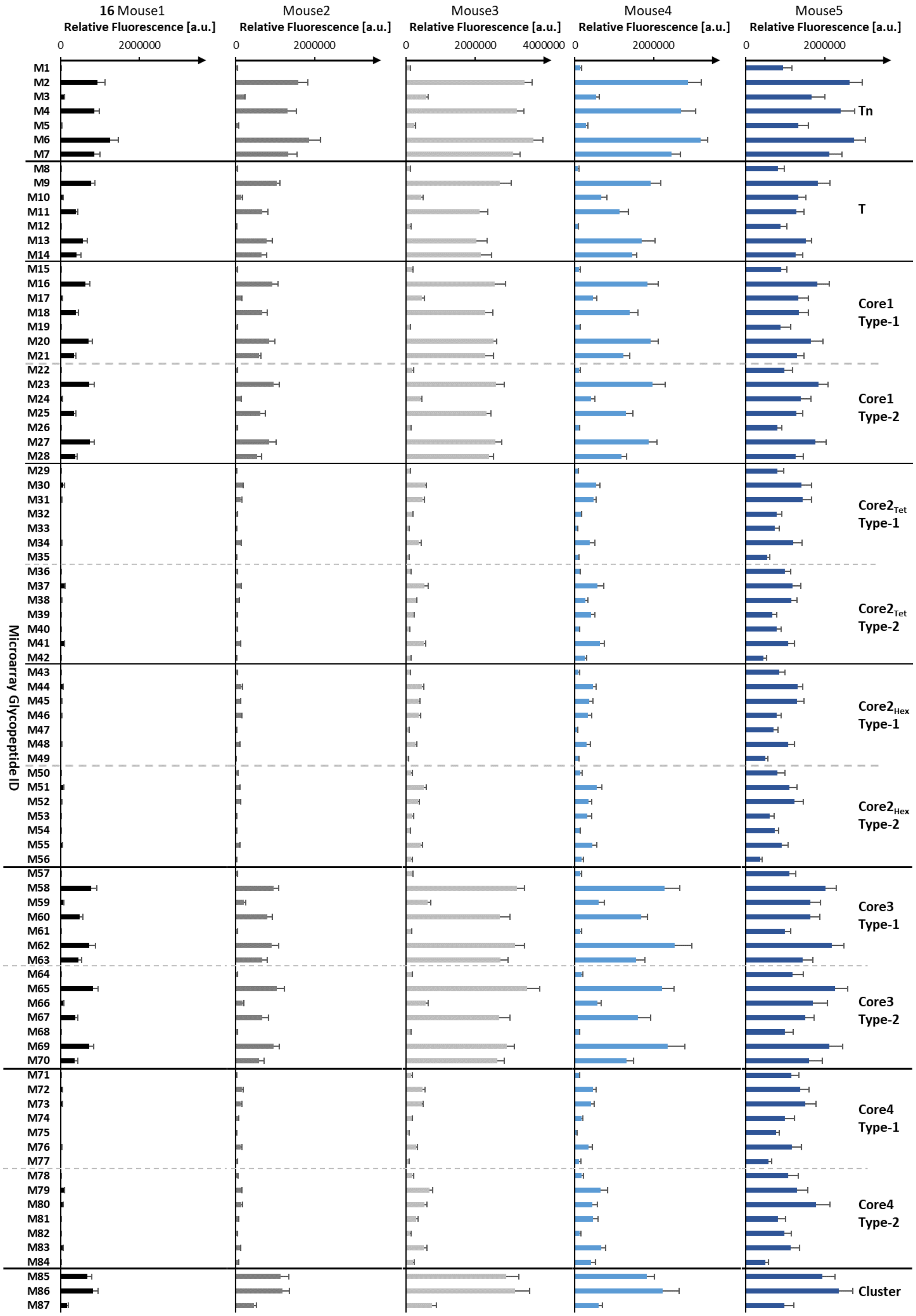

Figure S15. Binding of mouse sera derived from immunization with vaccine **16** to mucin core glycopeptides at 1:1000 dilution.

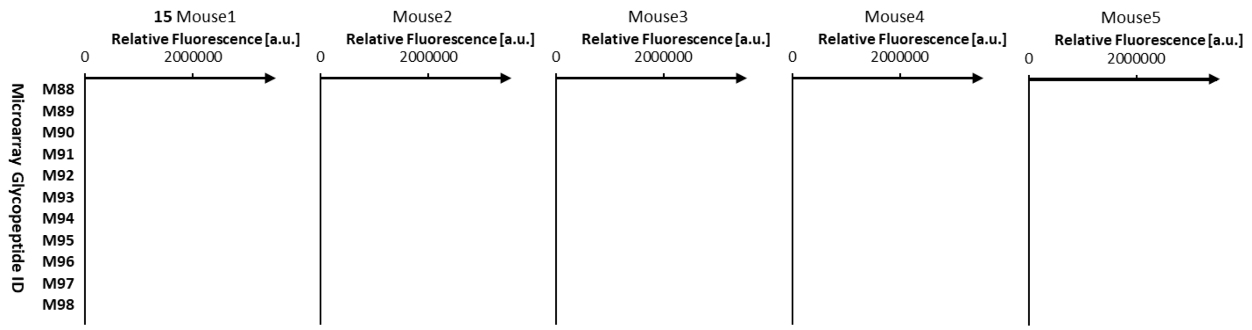

Figure S16. Binding of mouse sera derived from immunization with vaccine **15** to MUC4 and MUC5B glycopeptides at 1:1000 dilution.

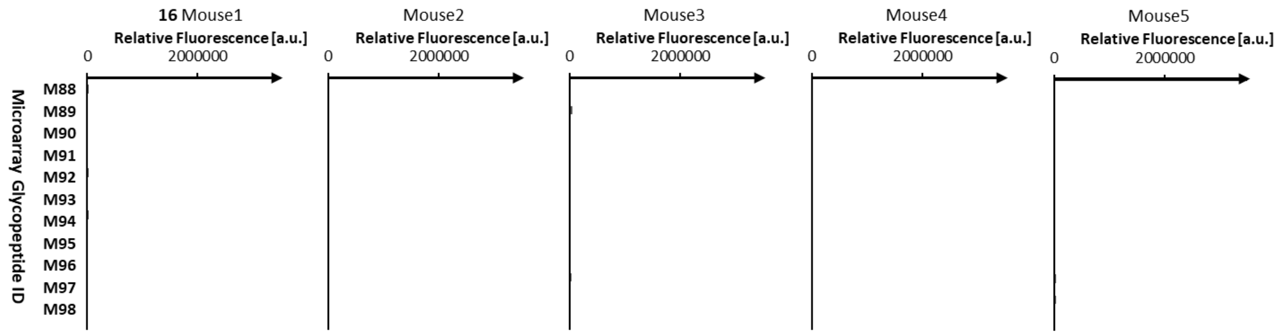

Figure S17. Binding of mouse sera derived from immunization with vaccine **16** to MUC4 and MUC5B glycopeptides at 1:1000 dilution.

7 References

(1) Gabba, A.; Bogucka, A.; Luz, J. G.; Diniz, A.; Coelho, H.; Corzana, F.; Cañada, F. J.; Marcelo, F.; Murphy, P. V.; Birrane, G. Crystal Structure of the Carbohydrate Recognition Domain of the Human Macrophage Galactose C-Type Lectin Bound to GalNAc and the Tumor-Associated Tn Antigen. *Biochemistry* **2021**, *60* (17), 1327–1336. https://doi.org/10.1021/acs.biochem.1c00009.

(2) Kaltner, H.; Manning, J. C.; García Caballero, G.; Di Salvo, C.; Gabba, A.; Romero-Hernández, L. L.; Knospe, C.; Wu, D.; Daly, H. C.; O'Shea, D. F.; Gabius, H. J.; Murphy, P. V. Revealing Biomedically Relevant Cell and Lectin Type-Dependent Structure-Activity Profiles for Glycoclusters by Using Tissue Sections as an Assay Platform. *RSC Adv.* **2018**, *8* (50), 28716–28735. https://doi.org/10.1039/c8ra05382k.

(3) Piñeiro, Á.; Muñoz, E.; Sabín, J.; Costas, M.; Bastos, M.; Velázquez-Campoy, A.; Garrido, P. F.; Dumas, P.; Ennifar, E.; García-Río, L.; Rial, J.; Pérez, D.; Fraga, P.; Rodríguez, A.; Cotelo, C. AFFINImeter: A Software to Analyze Molecular Recognition Processes from Experimental Data. *Anal. Biochem.* **2019**, *577* (February), 117–134. https://doi.org/10.1016/j.ab.2019.02.031.

(4) Pett, C.; Cai, H.; Liu, J.; Palitzsch, B.; Schorlemer, M.; Hartmann, S.; Stergiou, N.; Lu, M.; Kunz, H.; Schmitt, E.; Westerlind, U. Microarray Analysis of Antibodies Induced with Synthetic Antitumor Vaccines: Specificity against Diverse Mucin Core Structures. *Chem. - A Eur. J.* **2017**, *23* (16), 3875–3884. https://doi.org/10.1002/chem.201603921.

(5) Cai, H.; Palitzsch, B.; Hartmann, S.; Stergiou, N.; Kunz, H.; Schmitt, E.; Westerlind, U. Antibody Induction Directed against the Tumor-Associated MUC4 Glycoprotein. *ChemBioChem* **2015**, *16* (6), 959–967. https://doi.org/10.1002/cbic.201402689.
